# Wearable Sensors for Physiological Condition and Activity Monitoring

**DOI:** 10.1002/smsc.202300358

**Published:** 2024-05-18

**Authors:** Pillalamarri Srikrishnarka, Joonas Haapasalo, Juan P. Hinestroza, Zhipei Sun, Nonappa

**Affiliations:** ^1^ Faculty of Engineering and Natural Sciences Korkeakoulunkatu 6 FI‐33720 Tampere Finland; ^2^ Department of Neurosurgery Tampere University Hospital and Tampere University Kuntokatu 2 33520 Tampere Finland; ^3^ Department of Fiber Science and Apparel Design Cornell University Ithaca NY 14853 USA; ^4^ Department of Electronics and Nanoengineering Aalto University P.O. Box 13500 FI‐00076 Aalto Finland; ^5^ QTF Center of Excellence Department of Applied Physics Aalto University 00076 Aalto Finland

**Keywords:** biomarkers, condition monitorings, metabolites, wearable sensors

## Abstract

Rapid technological advancements have transformed the healthcare sector from traditional diagnosis and treatment to personalized health management. Biofluids such as teardrops, sweat, interstitial fluids, and exhaled breath condensate offer a rich source of metabolites that can be linked to the physiological status of an individual. More importantly, these biofluids contain biomarkers similar to those in the blood. Therefore, developing sensors for the noninvasive determination of biofluid‐based metabolites can overcome traditionally invasive and laborious blood‐test‐based diagnostics. In this context, wearable devices offer real‐time and continuous physiological conditions and activity monitoring. The first‐generation wearables included wristwatches capable of tracking heart rate variations, breathing rate, body temperature, stress responses, and sleeping patterns. However, wearable sensors that can accurately measure the metabolites are needed to achieve real‐time analysis of biomarkers. In this review, recent progresses in wearable sensors utilized to monitor metabolites in teardrops, breath condensate, sweat, and interstitial fluids are thoroughly analyzed. More importantly, how metabolites can be selectively detected, quantified, and monitored in real‐time is discussed. Furthermore, the review includes a discussion on the utility of, multifunctional sensors that combine metabolite sensing, human activity monitoring, and on‐demand drug delivery system for theranostic applications.

## Graphical abstract

Recent progress in wearable sensors for noninvasive sensing of teardrop, breath, and sweat metabolites is reviewed. The importance of wearable sensors for real‐time human activity and health monitoring, clinical significance, and opportunities for personalized diagnosis and treatment are highlighted.

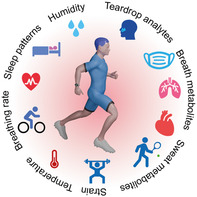

## 1 Introduction

According to the World Health Organization (WHO), healthcare expenditures in the past two decades have skyrocketed to 11.9% (≈$11 trillion) of the global gross domestic product (GDP).^[^ ^1^ ^]^ The European Union (EU) spends approximately 8.1% (€1.18 billion) of its GDP on public healthcare, of which 1.2% is spent on medical products, appliances, and equipment.^[^ ^2^ ^]^ Understanding these spending patterns is vital for policymakers and healthcare providers to maximize the services needed, as human resources and facilities are finite.^[^ ^3^, ^4^, ^5^, ^6^ ^]^ Conventional medical diagnostic methods include invasive routes of testing and analysis that sometimes require sophisticated equipment. These factors limit easy access to primary healthcare and diagnostic services for most of the population. Invasive blood‐test‐based diagnostics are unsuitable for certain individuals.^[^ ^7^ ^]^ For example, in elderly people, blood sampling may be challenging due to thinner skin and fragile blood vessels. Similarly, infants have an immature immune system, fragile blood vessels, and low blood volume. Furthermore, blood‐based diagnostics do not allow for real‐time and continuous monitoring of the human physiological condition and activity. This situation warrants the need for lightweight, low‐cost, and efficient noninvasive diagnostic tools. Such tools must offer real‐time monitoring of patients’ pathological states and provide crucial insights for preventive diagnosis and personalized treatments. Recent advances in material science, electronics, and nanotechnology have enabled the fabrication of lightweight and flexible sensors as wearable devices that can continuously monitor vital features in a noninvasive manner.^[^ ^8^ ^]^

Wearable devices incorporate analytical sensors that can be worn on the body or integrated into clothing. Being a part of the internet of things (IoT), wearable sensors can perform functions such as tracking human activity, monitoring the health condition of the user and transferring data in real time.^[^ ^9^ ^]^ Such devices can rapidly provide baseline values and issue alerts on plausible abnormalities in the monitored data. Advanced and miniaturized wearable sensors such as smart watches, earbuds, and smart glasses are able to provide continuous information on the physiological state of patients.^[^ ^10^ ^]^ For example, wearable sensor‐integrated watches can measure key biometrics such as oxygen saturation, heart rate, and breathing rates.^[^ ^11^ ^]^ The availability of these advanced sensors has enabled healthcare professionals to monitor physiological parameters for early and preventive treatments. Recent efforts include integrating sensors inside wearable devices for monitoring changes in the concentrations of biomarkers present in a wide range of biofluids.

It is feasible that some of these noninvasive devices can be further integrated with targeted drug delivery systems, leading to the use of theranostic devices.^[^ ^12^ ^]^ Most wearable devices are powered by batteries that may require frequent charging, which is a major challenge for long‐term monitoring. Significant research has been done on self‐powered wearable sensors capable of harnessing the mechanical motion of the user and converting it into electric potential to power these wearable devices.^[^ ^13^ ^]^ Triboelectric, piezoelectric, and thermoelectric generators can translate mechanical and thermal energy into electrical energy.^[^ ^14^ ^]^

**Figure 1:**
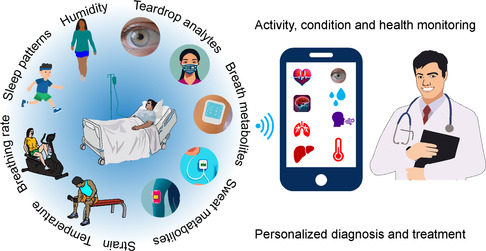
A schematic of the noninvasive metabolite sensing and human activity monitoring discussed in this review.

In this review, we will briefly discuss the evolution of wearable sensor technology and sensor fabrication parameters. We will focus on wearable sensors to monitor teardrop metabolites, breath metabolites, epidermal sweat metabolites, and interstitial fluids (**Figure** **1**). Using selected examples, we will discuss how metabolites such as glucose, lactose, carbon dioxide, ammonia, acetone, pH, ions, hydrogen peroxide, lipids, and matrix metalloproteinase‐9 (MMP‐9) can be monitored using wearable sensors. Furthermore, we will present how parameters such as intraocular pressure, temperature, breath rate, sweat rate, and humidity can also be tracked during specific human activities. Beyond sensing, wearable sensors integrated with microneedle (MN) platforms can also offer selective detection and simultaneous drug delivery for theranostic applications.^[^ ^15^ ^]^ Finally, we will conclude with the limitations and future opportunities for wearable sensor technologies.

## 2 Evolution of Wearable Sensors

**Figure 2:**
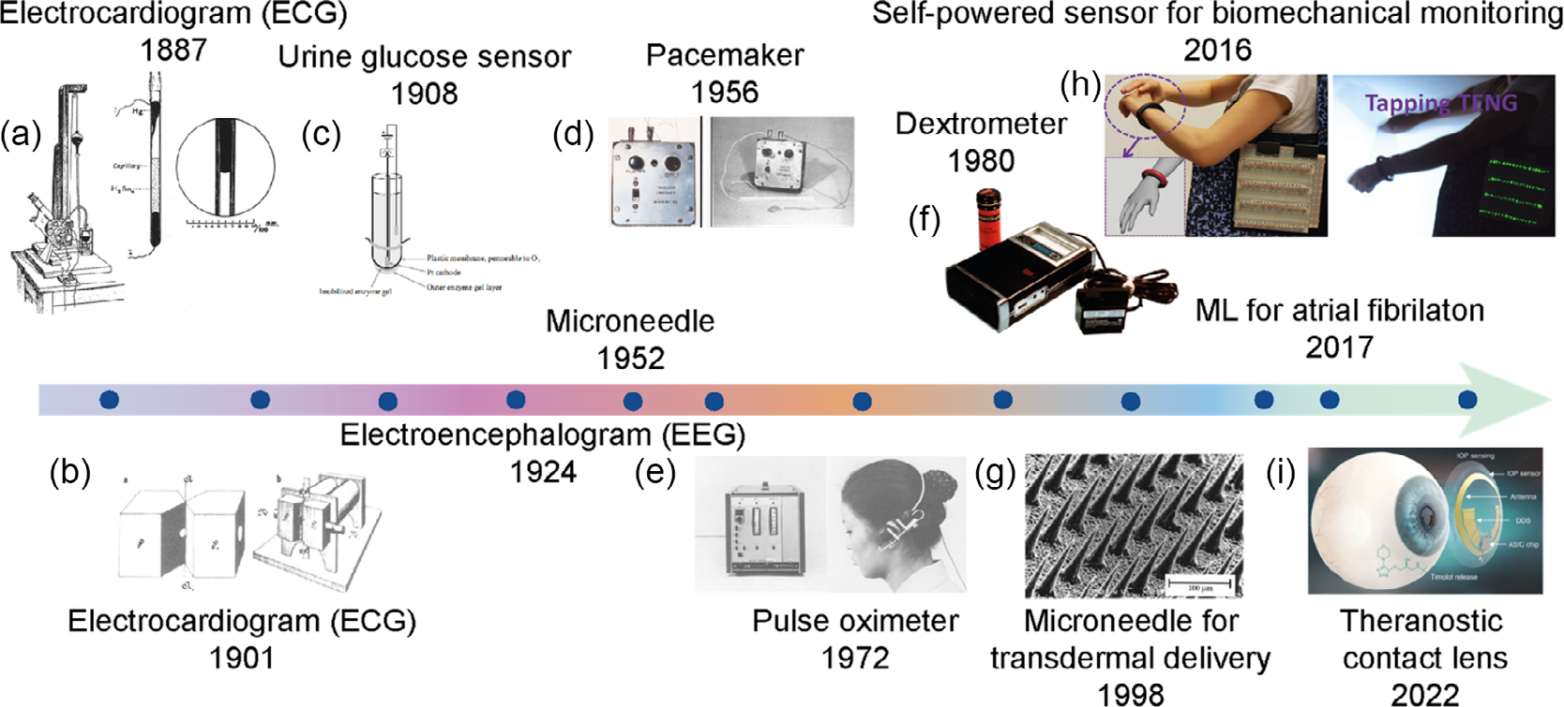
The evolution of wearable sensors. Selected examples of a) capillary electrometer. Reproduced with permission.^[^ ^17^ ^]^ Copyright 2000, American College of Cardiology. Published by Elsevier Inc. b) Electrocardiogram. Reproduced with permission.^[^ ^16^ ^]^ Copyright 2003, Kluwer Academic Publishers. c) Glucose sensor. Reproduced with permission.^[^ ^21^ ^]^ Copyright 2015, Elsevier Inc. d) Photograph of pacemaker. Reproduced with permission.^[^ ^25^ ^]^ Copyright 2006, Paediatric Cardiology under CC 3.0. e) Photograph of a volunteer wearing a pulse oximeter. Modified with permission.^[^ ^26^ ^]^ Copyright 1987, Little, Brown and Company, Inc. f) The photograph of a dextrometer. Reproduced with permission.^[^ ^23^ ^]^ Copyright 2011, SAGE Publications. g) Scanning electron microscopy (SEM) image of the microneedle array. Reproduced with permission.^[^ ^37^ ^]^ Copyright 1998, Wiley‐Liss, Inc. and the American Pharmaceutical Association. h) A self‐powered loop for energy harvesting. Adapted under the terms of the Creative Commons Attribution 4.0 International License.^[^ ^29^ ^]^ Copyright 2016, The Author(s). Published by the American Association for the Advancement of Science. i) Schematic representation of a theranostic contact lens. Adapted under the terms of the Creative Commons Attribution 4.0 International License.^[^ ^33^ ^]^ Copyright 2022, The Author(s). Published by Springer Nature.

Wearable sensors can be classified into invasive and noninvasive based on their interaction with the human body. **Figure** **2** shows a summary of the evolution and development of wearable sensors. The first electrocardiogram (ECG) was measured by Augustus Waller in 1887 using a mercury capillary electrometer from an intact human heart, although only two distorted deflection tracings were reported.^[^ ^16^ ^]^ Willem Einthoven is regarded as the founding father of the modern ECG. In 1901, he introduced a string galvanometer to register these distortions.^[^ ^17^ ^]^ Richard Caton detected the electrical properties of the brain for the first time by recording electrical activity in an animal brain using a galvanometer.^[^ ^18^ ^]^ In 1924, Hans Berger recorded the first electroencephalogram (EEG).^[^ ^19^ ^]^ In 1956, Leland C. Clark measured the concentration of oxygen in blood using electrodes.^[^ ^20^ ^]^ Benedict reported the first noninvasive urine glucose sensing in 1908, when he used a copper reagent to detect glucose in urine.^[^ ^21^ ^]^ Benedict's reagent includes a complex mixture of sodium carbonate, sodium citrate, and copper (II) sulfate pentahydrate and is commonly used for detecting reducing sugars. In 1965, Ames developed dextrostix, the first blood glucose strip based on glucose oxidase.^[^ ^22^ ^]^ A color change was detected upon interacting with the glucose in the blood. By comparing the color change to a standard chart, glucose concentration in the blood can be assessed semiquantitatively. With further improvements, the first domestic glucose meter, the dextrometer, was launched in 1980 for self‐monitoring.^[^ ^23^ ^]^ Today more than 537 million people between the ages of 20 and 79 years are living with diabetes around the world.^[^ ^24^ ^]^ Notably, 75% of them are living in low‐ and middle‐income countries. Portable devices for blood glucose monitoring have positively affected patients diagnosed with type 1 diabetes.

Implantable devices are another class of wearable devices introduced into the human body, permanently or temporarily, through surgical or other medical interventions. These implantable devices are aimed at supporting the correct function of specific organs or tissues. Beyond that, they also allow for real‐time monitoring, providing clinically significant data for improved patient care. In the 1950s, several landmark developments were made in the area of implantable devices, such as pacemakers. In 1957, Earl E. Bakken fabricated a battery‐powered pacemaker with knobs for controlling both stimulus rate and amplitude. This pacemaker prototype was tested on a dog, and upon positive results, it was used to treat a young girl the next day.^[^ ^25^ ^]^ The first pacemaker was already in clinical use within 4 weeks of this experiment. Today, over one million pacemakers are implanted each year worldwide. Takuo Aoyagi and Michio Kishi developed the first pulse oximeter in 1972 to measure the saturation of blood oxygen concentration. The device was commercialized in 1977 by Minolta in Japan and in the US by Biox in 1980.^[^ ^26^ ^]^ This device used the ratio of absorption intensities of light from the red to the infrared region of the electromagnetic spectrum during each pulsation and correlated this ratio to the oxygen level.

Soft contact lenses are another class of wearable materials that can potentially gain insights into teardrop metabolites. Gaylord et al. reported the first oxygen‐permeable soft contact lens for vision correction.^[^ ^27^ ^]^ In 2013, Andoralove et al. used a soft contact lens as a substrate for sensing many biomarkers in tears.^[^ ^28^ ^]^ Rogers and co‐workers reported one of the early epidermal sensors, which wirelessly transmitted the surface temperature of the skin, ECG, and strain.^[^ ^29^ ^]^ In 2016, Guder et al. demonstrated a paper substrate for measuring exhale breath humidity.^[^ ^30^ ^]^ A self‐powered wearable sensor was first reported by Yi et al. in 2016.^[^ ^31^ ^]^ In 2017, Shashikumar et al. reported the incorporation of machine learning (ML) algorithms in wearable technology to detect atrial fibrillation.^[^ ^32^ ^]^ Kim et al. reported the fabrication of the first theranostic smart contact lens to detect and treat glaucoma.^[^ ^33^ ^]^ MN‐based platforms are another class of biomedical devices for the extraction, detection, and monitoring of biomarkers.^[^ ^15^ ^]^ Because of their minimally invasive nature, MNs are a promising alternative for hypodermic needle‐based delivery.^[^ ^34^ ^]^ In 1952, Reaume reported the fabrication of first glass MNs using hydrofluoric acid.^[^ ^35^ ^]^ Three decades later, Hashmi et al. used an MN‐based device to inject deoxyribonucleic acid (DNA) through the nematode cuticle.^[^ ^36^ ^]^ In 1998, Henry et al. reported the first transdermal drug delivery using an MN array.^[^ ^37^ ^]^ Recently, Ates et al. categorized wearable sensor technologies into first and second generations.^[^ ^38^ ^]^ Sensors that monitor physical activities such as heart rate and body temperature are grouped under first‐generation sensor technologies, whereas sensors that analyze biofluids are grouped as second‐generation sensors.

## 3 Fabrication of Wearable Sensors

Fabrication of sensor‐integrated wearable devices is a complex process involving various steps. Typically, a wearable device consists of receptors (recognition units) for metabolites, a sampling unit, a sensing unit, a signal transduction module, a signal amplification module, a power supply, and a data transfer unit. In this section, we will briefly summarize the importance of receptors and sampling, sensing elements, substrate selection, transduction techniques, and different steps involved in the fabrication of wearable sensors.

### 3.1 Receptors and Sampling

Biofluids such as interstitial fluids, teardrops, sweat, breath condensates, urine, and saliva are major sources of metabolites.^[^ ^39^ ^]^ Accordingly, wristwatches, skin patches, tattoos, face masks, contact lenses, fitness bands, and diapers can be integrated with sensors and sampling units without altering their original purpose.^[^ ^40^, ^41^ ^]^ However, appropriate sampling and metabolite‐specific sensors need to be incorporated to achieve this goal. The success of noninvasive metabolite detection and quantification depends on the sensitivity, specificity, compatibility, and output modality of the receptors. Enzymes, aptamers, affinity proteins, custom designed peptides, synthetic imprinted polymers, or surface‐functionalized nanomaterials can be used as receptors as well as sensing elements.^[^ ^42^ ^]^ Biological receptor molecules can selectively bind to a biomarker with high sensitivity and specificity and perform a catalytic reaction. These receptors are chemically or physically immobilized on the surface of the transducers. Accordingly, when they interact with analytes, they generate an appropriate signal (chemical, optical, or electrical) that can correlate with the metabolite concentration. The volume of the biofluids and concentration of metabolites vary from organ to organ and among individuals, so MNs, microfluidics, immersion, and direct contact methods are used to sample the biofluids.^[^ ^43^, ^44^ ^]^

### 3.2 Sensing Elements

The key aspect of a sensing element is the material used, which needs to be specific to the analyte, exhibit a quick response and recovery time, and be robust and affordable. We classified the materials reported in the literature into the following four categories: metal and metal oxides, conducting polymers, dyes, and carbon materials (**Table** **1**).

**Table 1:** List of commonly used sensing materials, selected examples, and their properties.

Materials	Examples	Fabrication scalability	Biocompatibility	Sustainability
Metals and metal oxides	CuO, Cu_2_O, SnO, SnO_2_, ZnO, NiO, In_2_O_3_, WO_3_, Au, Ag, Cu, Pt	Moderate	Moderate	Minimal
Conducting polymers	Poly (3, 4‐ethylenedioxythiophene: polystyrene sulfonate) PEDOT:PSS, polyaniline, polypyrrole	Moderate	Minimal	Minimal
Dyes and nanoparticles	pH indicators, photonic crystals, thermochromic dyes, liquid crystals, plasmonic nanoparticles	Good	Minimal	Minimal
Carbon nanomaterials	Graphene, graphene oxide, carbon nanotubes (CNTs), carbon nanofibers	Moderate	Moderate	Minimal

Precious metals such as gold, silver, copper, and platinum have been extensively used as electrodes and components for inks, pastes, and substrates to detect analytes in electrochemical sensors.^[^ ^45^ ^]^ Owing to their excellent electrical properties, flexibility and usability make them viable for the large‐scale production of sensors.^[^ ^46^ ^]^ However, due to the high costs, the large‐scale production of these materials can be challenging. Metal oxide nanomaterials have electronic properties that can be tuned between conducting, semiconducting, and insulating behavior. Additionally, they can be surface functionalized via chemical modification to improve their selective sensing of analytes. Metal oxides also allow for easy fabrication and display rapid response, recovery times, excellent stability, and reproducibility.^[^ ^47^, ^48^, ^49^ ^]^ One of the major drawbacks of these materials is the high processing temperatures, which increases the overall cost of manufacturing. Some sensing applications require higher operation temperatures, which requires a pragmatic approach for different fields. Recently, conducting polymers have been extensively used in wearable sensors as they possess high flexibility, stretchability, and tunable conductivity based on the concentration of dopants. Additionally, their stability, lightweight, and low fabrication costs make them highly sought‐after materials for wearable technologies.^[^ ^50^, ^51^ ^]^ However, many conducting polymers suffer from poor recyclability and low selectivity, making them less sustainable, and new research is being pushed toward green synthesis routes.^[^ ^52^ ^]^

Dye molecules have excellent specificity and dynamic response time, and require simple processing steps to fabricate a sensor. Dyes can offer instrument‐free analysis. However, dye molecules can be expensive and display limited stability, which hampers their overall usability in large‐scale production of wearable sensors. Graphene oxide and reduced graphene oxides (rGOs) have been extensively reported in the fabrication of flexible electronics and wearable sensors due to their mechanical strength, flexibility, and electrical conductivity.^[^ ^53^, ^54^, ^55^, ^56^ ^]^ One of the major limitations of using graphene and graphene oxide in wearable sensors is the expensive and laborious synthesis steps. Carbon nanotubes (CNTs) are 1D nanomaterials with a varying electronic nature ranging from conducting to insulating.^[^ ^57^ ^]^ Metallic CNTs have quick electron transport kinetics and are typically used as working electrodes for three‐electrode electrochemical sensors.^[^ ^58^ ^]^ In contrast, semiconducting CNTs are used as electrodes for field‐effect transistor (FET)‐based sensors.

### 3.3 Substrate Selection

Substrates play a vital role in wearable sensors, and substrate selection depends on several operating constraints. Substrate materials must be flexible, breathable, mechanically tough, elastic, and long‐lasting. In addition to their physical and mechanical properties, the substrate must be bioinert and biocompatible. Substrate materials can be broadly classified into inorganic materials and polymers. Metals and semiconductors are classified as inorganic materials, possessing one of the highest electric conductivity.^[^ ^59^ ^]^ Processing these metals into desirable forms is possible due to their high ductility. Therefore, they can be used to fabricate conducting inks, threads, and serpentine patterns that can be transformed into conformable wearable sensors.^[^ ^60^, ^61^ ^]^ Some of the electronic components needed for miniaturization can be printed on flexible membranes for transforming into a wearable device. However, one of the major drawbacks of metal‐based wearable sensors is the potential lack of biocompatibility, which restricts their use in applications that require intimate contact with the user. Synthetic and natural polymer‐based hydrogels offer flexible, comfortable, soft, biocompatible and easily processable substrates. Polymers have been used in fabrics that can be tuned to impart hydrophobicity and increased breathability. Because of their biocompatibility, hydrogel‐based substrates have been extensively used as scaffolds for implantable materials.

### 3.4 Transduction Techniques

Wearable sensors can use electrochemical or colorimetric sensing methods for signal transduction. Electrochemical sensors can convert a chemical reaction into an electrical signal.^[^ ^62^ ^]^ In electrochemical sensors, chemical reactions often occur at the electrodes where the analyte undergoes oxidation or reduction.^[^ ^42^ ^]^ A redox chemical reaction implies changes in voltage, impedance, or current proportional to the concentration of the analyte. In electrochemical sensors, a change in color can also be used to indicate the presence of specific targeted analytes and the intensity of the color is proportional to the concentration of the analyte. Electrochemical sensors are portable, easy to fabricate, and do not need sophisticated instruments for further analysis.^[^ ^63^ ^]^ In the following sections, we will summarize the use of different wearable sensors to monitor vital biomarkers in teardrops, exhaled breath condensate, sweat and interstitial fluids.

## 4 Teardrop Metabolite Sensing

**Figure 3:**
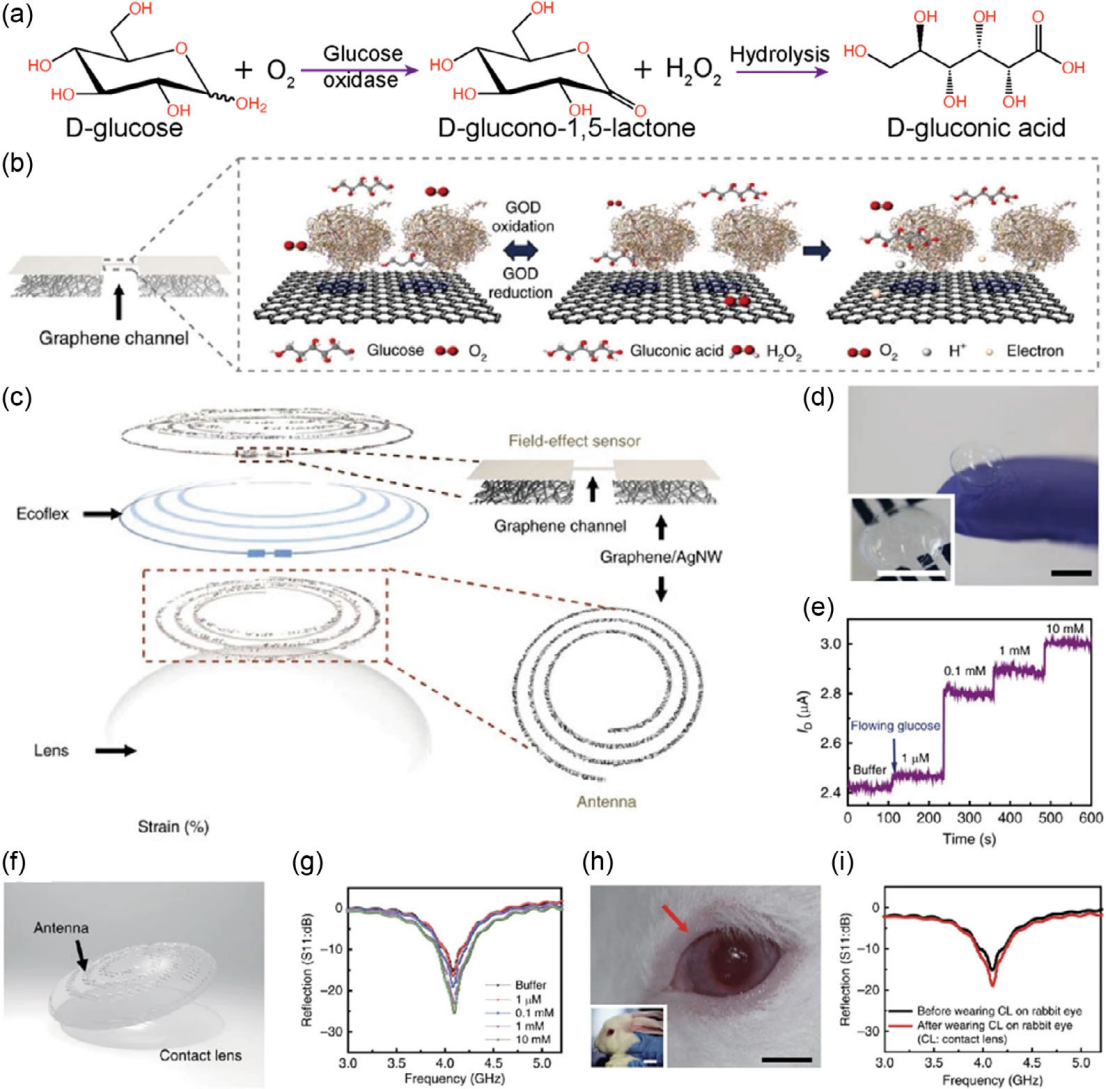
Smart contact lens for glucose sensing. a) The mechanism of glucose oxidation in the presence of glucose oxidase. b) Schematics of graphene‐based FET and the principle of glucose sensing. c) Schematics of the sensor incorporated a smart contact lens for glucose and intraocular pressure sensing. d) Photograph of sensor‐integrated contact lens (scale bar 1.0 cm). e) The change in the drain current as a function of glucose concentration. f) Photograph of contact incorporated with an antenna. g) Wireless monitoring of glucose concentrations. h) Photographs of the eyes of a live rabbit with a smart contact lens. Scale bars, 1 cm (black) and 5 cm (white). i) Wireless glucose‐concentration‐sensing curves before and after wearing contact lenses on the eye of a live rabbit. (b–i) Reproduced under the terms of the Creative Commons Attribution 4.0 International License.^[^ ^73^ ^]^ Copyright 2017, The author(s). Published by Springer Nature.

Contact lenses have been widely used for vision correction and cosmetic purposes. As contact lenses are in continuous contact with the eyeball and the surrounding tear fluid, they are ideal substrates for incorporating flexible electronics and transforming them into wearable sensors. Tear fluid contains abundant proteins and other components similar to those found in the blood. Unlike invasive blood‐based assays, teardrop analysis offers a unique insight into the individual's physiological state through a noninvasive technique.^[^ ^64^, ^65^, ^66^, ^67^, ^68^, ^69^, ^70^, ^71^, ^72^ ^]^ Glucose is one of the important biomarkers present in teardrop fluid. Recent studies have revealed a correlation between glucose in blood and tear glucose concentrations. Measuring glucose concentration has been achieved using photonic microstructures, fluorescent sensors, and electrochemical sensors. In an early attempt to detect glucose concentration in the teardrop fluid, Kim et al. fabricated a transparent and stretchable real‐time glucose sensor incorporating an FET on a contact lens.^[^ ^73^ ^]^ Their design allowed real time, wireless detection of glucose in vivo and in vitro intraocular pressure monitoring. To achieve the sensing, a resistance, inductance, and capacitance circuit was integrated using a graphene–silver nanowire hybrid structure (**Figure** **3**). This hybrid network acts as a source/drain (S/D) electrode for an FET and the graphene behaves as a channel (Figure 3b,c). The incorporation of graphene–silver nanowire hybrid materials allowed for the retention of the optical transparency of the lens (Figure 3d). For glucose sensing, glucose oxidase was immobilized on the graphene using a pyrene linker. Glucose oxidase catalyzes glucose into gluconic acid and produces hydrogen peroxide (H_2_O_2_). H_2_O_2_ oxidizes to produce oxygen, protons, and electrons (Figure 3a,b). The resulting electron thereby increases the drain current, which is linearly related to the concentration of glucose (Figure 3e). The device allowed the detection of glucose concentrations as low as 1.0 μM.

**Figure 4:**
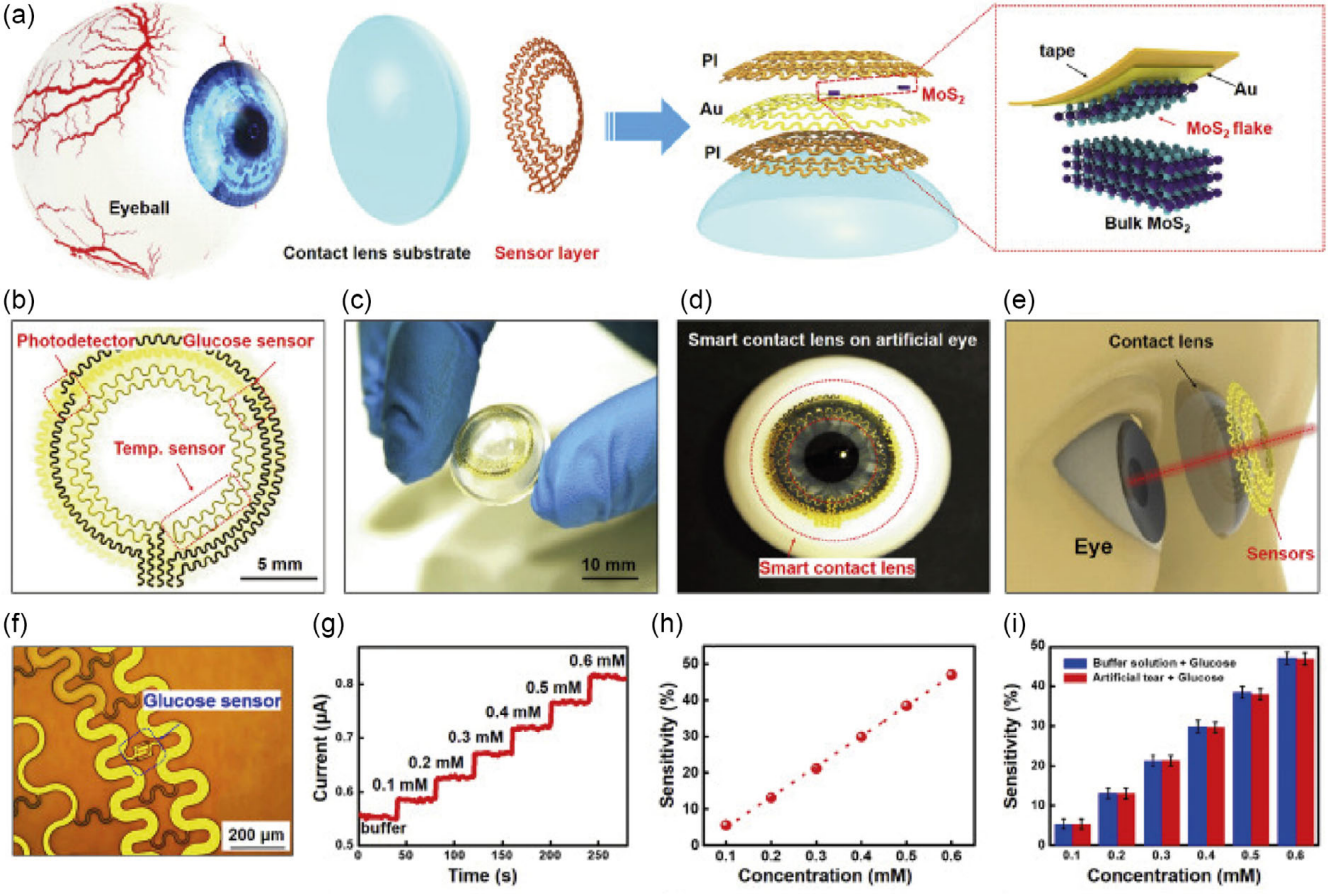
Multifunctional MoS_2_ transistor integrated with a contact lens. a) Schematics of eyeball, smart contact lens design, and the components for glucose sensing. b) Optical image of the serpentine electrode and sensor structure. c) Photograph of the sensor layer transferred onto a polydimethylsiloxane (PDMS) substrate. d) Photograph of an artificial eye with smart contact lens. e) Schematics of smart contact lens compared with human eye. f) Optical image of MoS_2_‐based glucose sensor. g) Change in the current as a function of time while changing the glucose concentration. h) The sensor sensitivity is measured as a function of glucose concentration. i) Sensitivity of the sensors with the phosphate buffered saline buffer solution (blue) and the artificial tear solution (red). Reproduced with permission.^[^ ^74^ ^]^ Copyright 2021, Elsevier Inc.

The contact lens was coupled with an external antenna, allowing wireless transmission of the data (Figure 3f). The sensor was specific to glucose even in the presence of interfering analytes such as lactic acid, ascorbic acid, and uric acid. For in vivo testing, the lens was incorporated into a rabbit eye and the glucose concentration was monitored for 5 h (Figure 3h,f). Transition metal dichalcogenides such as MoS_2_ have emerged as important sensing components due to their large surface‐to‐volume ratio, layered structure, tunable bandgap, and charge carrier properties. Guo et al. demonstrated a MoS_2_‐based sensor integrated into a contact lens to monitor glucose, and temperature (**Figure** **4**).^[^ ^74^ ^]^ To achieve this, MoS_2_ transistors were reinforced using photo‐patternable polyimide (PI). The elastic components were made of serpentine gold and PI (Figure 4a). Placing the sensor on the outer ring of the cornea prevented mechanical damage and unwanted blocking of the user's vision. Similar to the work by Kim et al. for glucose sensing, glucose oxidase was immobilized on the MoS_2_ surface. The Au‐based serpentine structure was used for temperature sensing (Figure 4b). By measuring the electrical resistance as a function of temperature, a sensitivity of 0.94 Ω °C^−^^1^was achieved with negligible changes due to mechanical strain. Overall, the sensor‐integrated contact lens allowed for detecting glucose concentrations <0.1 mM and photodetection with a responsivity of 4.8 A W^−1^ (Figure 4f–i). Electrochemical sensors for glucose monitoring require sophisticated manufacturing processes and a direct or wireless external power supply. These limitations can be overcome using optical spectroscopy.^[^ ^75^ ^]^ Kim et al. developed a biocompatible nanoparticle (NP)‐embedded contact lens that served as a glucose sensor. This sensor was fabricated by coating cerium oxide (III) and glucose oxidase on the contact lens. The glucose in the tear fluid was monitored by measuring the reflectance spectra of the sensor‐integrated lens. Lin et al. attempted to fabricate an organic solar cell integrated self‐powered sensor to overcome the challenges associated with the need for an external power source.^[^ ^76^ ^]^ The organic electrochemical transistor‐based sensor was designed to detect glucose and Ca^2+^ ions in the tear fluid. The sensor fabrication involved coating a membrane with a PEDOT:PSS channel for selective Ca^2+^ monitoring. A platinum gate was modified using a mixture of chitosan and glucose oxidase for glucose sensing. Glucose oxidase catalyzed the reaction producing gluconic acid and H_2_O_2_. In the presence of a positive bias, the Pt electrode catalyzes the electrochemical oxidation of H_2_O_2_. The oxidation process transfers electrons to the gate electrode and changes the electrical double layer at the gate/electrolyte interface.

**Figure 5:**
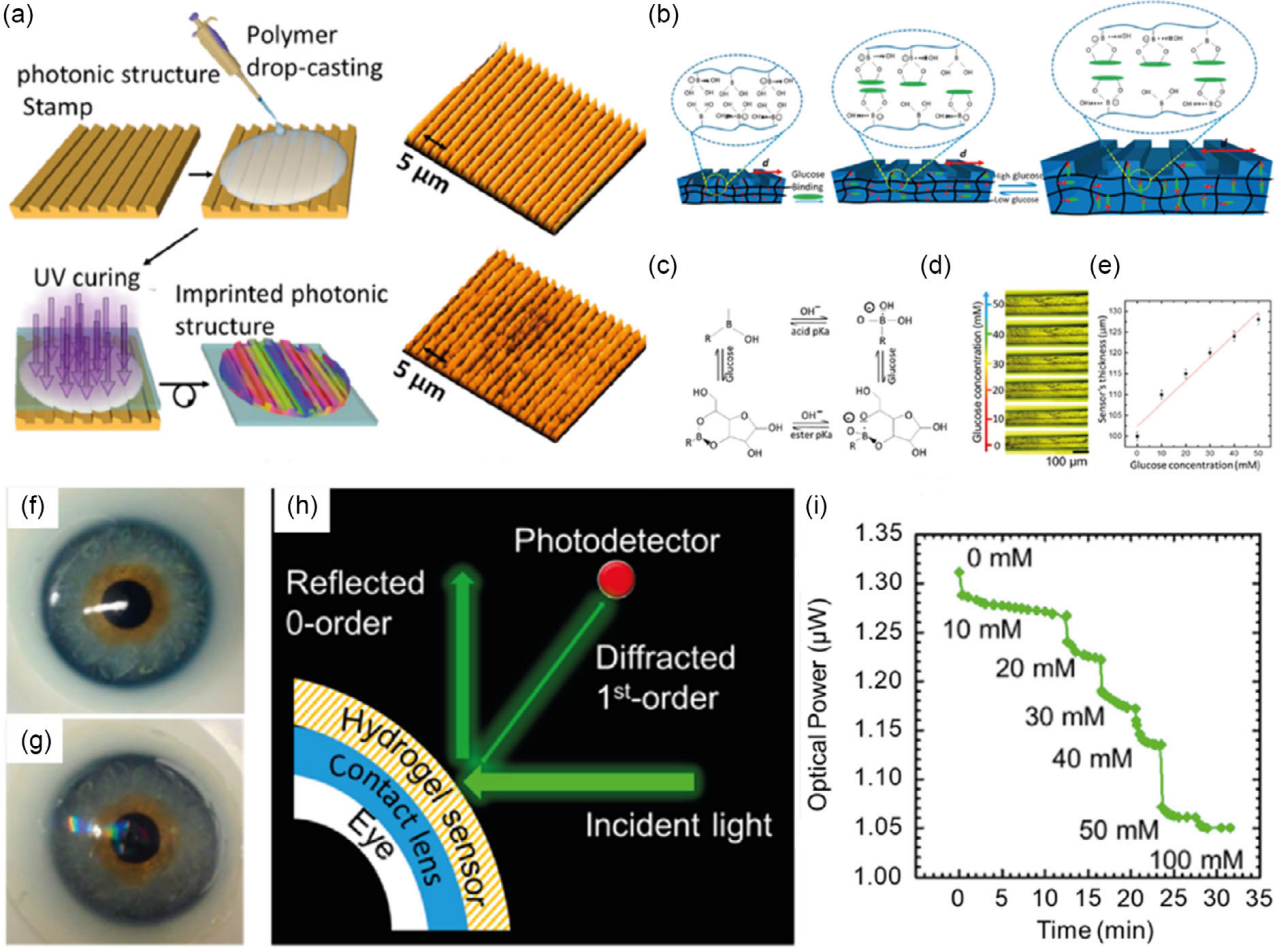
Photonic structures for glucose sensing. a) Schematics of photonic structure fabrication and optical microscope images. b) The complexation and equilibrium between glucose and phenylboronic acid. c) Effect of the Au‐phenylboronic acid complexation in the one‐dimensional photonic structure sensor. d) Optical microscope images showing the effect of glucose concentration on the photonic structures. e) Graph showing the change in the sensor's thickness as a function of glucose concentration. f) Photograph of a commercial contact lens on the artificial eye model. g) A photograph of the sensor attached to the contact lens was placed on the eye model. h) Schematic illustration showing the experimental setup. i) Real‐time change in the optical power with varying glucose concentration. Reproduced with permission.^[^ ^79^ ^]^ Copyright 2018, American Chemical Society.

Consequently, the voltage drops at the gate/electrolyte interface, increasing the potential applied to the active channel and decreasing the drain current. Although electrochemical sensors provide accurate and selective detection of analytes, the presence of metal electrodes could introduce discomfort for the user if the sensors are used for an extended period. Kikuchi et al. demonstrated one of the early glucose‐sensing devices using a platinum electrode fabricated with a gel membrane comprising polyvinyl alcohol and a phenylboronic acid (PBA) containing polymer.^[^ ^77^ ^]^ Lee et al. utilized a similar concept to fabricate inverse opal hydrogels for glucose sensing.^[^ ^78^ ^]^ To achieve this, 2‐hydroxyethyl methacrylate and 3‐acrylamidophenylboronic acid were photopolymerized within the interstitial space of a polystyrene colloidal template. PBA binds reversibly with glucose and alters the volume of the hydrogel matrix. This volumetric change was detected as a shift in the Bragg's peak. Recently, Elsherif et al. fabricated a wearable contact lens optical sensor to quantify glucose that allowed smartphone readouts (**Figure** **5**).^[^ ^79^ ^]^ The sensor design included creating a periodic structure of hydrogel containing PBA by drop‐casting on a photonic structure stamp followed by UV curing (Figure 5a). The result was a 1D photonic structure of a sensing film with a periodicity of 1.6 μm. Due to changes in the glucose concentration, the periodic constant of the microstructures is altered, resulting in a change in the diffraction angle of the transmitted light (Figure 5b–e). A sensitivity of 12 nm mM^−1^, a response time of 3 s, and a saturation time of 4 min for continuous monitoring were achieved (Figure 5i). Park et al. developed a calorimetric contact lens for noninvasive glucose monitoring in human tears.^[^ ^80^ ^]^ The sensor included glucose oxidase conjugated with cerium oxide NPs using polyethylene glycol. In the presence of glucose oxidase, glucose is oxidized, producing H_2_O_2_, which further converts Ce^3+^ to Ce^4+^, changing the color of the contact lens from an initial pale yellow to a deep yellow color. This color change can be detected using a mobile phone and correlated to the concentration of glucose in the tear fluid. The contact lens was fabricated by mixing and photopolymerizing a mixture of hydroxyethyl methacrylate and glucose‐oxidase‐conjugated cerium oxide NPs. The nontoxicity of the contact lens was tested using human corneal and endothelial cells. Glucose levels were measured by placing the lens in the eyes of diabetic rabbits. Contact‐lens‐based glucose sensors, which operate electrochemically, have several limitations, including the production of AgCl at the electrode interface, discomfort due to metal contact, and protein aggregation on the substrate. Colorimetric sensors, in contrast, have delayed kinetics, poor reproducibility, and reduced transparency due to coating (see Table S1, Supporting Information, for a detailed comparison of all reported approaches).

**Figure 6:**
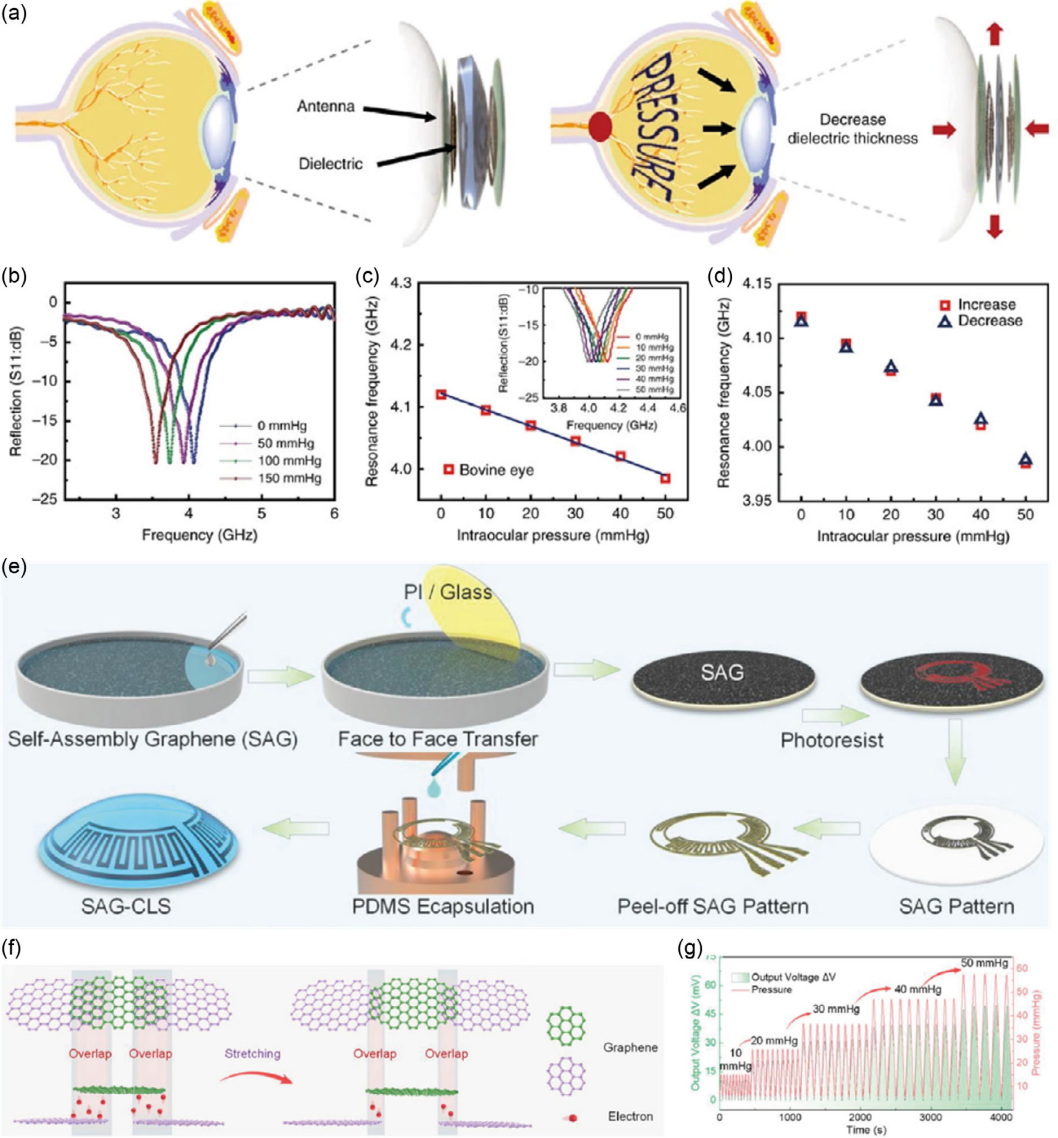
Intraocular pressure sensors. a) Schematic illustration showing the mechanism of intraocular pressure sensing using sensor‐integrated contact lens. b) Reflection coefficients at different pressures were wirelessly collected from the contact lens sensor worn by the bovine eyeball. c) Frequency response of the intraocular pressure sensor on the bovine eye (inset: the corresponding reflection coefficients of the sensor). d) Frequency response of the sensor during a pressure cycle. (a–d) Reproduced under the terms of the Creative Commons Attribution 4.0 International License.^[^ ^73^ ^]^ Copyright 2017. The author(s), Published by Springer Nature. e) Schematics of graphene‐enhanced contact lens sensor fabrication. f) The sensing mechanism of self‐assembled graphene‐enhanced contact lens. g) Change in the output voltage with pressure tested using a bionic eyeball test platform. (e–g) Reproduced with permission.^[^ ^84^ ^]^ Copyright 2021, Wiley‐VCH GmbH.

Glaucoma is an irreversible chronic eye disease that can lead to vision loss in a few cases and requires continuous medical care for the patient's lifetime.^[^ ^81^, ^82^ ^]^ In glaucoma, there is damage to the drainage angle of the eye, and thus fluids are not drained away. This results in an elevated intraocular pressure (IOP). Therefore, IOP is an important indicator of glaucoma. Traditionally, IOP is measured using a Goldmann applanation tonometer and requires visits to a clinic. Recently, the iCare HOME tonometer has been demonstrated for measuring IOPs at home. Several wearable tonometers have been reported to be capable of continuous and long‐term monitoring of IOP. However, these wearable tonometers can cause eye pain, superficial punctate keratitis, corneal epithelial defects and conjunctival erythema.^[^ ^83^ ^]^ Kim et al. utilized a graphene–AgNW hybrid electrode for in vitro IOP measurement using a bovine eyeball (**Figure** **6**a–d).^[^ ^73^ ^]^ The IOP values obtained using this smart contact lens correlated well with the real pressure measured using a pressure sensor previously inserted into the eyeball. Recently, Liu et al. fabricated a “fish scale”‐like film by self‐assembling graphene and integrated this microstructure into a contact lens for monitoring IOP (Figure 6e–g).^[^ ^84^ ^]^ A face‐to‐face wafer transfer method was used to transfer the self‐assembled graphene film onto the PI, creating Wheatstone strain gauge‐circuit electrodes. This assembly was finally transferred to a flexible PDMS film shaped as a contact lens and used to measure IOP. The Wheatstone bridge strain gauge circuit was supplied with a constant DC potential and the output voltage was measured—the output voltage depends on the pressure change. The sensor was transparent and had a sensitivity of 1.0164 mV mmHg^−1^ on a silicone eye and 3.166 mV mmHg^−1^ in vitro on a porcine eye. The sensor had high stability even after 30 cycles. Zhang et al. fabricated a contact lens tonometer using a chemical vapor deposition‐grown graphene‐woven fabric to monitor IOP with high resolution.^[^ ^85^ ^]^ Under constant voltage, as the pupil dilates, IOP increases. This increase in IOP will stretch the contact lens and consequently, the graphene‐woven fabric will increase its electrical resistance. In contrast, a recovery of IOP leads to a decrease in the resistance of graphene fabric. The change in current was noted with an average resolution of 6.8% mmHg^−1^ in the range of 0–10 mmHg. The main challenges with contact lens tonometers are that they are bulky and rigid, which often block the user's vision. Kim et al. recently reported a flexible, soft, and transparent contact lens tonometer.^[^ ^86^ ^]^ The lens served as a strain sensor with a performance similar to a commercial tonometer commonly used in rabbits and humans. The contact lens had a mechanically reinforced ring pattern that was disconnected near the rim region.

**Figure 7:**
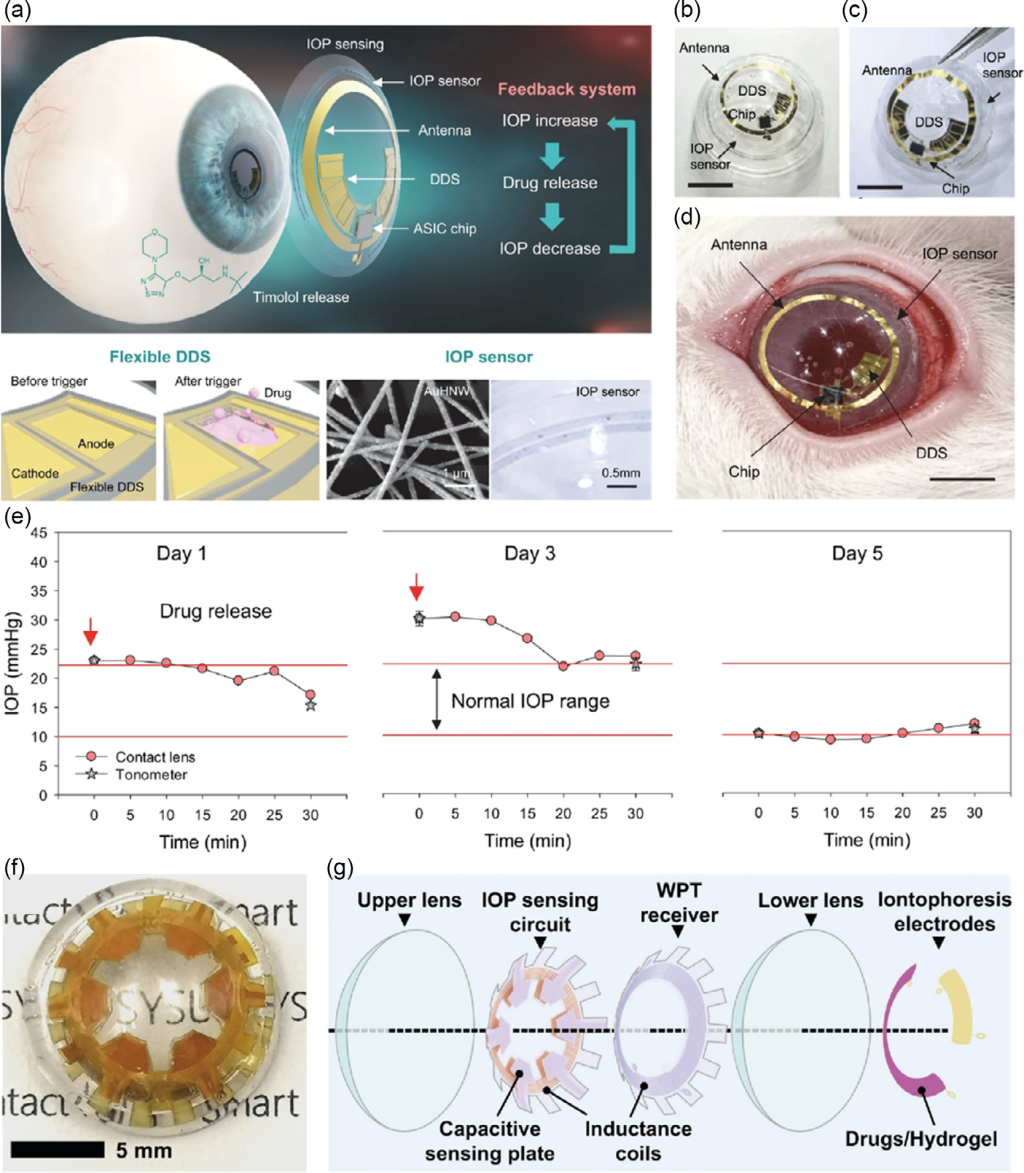
a) Schematic of the structure of a theranostic smart contact lens with a fully integrated gold hollow nanowire‐based IOP sensor, a drug delivery system, and a wireless circuit for wireless glaucoma treatments with a feedback system for IOP sensing and timolol release. b,c) Optical images of daily and weekly types of theranostic smart contact lenses (scale bar: 5.5 mm). d) Photograph of a fully integrated theranostic smart contact on rabbit's eye (scale bar, 5.5 mm). e) The IOP control of the theranostic smart contact lens by simultaneous IOP monitoring and timolol release (*n* = 5 independent experiments for tonometer except for day 1 (*n* = 3). (a–e) Reproduced under the terms of the Creative Commons Attribution 4.0 International License.^[^ ^33^ ^]^ Copyright 2022. The author(s), Published by Springer Nature. f) Photograph of a wireless theranostic contact lens. g) Schematic illustration of various components and functions of wireless theranostic contact lens. (f,g) Reproduced under the terms of the Creative Commons Attribution 4.0 International License.^[^ ^87^ ^]^ Copyright 2022, The author(s). Published by Springer Nature.

A strain sensor is placed between the reinforced and soft elastic region of the ring pattern to detect very small changes in IOP with a sensitivity of 0.05% mm^−1^ Hg^−1^. Integrating contact lenses with sensing and drug delivery systems will offer theranostic systems for real‐time regulation of glaucoma. However, due to the small size, flexibility, and ultrathin nature of contact lenses, integrating theranostic systems is expected to be a significant challenge. Kim et al. developed a theranostic contact lens for IOP monitoring and drug administration (**Figure** **7**a).^[^ ^33^ ^]^ This contact lens serves as a substrate for a pressure sensor made of hollow Au wires, a flexible drug delivery system, wireless circuitry, and an application‐specific integrated circuit chip **(**Figure 7b,c). The contact lens could deliver timolol on demand for IOP control. The fully integrated theranostic contact lens was tested on a glaucoma‐induced rabbit eye (Figure 7d). The controlled release of timolol reduced 22% of glaucoma, and the values measured for IOP were identical to those obtained using a tonometer (Figure 7e). In another approach, Yang et al. reported a wireless theranostic contact lens (Figure 7f).^[^ ^87^ ^]^ The design included an IOP‐sensing circuit and a wireless power transfer receiver sandwiched between the upper and lower lenses. The lower lens is fabricated with antiglaucoma drug‐coated hydrogel and electrodes, allowing the triggering of the drug administration via iontophoresis (Figure 7g).

MMP‐9 in teardrops is one of the biomarkers for detecting dry eye disease.^[^ ^88^ ^]^ Ye et al. fabricated a flexible contact lens sensor to monitor IOP and MMP‐9.^[^ ^89^ ^]^ The lens comprises anti‐opal photonic crystal structures built using silica NP templates, which diffract light according to changes in the lattice spacing. These changes in the lattice spacing are due to variations in the IOP. MMP‐9 sensing was performed using surface‐enhanced Raman spectroscopy. Au nanobowls coated with a fluorescent Raman tag peptide were used and the intensity of the Raman peak was correlated with the concentration of MMP‐9. An electroretinogram is an ophthalmic examination to diagnose many ocular diseases, including glaucoma, retinitis pigmentosa, diabetic retinopathy, retinoschisis, and many other congenital degradations.^[^ ^90^, ^91^, ^92^ ^]^

One of the major hindrances that patients face during their regular checkups is physical discomfort due to the use of rigid sensors. This situation may require the use of anesthesia and pain medication. To address this issue, Kim et al. fabricated a corneal sensor that can be integrated inside a soft contact lens to obtain electroretinograms in a seamless manner.^[^ ^93^ ^]^ They achieved this painless outcome by placing a circular serpentine trace of the conducting path near the edge of the contact lens, which was connected to an external port for data acquisition. This conducting path consists of a composite polystyrene‐b‐poly(ethyl‐co‐butane)‐*b*‐polystyrene and is encapsulated using fumed silica‐NP‐filled PDMS. Using this contact lens, the electroretinogram was recorded following the International Society for Clinical Electrophysiology of Vision guidelines and protocols. The reported IOP sensors have laborious fabrication steps that are cost‐intensive. Furthermore, long‐term performance evaluation is needed to understand the accuracy of the sensors (see Table S2, Supporting Information, for a summary of different techniques for measuring IOP and their limitations).

In addition to glucose, lactate is an important metabolite in tear fluids. Elevated lactate levels could indicate oxygen deficiency, ischemia, sepsis, liver disease, and even cancer.^[^ ^94^, ^95^ ^]^ Thomas et al. used contact lenses as a flexible substrate to demonstrate amperometric, mono‐enzymatic l‐lactate sensing.^[^ ^96^ ^]^ The sensor was fabricated using a biocompatible polyurethane substrate coated with Ti/Pd/Pt as the working, reference, and counter electrodes. The electrodes were further functionalized with the lactate oxidase enzyme. Lactate oxidase reduces lactate to pyruvate and produces H_2_O_2_, which was detected with an average sensitivity of ≈53 μA mM^−1^. Reid et al. fabricated a biofuel cell on a contact lens for detecting lactate in a teardrop.^[^ ^97^ ^]^ They used modified buckypaper and silicone elastomers for the biofuel cell design to generate a power density of 8.1 ± 1.4 μW cm^−2^. However, the biofuel cell was unable to differentiate between glucose and lactate. One of the major limitations of contact‐lens‐based wearable sensors for continuous tear biomarker monitoring is the poor flexibility, conformability, and unavailability of soft electrodes. The connections to transfer the signal from the electrodes to a device also require further experimentation. A summary of various lactate sensors and their advantages and limitations is presented in Table S3, Supporting Information.

## 5 Oral and Nasal Breath Metabolite Sensing

**Figure 8:**
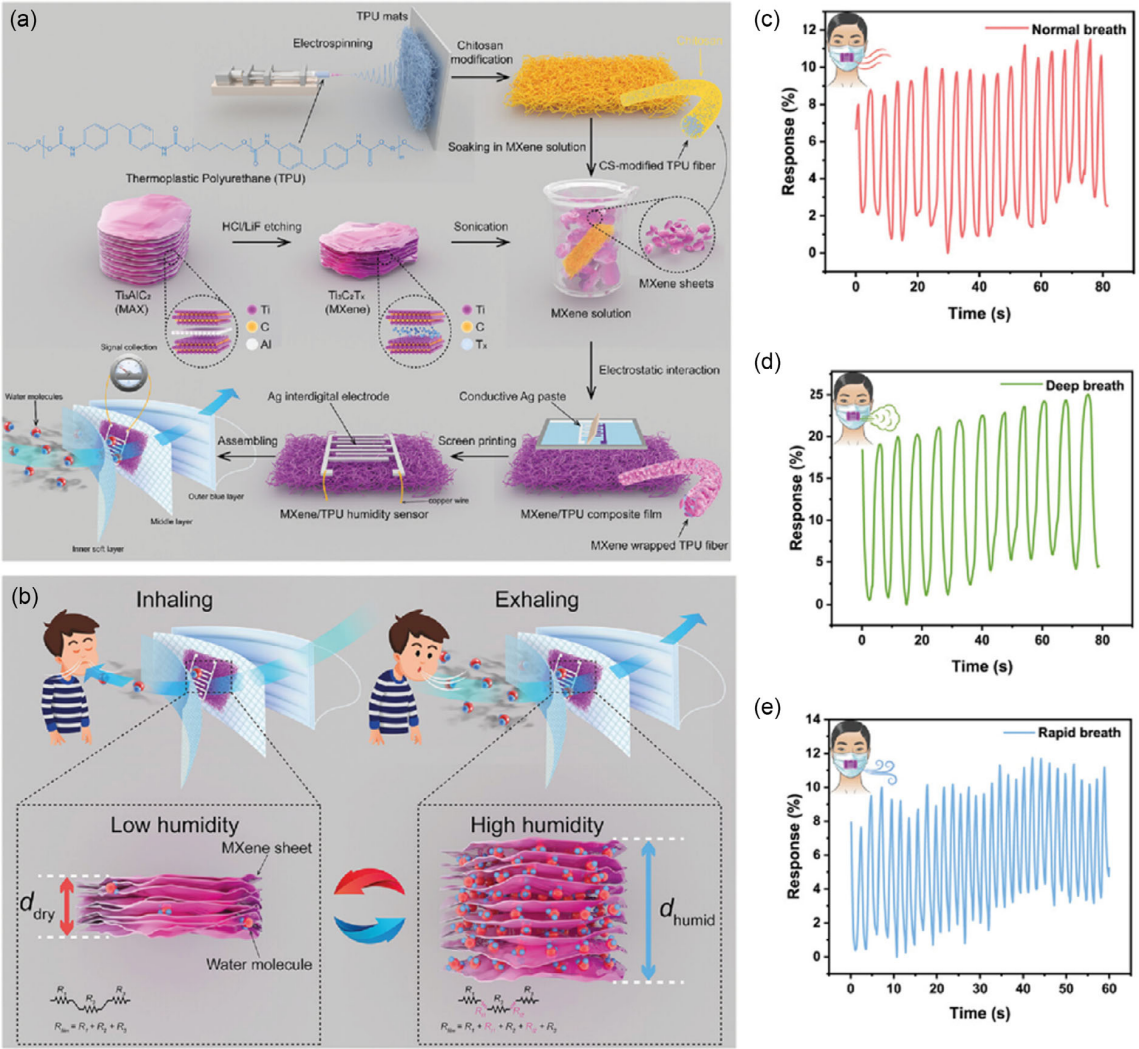
Smart mask for sensing breath metabolites. a) Schematic illustration showing various components and fabrication of MXene/thermoplastic polyurethane sensor. b) Schematic image describing the humidity‐sensing mechanism of the MXene/TPU sensor. R1, R2, and R3 represent the intrinsic resistance of MXene nanosheets. Rt1 and Rt2 represent tunneling resistance at the junctions. Rfilm represents the total resistance of MXene/TPU composite film. c–e) Respiration response curves from normal, deep, and rapid breathing. Reproduced under the terms of the Creative Commons Attribution 4.0 International License.^[^ ^111^ ^]^ Copyright 2023, The Author(s). Advanced Sensor Research published by Wiley‐VCH GmbH.

Exhaled breath condensate contains nearly 3500 biomarkers (i.e., metabolites), which could help understand the physiological status of an individual. Real‐time noninvasive monitoring and analysis of breath metabolites could pave the way for breakthroughs in medical technology as they could help in the early detection of diseases and create personalized medical treatments. The COVID‐19 pandemic has shown that wearing a face mask can be one passive measure to prevent virus transmission by up to 85%, depending on the type of mask used.^[^ ^98^ ^]^ Conventionally, face masks protect the individual from ambient air pollution indoors and outdoors.^[^ ^99^ ^]^ Air pollution is a global phenomenon that has resulted in approximately 7 million deaths annually.^[^ ^100^ ^]^ According to the World Health Organization (WHO), these deaths can be prevented by wearing face masks.^[^ ^101^, ^102^ ^]^ However, currently, face masks are primarily used to prevent exposure to external pollutants and microbial transmission. These same masks can be potentially utilized to collect breath analytes. Among the constituents of exhaled breath, humidity and CO_2_ concentration are analytes of interest. Recent studies indicate that humidity can be related to different respiratory conditions such as asthma, chronic obstructive pulmonary disease (COPD), and even cystic fibrosis.^[^ ^103^, ^104^ ^]^ Sensing of exhaled breath humidity has been achieved using conducting polymers, metal oxide NPs, metal organic frameworks,^[^ ^105^, ^106^, ^107^ ^]^ 2D transition metal carbides and nitrides (MXenes), graphene, and other carbon‐based materials.^[^ ^108^, ^109^, ^110^ ^]^ Liu et al. reported a humidity sensor with a response time of 12 s operating in the 11–94% relative humidity (RH) capable of differentiating breath rates (**Figure** **8**).^[^ ^111^ ^]^ Briefly, the sensor comprised of electrospun thermoplastic polyurethane (TPU) nanofiber mat modified with chitosan. The mat was further modified with MXene coating and screen‐printed silver electrodes (Figure 8b). The sensor‐integrated face mask was able to distinguish normal, rapid, and deep breath during activities such as running, walking, and jogging (Figure 8c–e). Recently, we reported electrospun nanofibers of PVDF rGO and polypropylene (PP) fabric as substrates for in situ polymerization of polyaniline using successive ionic layer adsorption reactions.

**Figure 9:**
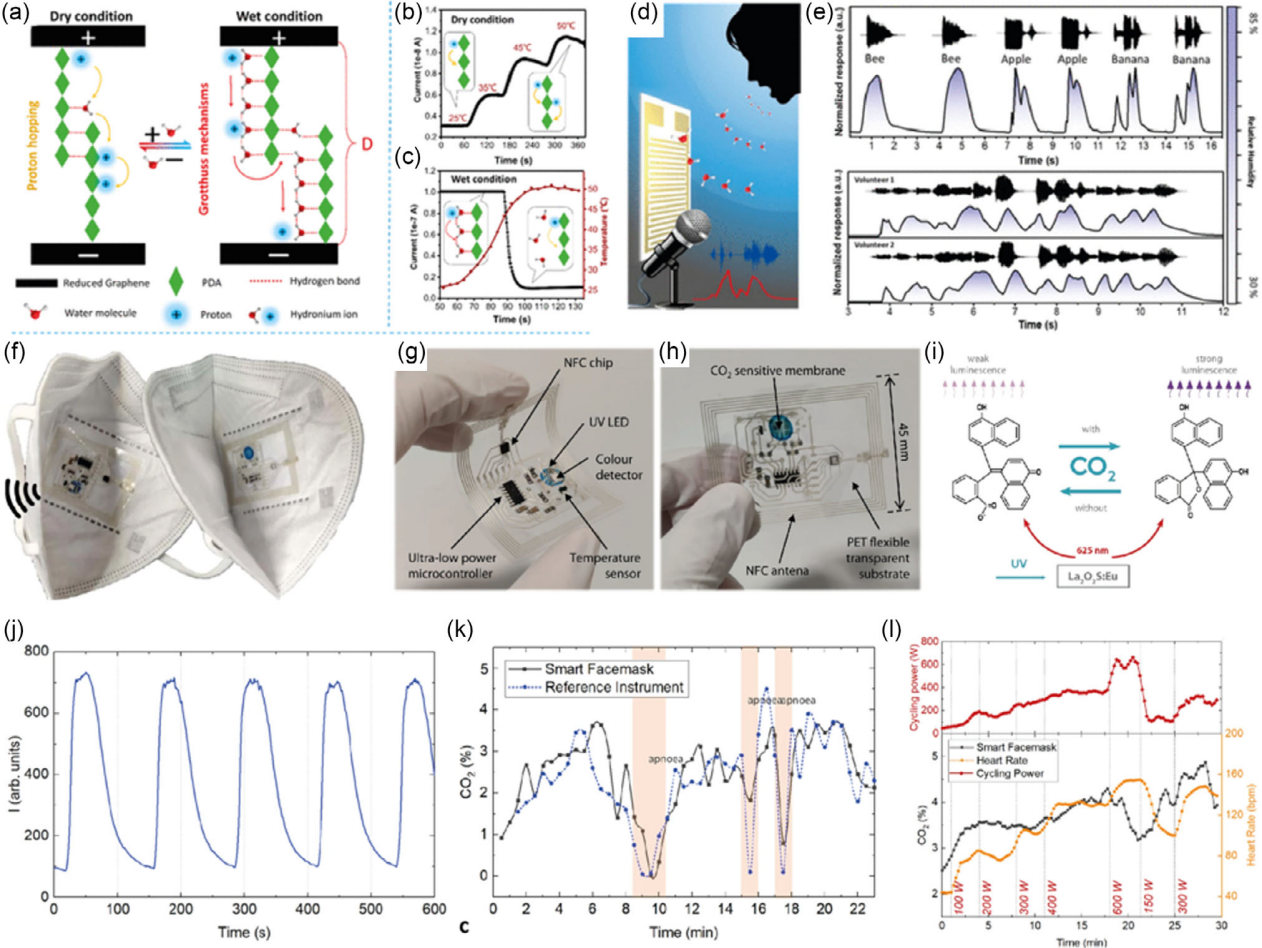
a) Sensing mechanism showing possible proton transport mechanisms in graphene–polydopamine hybrid materials under dry and wet conditions. b) The increase in conductivity as temperature increases suggests proton hopping. c) Under a wet atmosphere, the conductivity decreases sharply to near [H_2_O] ≈8858 ppm. d) Schematic illustration of a humidity sensor for human exhaled air detection during speaking. e) Repeated responses of a graphene nanochannels confined poly(dopamine) sensor to three different words and the song “Twinkle Twinkle Little Star” sung by two volunteers. (a–e) Reproduced with permission.^[^ ^115^ ^]^ Copyright 2018, American Chemical Society. f) FFP2 face mask integrated with a CO_2_‐sensitive membrane deposited on a flexible NFC tag. g,h) Photographs of the 125 μm thick PET substrate with printed flexible NFC tag. i) CO_2_‐sensing mechanism. j) Response and recovery times of the developed sensor while increasing and decreasing CO_2_ concentrations between 0% and 100%. k) Smart face mask performance during short‐term use. l) Smart mask performance during graded cycling exercise tests. (f–l) Reproduced under the terms of the Creative Commons Attribution 4.0 International License.^[^ ^119^ ^]^ Copyright 2022, The author(s). Published by Springer Nature.

The resulting substrate was used to sense humidity in the exhaled breath from the nose and mouth, with a rapid response and recovery times.^[^ ^112^, ^113^ ^]^ The humidity switching was synchronous with the breath rate. Therefore, it allowed the estimation of breathing rate and the ability to differentiate nasal and oral exhalations. Furthermore, it also differentiated rapid, normal, and slow breathing rates for different individuals. Yao et al. fabricated a sensor using GO drop‐casted on a laser‐induced graphitized PI substrate for detecting humidity in exhaled breath with a response and recovery time of 2 and 35 s.^[^ ^114^ ^]^ This sensor was robust enough to differentiate between different breathing rates, coughing, and breathing rates of a volunteer suffering from rhinitis. He et al. reported a high‐performance humidity sensor containing a graphene–polydopamine heterogeneous‐sensing junction (**Figure** **9**a).^[^ ^115^ ^]^ The composite allowed for a highly precise interlayer spacing varying between 0.7 and 1.4 nm (Figure 9b,c). Graphene oxide flakes were functionalized using polydopamine and drop‐casted on a flexible polyethylene terephthalate (PET) substrate. The resulting substrate was used to detect humidity in exhaled breath operating under the RH range of 0–97% with response and recovery times of 20 and 17 ms. Volunteers could speak repeatedly, and humidity fluctuations were used for voiceprint recognition (Figure 9d,e). This sensor was placed inside a nebulizer mask to monitor the breathing rate while jogging. Mogera et al. recently reported a humidity sensor based on a supramolecular assembly of coronene tetracarboxylate and dodecyl methyl viologen with an unprecedented response and recovery times of 10 ms.^[^ ^116^ ^]^ In addition to measuring the humidity in the exhaled breath, this sensor can be used as a diagnostic tool to study the %RH before and after exercise, and after consumption of alcohol. The authors observed that humidity decreased immediately after exercise and ≈4 h later, the humidity in the exhaled breath reached its normal values. Furthermore, the sensor was able to detect that immediately after consumption of alcohol, the humidity in the exhaled breath reduced drastically. Such a sensor can provide important insights into the understanding of hydration mechanisms. A flexible breath humidity sensor was recently reported wherein cellulose nanofibers, along with CNTs were printed on a flexible PET substrate via a near‐field electrodynamic direct‐writing method. This new sensor was used to monitor humidity in the exhaled breath while breathing through the nose, mouth, holding, yawning, and coughing.^[^ ^117^ ^]^ Even though the sensor was robust to capture the quick spikes in the resistance while speaking words, it was not yet able to discern the words. Despite growing interest in wearable humidity sensors, they suffer from complex fabrication processes, high costs, limited air breathability, and extended recovery times (see Table S4, Supporting Information, for a detailed summary of various humidity sensors, their advantages, limitation, and performances).

Carbon dioxide (CO_2_) is another important biomarker and the abnormally high concentration of CO_2_ in the blood stream results in hypercapnia.^[^ ^118^ ^]^ Chronic obstructive pulmonary disease (COPD), a respiratory disease, is known to increase the concentrations of CO_2_ in the blood. Commonly, blood is used to measure the concentration of CO_2_. In theory, by measuring the concentration of CO_2_ in the exhaled breath, one could noninvasively link the obtained value to the concentration of CO_2_ in the blood. Escobedo et al. incorporated a CO_2_ sensor inside a face mask to monitor the concentration of CO_2_ inside the mask and transmit the data wirelessly using near‐field communication (NFC) protocol (Figure 9f–h).^[^ ^119^ ^]^ Eu‐doped La_2_O_2_S nanocrystals acted as a luminophore and α‐naphtholphthalein, a pH indicator was used as an acceptor for La_2_O_2_S:Eu luminescence (Figure 9i). In the presence of CO_2_, the pH indicator is protonated, reducing the phosphor attenuation and increasing the sensor's luminescence. A UV light emitting diode (LED) was used to illuminate the CO_2_‐sensitive membrane and changes in intensities in the presence of CO_2_ were monitored using a digital color sensor. The sensor was exposed to concentrations of CO_2_ from 0% to 100% and exhibited a rapid response time of 0.33 ms (Figure 9j–l). The shelf‐life of the sensor in ambient conditions was approximately 140 days. Furthermore, the shelf‐life could be further extended by storing the sensor in an inert atmosphere. Recently, a sensor for sensing breath CO_2_ was fabricated by coating the tip of an optical fiber using an organically modified silica film embedded with a pH‐sensitive dye, thymol blue, and tetramethylammonium hydroxide.^[^ ^120^ ^]^ In reflection mode, the sensor can detect CO_2_ in the exhaled breath within a ≈33% error compared to the values obtained from a commercial CO_2_ datalogger. However, the response of the sensor degraded after 10 days. The major limitations of optical CO_2_ sensors inside face masks are that they are slow in response, not affordable, and require microheating, which could be uncomfortable for the user. The colorimetric CO_2_ sensors despite allowing real‐time sensing, suffer from potential photobleaching upon prolonged illumination (see Table S5, Supporting Information, for a more detailed description of each sensor).

**Figure 10:**
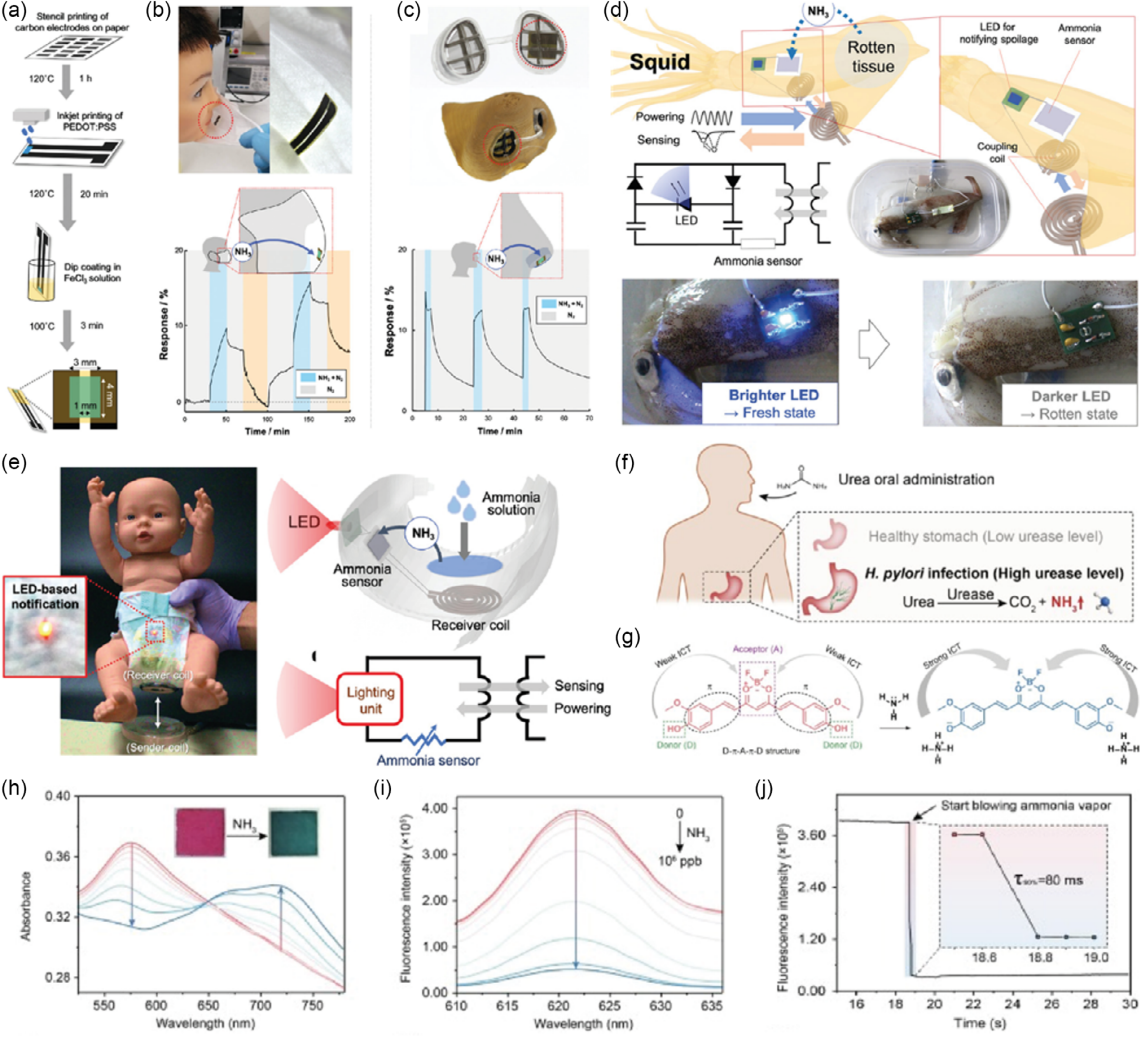
a) Schematics of the fabrication of a paper‐based ammonia sensor. b) Photograph of paper‐based sensor integrated into a nonwoven face mask and the sensor performance by measuring its response to ammonia (blue regions), recovery using nitrogen at 25 °C (gray regions) and recovery by heating at 37 °C in a nitrogen environment (orange regions). c) Photograph of the sensor integrated in a plastic nasal filter and its response to ammonia (blue regions) and recovery in nitrogen (at 25 °C, gray regions). d) Wireless ammonia gas sensing from a spoiling squid using the light intensity of a wirelessly powered LED showing the schematics and photographs of fresh‐ and rotten‐state sensing. e) LED‐based wireless ammonia sensing using a paper‐based sensor embedded in a diaper. (a–e) Reproduced with permission.^[^ ^136^ ^]^ Copyright 2022, Wiley‐VCH GmbH. f) Graphical illustration of breath ammonia increases in *Helicobacter pylori* infection. g) Chemical structures and ammonia‐sensing mechanism in curcumin derivatives. h) UV spectra of nanofibers in different concentrations of ammonia vapor (0–10^6^ ppb). i) Emission spectra of the nanofibers under excitation at 576 nm in different concentrations of ammonia vapor (0–10^6^ ppb). j) Fluorescence quenching of the curcumin dye upon blowing over with ammonia vapor (1000 ppm). (f–j) Reproduced with permission.^[^ ^138^ ^]^ Copyright 2022, Elsevier B.V.

Ammonia is an important biomarker as it is the byproduct of the breakdown of proteins. Ammonia is converted into urea in the presence of CO_2_ via the urea cycle, which enters the bloodstream and is excreted in urine.^[^ ^121^ ^]^ Blood ammonia concentrations are typically in the 11–50 μM range for a healthy individual. Concentrations higher than 100 μM affect multiple organs and can result in cognitive and neurological impairments.^[^ ^122^, ^123^, ^124^, ^125^ ^]^ Noninvasive measurement of ammonia in exhaled breath could become a primary diagnostic tool to understand the functioning of the liver, kidney, and drug metabolism.^[^ ^126^ ^]^ Conducting polymers and composite materials such as polyaniline (PANi),^[^ ^127^, ^128^, ^129^, ^130^ ^]^ polypyrrole (PPy),^[^ ^131^, ^132^, ^133^ ^]^ PEDOT:PSS, and CNTs^[^ ^134^, ^135^ ^]^ have been extensively studied for their suitability in sensing ammonia. Fujita et al. recently reported a humidity‐resistant and humidity‐sensitive paper‐based breath ammonia sensor (**Figure** **10**).^[^ ^136^ ^]^ Their design included a stencil printing of carbon electrodes on a paper substrate, followed by inkjet printing of PEDOT:PSS‐interdigitated electrodes and treatment with FeCl_3_ (Figure 10a).

The sensor was integrated inside a face mask and a nasal filter to measure the breath ammonia concentrations (Figure 10b,c). The response of the sensor was measured by exposing it to 0.2 L min^−1^ of simulated breath containing ammonia. Furthermore, a battery‐free wireless alert system was developed to detect food spoilage by integrating the ammonia sensor into a resonant circuit with LEDs. Upon increased ammonia levels, the LED loses power allowing the detection of rotten meat. This approach was also applied in the incorporation of the sensor inside a diaper to detect ammonia (Figure 10e). Wu et al. recently presented a polypropylene‐fabric‐based breath ammonia sensor.^[^ ^137^ ^]^ The polypropylene fabric was coated with multiwalled carbon nanotubes and PANi via in situ polymerization, followed by silver painting. When embedded inside a face mask, the sensor detected a maximum of 70 ppb of ammonia in breath and provided information on the respiration rate. The authors observed a response and recovery time of ≈93 and 96 s while dynamically switching the concentration of the ammonia gas. Song et al. fabricated an ultrafast, sensitive fluorescent breath ammonia sensor based on curcumin derivatives (Figure 10f–j).^[^ ^138^ ^]^ Briefly, a boron‐trifluoride‐treated curcumin dye was loaded with polyvinyl pyrrolidone to fabricate electrospun fluorescent nanofibers. Upon interacting with ammonia molecules, the internal charge transfer in the dye molecules is affected (Figure 10g). The electrospun fiber displays a strong absorption peak at 576 nm and no absorption peak centered at 715 nm in their UV–vis spectra (Figure 10h). In the presence of ammonia, the absorption peak at 576 nm gradually decreased and a new peak emerged at 715 nm (Figure 10h). The sensor's limit of detection (LOD) was measured at 22 ppb, and it could even sense 10^4^ ppb of ammonia with a response of ≈80 ms. This sensor was used to measure the ammonia concentration in the exhaled breath of patients with chronic kidney disease pre‐ and post‐hemodialysis. The results indicate a significant reduction in breath ammonia post‐dialysis.

**Figure 11:**
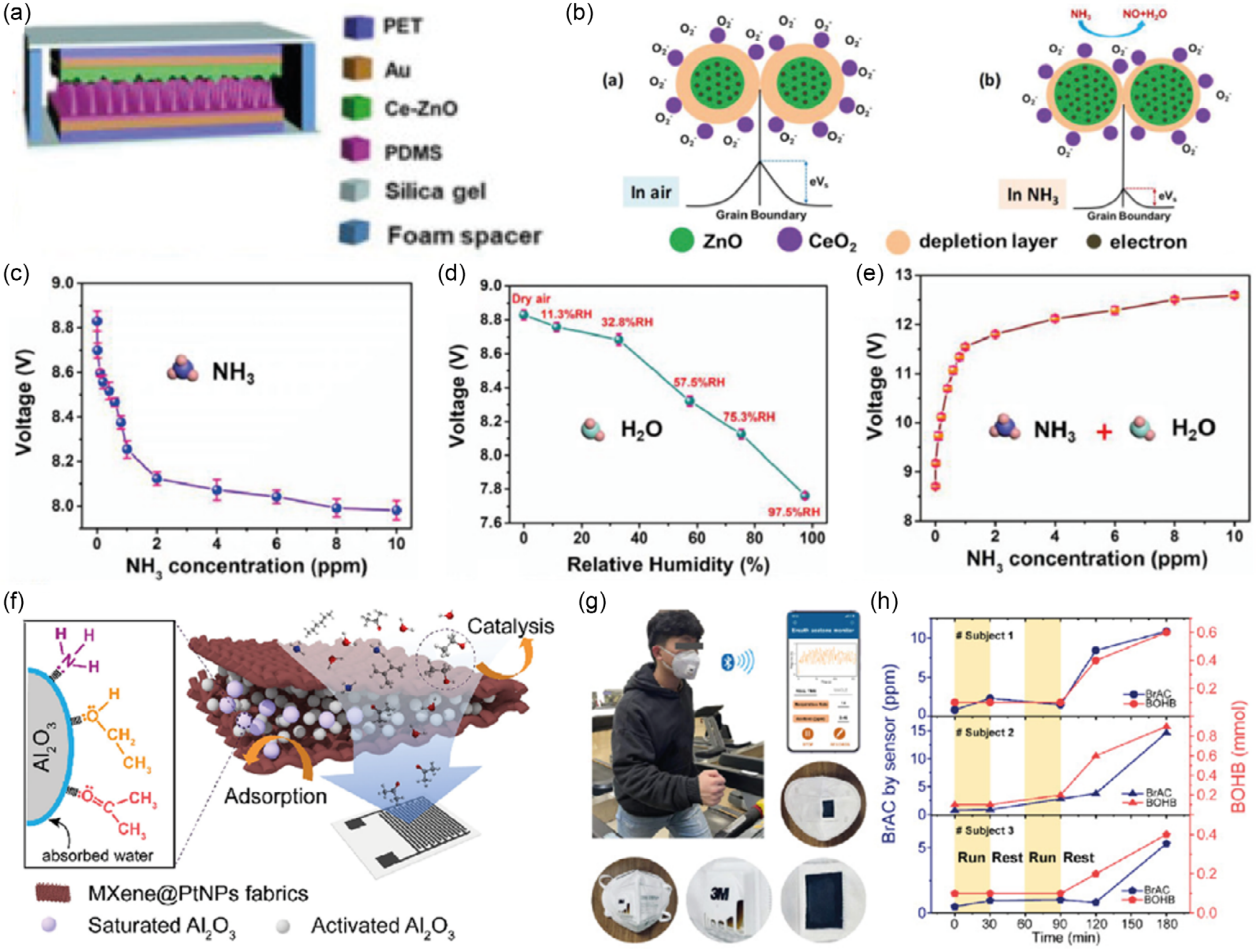
a) Schematics of a TSRS and its components. b) The gas‐sensing mechanism of Ce‐doped ZnO is in the air (left) and ammonia (right). c) Voltage v/s ammonia concentration for a Ce–ZnO film under dry conditions. d) Voltage as a function of increasing RH for a Ce–ZnO film. e) Voltage as a function of increasing ammonia concentration in the presence of 97.5% RH for a Ce–ZnO film. (a–e) Reproduced with permission.^[^ ^141^ ^]^ Copyright 2022, Elsevier B.V. f) Schematic illustration of the working principle of the MXene@Pt NPs‐functionalized textile filter. g) Photographs of a volunteer wearing a sensor‐integrated face mask during exercise and the images of the detection tag and textile filter amounted in the face mask. h) The breath acetone (BrAc) level and *β*‐hydroxybutyrate (BOHB) references during and post alternating aerobic–anaerobic running. Reproduced with permission.^[^ ^150^ ^]^ Copyright 2023, Elsevier B.V.

The sensing of ammonia using metal‐oxide‐based sensors has been well documented in the literature.^[^ ^139^, ^140^ ^]^ However, one of the major drawbacks of using metal‐oxide‐based sensors is their higher operational temperature. Wang et al. designed a triboelectric self‐powered wearable respiration sensor (TSRS) (**Figure** **11**a).^[^ ^141^ ^]^ The sensor comprised a PET substrate on which Au electrodes were coated. The flexible substrate was spray coated with Ce‐doped ZnO NPs on one electrode. The top layer consisted of Au‐coated PET with patterned PDMS forming TSRS. The sensor could calculate the breath rate and simultaneously measure concentrations of ammonia between 0.1 and 10 ppm under 97% RH (Figure 11c–e). More importantly, the results indicated that the output voltage of the TSRS was inversely proportional to the concentration of ammonia when the sensor was exposed to dry ammonia (Figure 11c). Similarly, increasing the RH (11.3–97.5% RH) decreased the output voltage (Figure 11d). Notably, in the presence of high humidity, the selectivity and sensitivity of ammonia were enhanced with a rapid increase in output voltage as the ammonia concentration increased (Figure 11e). Wang et al. designed a respiration‐driven triboelectric nanogenerator (TNEG) for measuring breathing rate and detecting trace concentrations of ammonia.^[^ ^142^ ^]^ The exhaled breath was collected inside an expanding balloon in which the TNEG, made of two flexible PET substrates, was located. One of the PET substrates was coated with Au with patterned PDMS and the other was Ce‐doped ZnO PANi. This TENG sensor was able to detect ammonia concentrations ranging from 0.1 to 1 ppm in addition to determining breath rate recognition. The major limitations of ammonia sensors are their poor response and recovery time, and narrow sensing range. Furthermore, the sensor fabrication is laborious, and they operate at elevated temperatures (see Table S6, Supporting Information, for a detailed summary comparing various ammonia sensors).

Acetone is another important biomarker released during ketosis, a metabolic process occurring in hepatic mitochondria after *β*‐oxidation of fatty acids.^[^ ^143^ ^]^ Acetone concentration can be correlated to the physiological status of an individual during fasting, exercise, or experiencing diabetes, obesity, and pregnancy.^[^ ^144^, ^145^, ^146^, ^147^, ^148^ ^]^ Real‐time monitoring of acetone concentration in breath can be achieved using wearable sensors.^[^ ^149^ ^]^ Li et al. reported an acetone sensor that can be incorporated inside a face mask and wirelessly transfer data using a low‐energy Bluetooth module (Figure 11f–h).^[^ ^150^ ^]^ To detect acetone inside breath, in situ grown TiO_2_ and short peptides were incorporated on a MXene substrate. The integration of an MXene sensor to a face mask comprised a textile filter, a PDMS‐encapsulated LED, and electronic circuitry. The acetone in breath (≈5 ppm) and acetone in blood were measured during and post‐gym exercise from three volunteers (Figure 11g,h). This sensor also allowed the monitoring of acetone in the breath of subjects consuming a balanced diet, a ketogenic diet, and a carbohydrate diet. Dai et al. reported a self‐powered acetone sensor that was incorporated inside a face mask.^[^ ^151^ ^]^ The sensor comprised a PVDF film, taped on both sides with Kapton tape and placed between two silver electrodes. Due to the piezoelectric nature of PVDF film, a voltage of ≈2 V was generated while breathing. This voltage was utilized to power the acetone sensor made of sodium‐doped ZnO nanoflowers drop‐casted on a flexible PET substrate with Au‐interdigitated electrodes. The sensor was able to detect a maximum acetone concentration of 7 ppm, even after 7 days of use, with a response time of 20 s. The acetone sensors reported in the literature suffer from slow response and recovery time and require laborious fabrication steps (see Table S7, Supporting Information, for the comparison of performance of different acetone sensors).

Macrophages and neutrophils produce hydrogen peroxide (H_2_O_2_) in response to respiratory diseases. Therefore, H_2_O_2_ in exhaled breath is an important biomarker to monitor respiratory illness.^[^ ^152^, ^153^ ^]^ Recently, Maier et al. reported a paper‐based electrochemical H_2_O_2_ sensor by printing wax on paper to create a hydrophobic barrier.^[^ ^154^ ^]^ The sensor included Prussian‐blue‐mediated carbon and blank carbon electrodes for H_2_O_2_ detection and background signal subtraction. The electrochemical cell comprised an Ag/AgCl reference electrode and a counter carbon electrode. The Prussian blue at the sensing electrode oxidizes upon interacting with H_2_O_2_. The sensor was able to detect H_2_O_2_ at approximately 350 μM, and the output current density was linearly dependent on the concentration of H_2_O_2_. Cao et al. demonstrated a sensor placed inside a face mask for electrochemical sensing of H_2_O_2_ in exhaled breath condensate.^[^ ^155^ ^]^ A 3D PANi structure was electrochemically grown over a glass electrode, which was further treated with chitosan, acetic acid, and a sacrificial porous alumina template. Personnel driving in an inebriated state is not only a risk for themselves but also can cause harm to bystanders and passengers traveling in other cars.^[^ ^156^, ^157^ ^]^ Electrochemical sensors could be powered using TNEGs for continuous breath H_2_O_2_ sensing without needing external power supplies. Such sensors can be used to detect H_2_O_2_, pre‐ and post‐workout and pre‐ and post‐food, and alcohol consumption, hence providing information on the metabolism of the test subject. Despite their lightweight nature, all reported electrochemical H_2_O_2_ sensors suffer from slow response time, delayed analyte detection, and are not reusable (see Table S8, Supporting Information, for detailed summary of different sensors).

## 6 Epidermal Sensing

**Figure 12:**
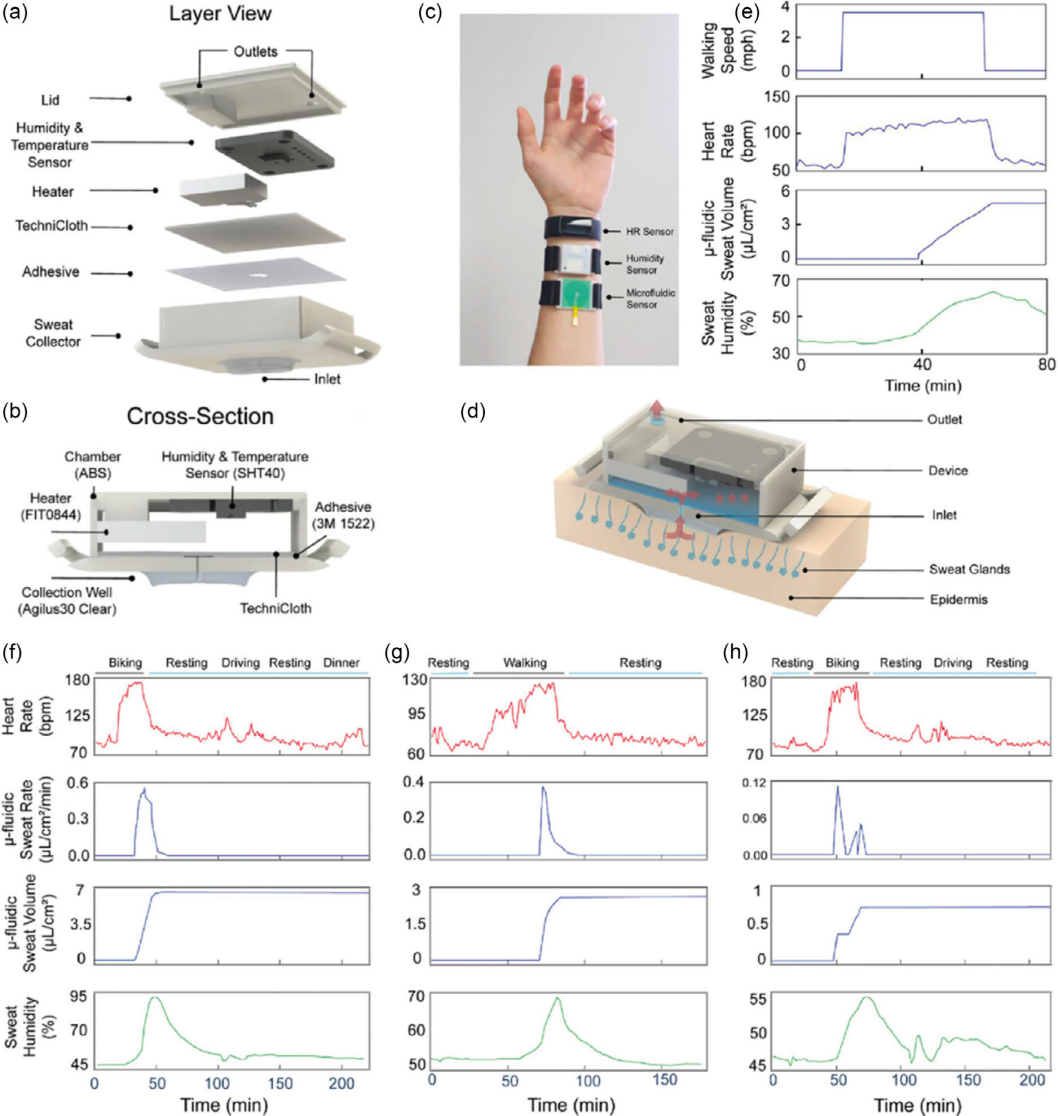
a) Schematics for a sweat‐monitoring device and its components. b) A cross section of the assembled device. c) Photograph showing a volunteer wearing a Fitbit to monitor heart rate, a humidity‐based sweat rate sensor, and a microfluidic‐based sweat rate sensor. d) Schematic illustration showing a cross‐sectional view of the interaction between the device and skin. e) Real‐time monitoring of the walking speed, heart rate, total sweat loss, and humidity throughout an entire trial of a volunteer wearing the device. f–h) The results from a 15 h, on‐body experiment where the volunteer performed normal daily activities in addition to three segments of physical activity. Reproduced with permission.^[^ ^181^ ^]^ Copyright 2023, Wiley‐VCH GmbH.

Skin is the largest organ, and it represents a great opportunity to detect biomarkers. Wristwatches, chest‐strapped heart rate monitors, glucose monitors, and blood oxygen concentration monitors have been successfully commercialized.^[^ ^158^ ^]^ Sweat is a fluid secreted by the sweat glands,^[^ ^159^ ^]^ and it contains metabolites such as lactose,^[^ ^160^, ^161^ ^]^ glucose,^[^ ^162^ ^]^ lipids,^[^ ^163^, ^164^, ^165^, ^166^ ^]^ urea,^[^ ^167^, ^168^, ^169^ ^]^ and salts. Sweat also plays a role in body temperature regulation.^[^ ^170^, ^171^, ^172^, ^173^ ^]^ Sweat rate also provides insights into hyperhidrosis or hypohidrosis states which are indicators for assessing upcoming ischemic strokes.^[^ ^174^ ^]^ The endocrine glands secrete sweat that travels through the dermis and the epidermis, reaching the top surface of the skin. Microfluidic channeled structures are commonly present on wearable skin patches, which direct the sweat into a reservoir. The collected sweat can be subjected to electrochemical, colorimetric, fluorescence, and optical transduction techniques for detecting the presence of different biomarkers.^[^ ^175^, ^176^, ^177^, ^178^, ^179^, ^180^ ^]^ Aggarwal et al. reported a wearable patch that tracks sweat rate using a commercially available humidity sensor and a microheater (**Figure** **12**).^[^ ^181^ ^]^ The device comprises a 3D‐printed acrylonitrile butadiene styrene plastic‐based humidity chamber with two holes for ventilation. The entrance of the chamber consists of a thin slice of TechniCloth that covers the floor, absorbing and spreading water molecules into the chamber (Figure 12a–d). The microheater increases the evaporation rate and the saturation vapor pressure. Consequently, observable humidity levels inside the chamber increase. Baseline humidity of sweat was measured by inserting various volumes of water into the chamber, and after subsequent microheating, the total humidity inside the chamber was measured. This device was worn on the hand, and sweat volume was measured after exercise (Figure 12c). The device allowed for real‐time tracking of the user's walking speed, heart rate, total sweat loss, and humidity (Figure 12e). Using a 15 h trial, heart rate, sweat rate, sweat volume, and sweat humidity were measured to monitor the effect of biking, resting, driving, resting, and dining on the composition of sweat (Figure 12f–h). Choi et al. reported a capacitance‐based sweat‐rate‐monitoring patch consisting of two ITO‐coated PET sheets sandwiching a laser‐cut microfluidic pathway.^[^ ^182^ ^]^ The capacitance between the two conducting plates was measured as sweat flowed through the microfluidic channel. Capacitance correlated linearly with the flow rate of sweat. An impedimetric sensing containing a microfluidic sweat collector was used for 10 h to monitor sweat rate, as reported by Bariya et al.^[^ ^183^ ^]^ The integrated electrodes within the microfluidic sweat collector measured the exact sweat rate, and once the channels were filled, a suction cup was pressed to transfer the sweat to a sweat collection chamber under negative pressure. After transferring, the chamber was reset to allow for continuous monitoring of sweat rate. An adhesion‐free, reusable digital wearable band for sweat rate monitoring was reported by Dautta et al.^[^ ^184^ ^]^ They utilized a 3D‐printed sweat collector with a concave surface strapped onto the skin.

The sweat collector was interfaced with a microfluidic channel with electrodes embedded for continuous sweat rate monitoring. One of the major advantages of this device is its reusability, as it is adhesion free. Sim et al. fabricated a portable sweat rate sensor by integrating a thermo‐pneumatic actuator, enabling humidity sensing. The pneumatic actuator allows automatic natural ventilation by lifting the humidity chamber above the skin.^[^ ^185^ ^]^ Brueck et al. recently reported a sweat rate monitor device using a microfluidic calorimetric sensor attached to a commercial sweat collector. The data was acquired using a Lilypad Simblee board and sent wirelessly to a mobile phone with a latency of 10 min before the first set of data was acquired.^[^ ^186^ ^]^ The sensor comprised a microheater sandwiched between two thermocouples. The values of temperature upstream and downstream provide information on the flow rate. Rogers and co‐workers pioneered colorimetric‐based sensors for sweat rate and sweat loss monitoring.^[^ ^187^, ^188^, ^189^, ^190^ ^]^ This skin‐integrated device consists of a microfluidic channel of a water soluble dye and as the sweat entered this channel, the dye was spread through changes in the color intensity were measured. Multimodal devices allow rapid and real‐time sensing of sweat loss, chlorides, pH, glucose, temperature, and lactate. Liu et al. recently fabricated a soft and environmentally degradable microfluidic device to monitor sweat rate and loss.^[^ ^191^ ^]^ The device could analyze sweat biomarkers using colorimetry. The microfluidic layer was fabricated using a co‐polyester elastomer attached to a transparent cellulose sealing layer at the top and a skin adhesive layer at the bottom. The serpentine microchannel allowed monitoring of the sweat rate and loss up to ≈20 μL. The sensing was visualized using a green dye placed at the inlet of the microchannel.

**Figure 13:**
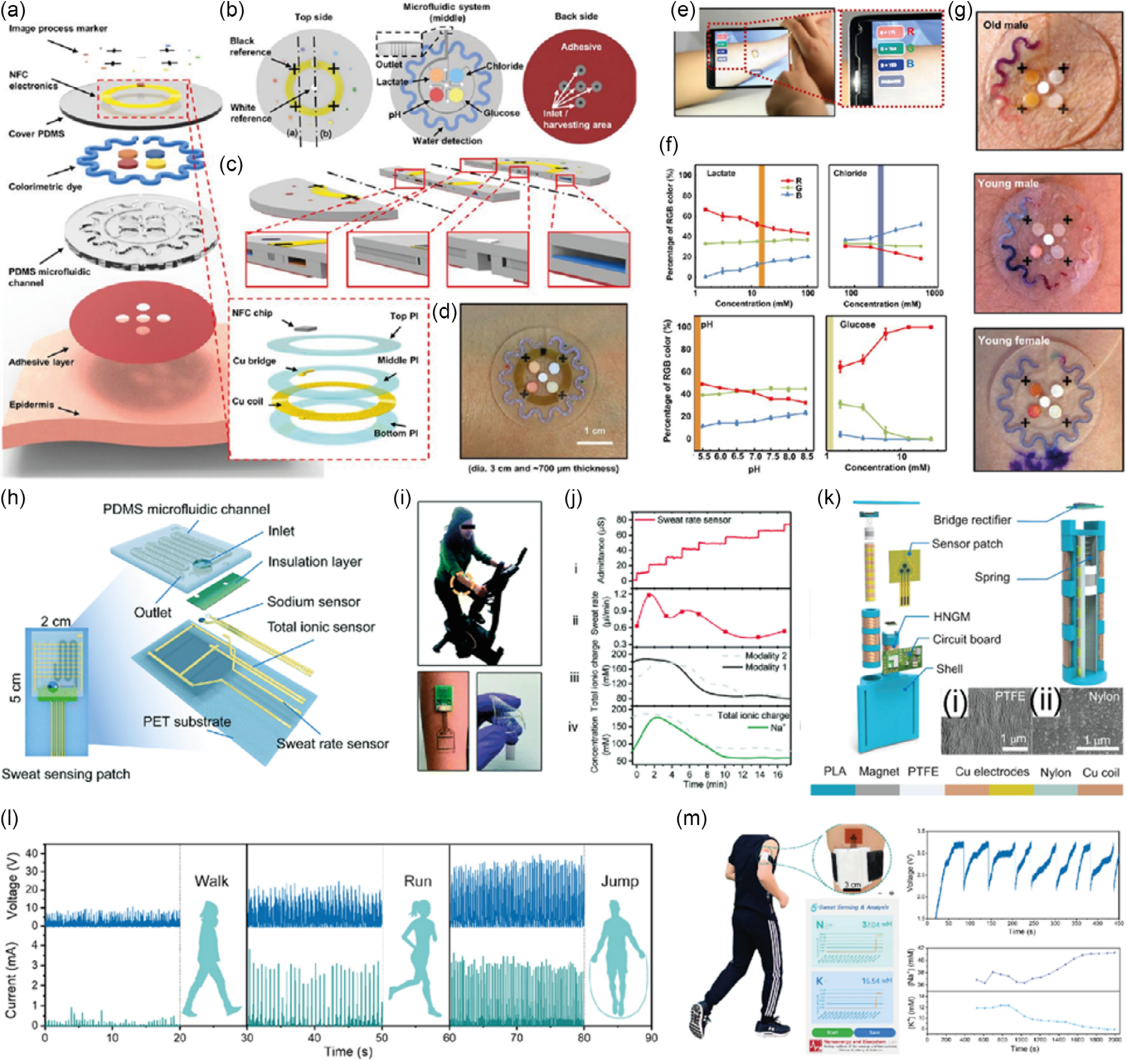
a) Schematics for a microfluidic sweat‐monitoring device and an enlarged image of the NFC system (inset). b) Schematics showing the top, middle, and back sides of the device. c) Cross‐sectional diagrams of the cuts are indicated using dashed lines. d) Optical image of a fabricated device mounted on the forearm. e) Pictures showing a smartphone used for image capture and analysis software. f) Standard calibration curves between normalized %RGB value and concentration of analytes. g) Devices on the forearms of volunteers were imaged after ≈84 km of cycling. (a–g) Reproduced with permission.^[^ ^192^ ^]^ Copyright 2016, American Association for the Advancement of Science. h) Schematics of multimodal sweat‐sensing patch. i) Photograph of a volunteer wearing a multimodal sweat‐sensing patch. j) Real‐time on‐body sensing of sweat rate and ions. (h–j) Reproduced with permission.^[^ ^193^ ^]^ Copyright 2019, Royal Society of Chemistry. k) Schematics of a self‐powered wearable sweat analysis system (SWSAS). l) Output performance of energy‐harvesting devices during walking, running, and jumping. m) Photograph of a volunteer wearing SWSAS while running, allowing for the sensing of Na^+^ and K^+^ in sweat. Reproduced with permission.^[^ ^197^ ^]^ Copyright 2022, Wiley‐VCH GmbH.

Koh et al. reported one of the earliest reports on sweat‐monitoring patches using a flexible PDMS substrate.^[^ ^192^ ^]^ The PDMS patch consisted of serpentine microfluidic channels in which the sweat was directed to reservoirs containing markers for detecting chloride, hydronium ions, glucose, and lactate (**Figure** **13**a–g). The concentration of these analytes was measured by analyzing the color changes.

These wearable patches were tested on 12 volunteers participating in the “El Tour de Tucson” bicycle tour. This experiment showed that there was no loss in the adhesion, leakage, irritation, or discomfort for the rider. Yuan et al. recently presented a multimodal wearable sweat sensor with interdigitated electrodes over a serpentine channel to measure the sweat rate and the concentration of Na^+^ ions in sweat (Figure 13h).^[^ ^193^ ^]^ As sweat passed through the channel, a discrete rise in the conductivity was noted (Figure 13i,j). Ghoorchian et al. recently reported a wearable potentiometric cell using nanostructured Na_0.44_MnO_2_ particles and Ag electrodes with a Nernstian response of 58 mV dec^−1^ in the Na^+^ concentration range of 0.21–24.54 mmol.^[^ ^194^ ^]^ The sensor was placed on the forehead of a volunteer, and the concentration of Na^+^ in the sweat was measured while the volunteer was running. The concentration of Na^+^ varied in the range of 10.4–17.7 mM. An offline inductively coupled plasma optical emission spectroscopy experiment showed a concentration of 19.8 mmol L^−1^, indicating the accuracy of the wearable device. Garcia‐Cordero et al. reported multiparameter wearable sweat analyzers using ion‐selective field‐effect transitions.^[^ ^195^ ^]^ By treating the electrode with a Na^+^ ionophore embedded inside a serpentine microfluidic channel, the sweat rate and the concentration of Na^+^ within the range of 56.7 ± 28.9 mM. The electrodes were further functionalized with a K^+^‐selective ionophore to measure K^+^ in the sweat.

**Figure 14:**
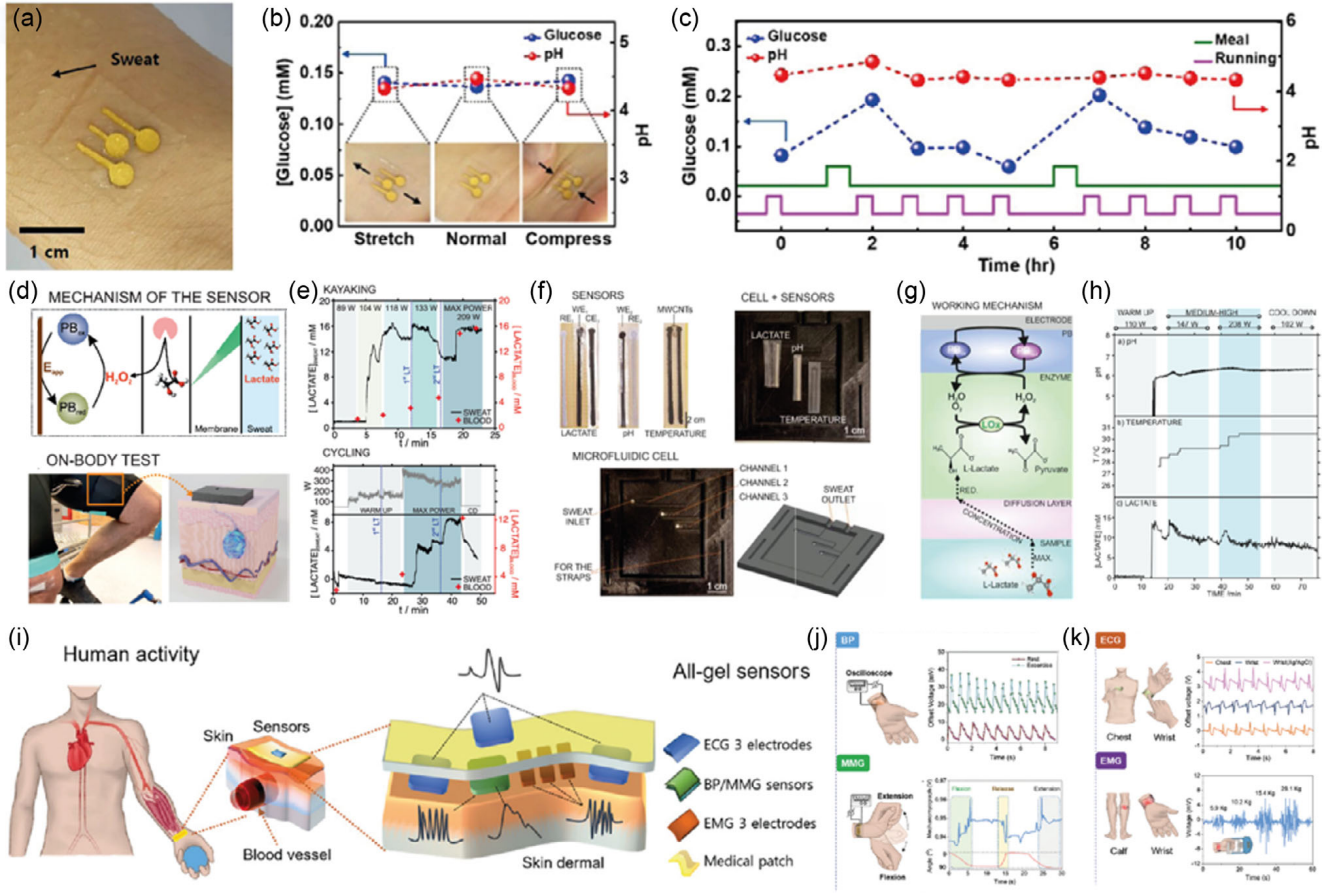
a) Photograph of an electrochemical sensor attached to the skin. b) Performance of the sensor under mechanical deformation. c) Glucose concentration and pH were measured during eating a meal (green pulse) and running (pink pulse) for 10 h. (a–c) Reproduced with permission.^[^ ^198^ ^]^ Copyright 2018, American Chemical Society. d) The mechanism for lactate‐sensing (top) and on‐body test with the subject wearing the device (bottom) in the thigh while cycling. e) The dynamic profile of lactate in sweat using maximum power during kayaking and cycling. (d,e) Reproduced with permission.^[^ ^199^ ^]^ Copyright 2023, The Author(s). Published by American Chemical Society. f) Images of lactate, pH, and temperature sensors. g) Lactate‐sensing mechanism. h) Measuring pH, lactate, and temperature during a test during stationary cycling exercise. (f–h) Reproduced with permission.^[^ ^211^ ^]^ Copyright 2021, The Author(s). Published by American Chemical Society. i) Schematics of an all‐gel sensor consisting of electrodes for ECG, electromyography (EMG), as well as a BP sensor and ECG. j) Wrist‐mounted BP sensor measurement at rest and after exercise (fast walking, top) and magnetomyography signal according to wrist angle (bottom). k) Signal obtained by ECG sensor (top) and EMG signal measured while increasing gripping force (bottom). Reproduced with permission.^[^ ^220^ ^]^ Copyright 2022, Wiley‐VCH GmbH.

Lu et al. fabricated a microsupercapacitor integrated with a self‐powered wearable fluid‐monitoring device capable of measuring glucose in the sweat with a sensitivity of 0.5 μA μM^−1^ as well as Na^+^ concentration (0.031 nF mM^−1^) and K^+^ concentration (0.056 nF mM^−1^).^[^ ^196^ ^]^ The data from the device was wirelessly transmitted to a smartphone, allowing real‐time monitoring. For the fabrication of the glucose sensor, a solution of chitosan and NiCo_2_O_4_ was drop‐casted onto the Au electrodes, and Ag/AgCl was used as the reference electrode. The incorporation of a selective ionophore allowed the detection of Na^+^ and K^+^ sweat. In another study, a self‐powered wearable system was recently reported by Gai et al.^[^ ^197^ ^]^ This system combines a TNEG and an electromagnetic generator coupled with a flexible sensor patch (Figure 13k–m). A low‐energy Bluetooth module was incorporated inside the system to transfer the data wirelessly. Laser‐induced graphitized electrodes were fabricated on a PI film and Ag/AgCl electrode was used as the reference electrode. A Na^+^‐ and K^+^‐selective ionophore was drop‐casted on the electrode, achieving sensitivities of 67.32 and 30.43 mV per decade of ion concentrations of Na^+^ and K^+^. Oh et al. reported a flexible wearable sweat patch capable of electrochemically measuring the glucose and pH of the sweat.^[^ ^198^ ^]^ The sensor consists of gold nanosheet‐based electrodes and sequential deposition of various substrates for sweat sensing (**Figure** **14**a). Glucose was measured using enzyme‐free deposition of CoWO_4_ NPs deposited over one of the pods of the electrodes. To measure pH in sweat, polyaniline was electrodeposited on another electrode. Silver nanowires were drop‐casted and treated with FeCl_3_, hence becoming Ag/AgCl reference electrode. Real‐time glucose concentration and sweat pH were measured continuously for 10 h (Figure 14b,c). It was shown that glucose concentration increased steadily after meal consumption. However, there was no significant decrease in sweat pH, presumably due to maintaining homeostasis.

A disposable enzyme‐based sweat glucose sensor that can electrochemically measure the concentration in sweat, heart rate, and blood oxygen saturation level would provide a comprehensive health management system.^[^ ^199^ ^]^ Xiao et al. ingeniously combined a plasma treated cotton thread to direct sweat toward a functionalized filter paper for sensing glucose calorimetrically.^[^ ^200^ ^]^ This was achieved by sequentially treating the paper with chitosan, glucose oxidase, horseradish peroxidase, and 3,3,5,5‐tetramethylbenzidine. The device showed a detection limit of ≈35 μM in the dynamic range of 50–250 μM. In another study, it was demonstrated that a polypyrrole polymerized conducting cotton fabric treated with copper acetate and manganese acetate to electrochemically detect glucose in sweat with a correlation *R* ^2^ = 0.973. The sensor remained accurate even after 60 days of use.^[^ ^201^ ^]^ Furthermore, graphene oxide and aminofullerene were used to detect and quantify the presence of several ions in sweat.^[^ ^202^ ^]^ Replacing aminofullerene with copper nanowires enabled glucose detection in sweat with a rapid response time of 5 s and a sensitivity of 1020 μA mM^−1^ cm^2^.^[^ ^203^ ^]^

Dervisevic et al. reported a sensitive sweat glucose sensor based on a Si‐based micropillar array patch with a sensitivity of 4.7 ± 0.8 μA mM^−1^ and an LOD of 26 ± 5 μM.^[^ ^204^ ^]^ The sensor consisted of over 70 000 micropillar arrays treated with Prussian blue and chitosan‐Au NPs, followed by immobilization of glucose oxidase. Importantly, the sensor lost 95% of its initial response after just four hours of use when a flat surface without any micropillar arrays was used.

Lactate is a byproduct of glycolysis during anaerobic exercise and can be detected in sweat.^[^ ^205^ ^]^ It has been shown that critically ill patients often show increased blood lactate levels, known as hyperlactatemia. Hyperlactatemia has clinical implications for the mortality and morbidity of the patients. Therefore, rapid and noninvasive measurements of lactate from various sources offer clinical significance.^[^ ^206^, ^207^ ^]^ Integrating commercially available lactate strips (Lactate Pro‐2) inside a single‐use sweat sensor provided a correlation between blood and sweat lactate concentration with an error of 0.3 mM.^[^ ^208^ ^]^ In another report, an electrochemical lactate sensor was integrated inside a microfluidic channel for noninvasively monitoring lactate concentrations with a sensitivity of −12.5 ± 0.53 nA mM^−1^ and a response time of <90 s (Figure 14d).^[^ ^209^ ^]^ Upon interaction with lactate, lactate oxidase produces hydrogen peroxide and pyruvate. Released hydrogen peroxide interacts with Prussian blue, thereby decreasing the output current. This change in current is proportional to the concentration of lactate in sweat. The on‐body test while cycling or on maximal power while kayaking or cycling allows for creating a dynamic profile of sweat lactate (Figure 14e). Currano et al. fabricated a flexible lactate sensor using a Kapton patch on which electrodes were printed using lactate oxidase enzyme and an organic electrochemical transistor was used to measure lactate concentrations below 1.0 mM.^[^ ^210^ ^]^ However, there is no clear correlation between sweat and blood lactate concentrations. Therefore, further understanding is needed for noninvasive lactate monitoring. Xuan et al. fabricated a lactate, pH, and temperature sensor by amperometry, potentiometry, and resistance readouts. The sensor was placed inside an epidermal patch containing microchannels to direct the flow of sweat (Figure 14f–h).^[^ ^211^ ^]^ The sensor used Prussian blue as a redox mediator and lactate oxidase as the activating enzyme. The pH sensor was fabricated using PANi electrodes and temperature was measured using CNTs.^[^ ^212^ ^]^ Huang and co‐workers incorporated six biosensors, NH_4_^+^, Na^+^, glucose, pH, skin impedance, and temperature, inside a wearable skin‐monitoring system having LEDs and mechanical vibrations to indicate physiological signals instead of transferring response wirelessly needing additional equipment.^[^^165^^]^

Ethyl glucuronide is a non‐oxidative metabolite produced by the liver when ethanol interacts with glucuronic acid. Ethyl glucuronide is a water‐soluble compound that is present in sweat, urine, and the hair.^[^ ^213^, ^214^, ^215^ ^]^ Prasad and co‐workers reported the noninvasive detection of ethyl glucuronide by monitoring sweat.^[^ ^216^ ^]^ They deposited Au electrodes and ZnO electrodes on a flexible PI substrate. Antibodies were immobilized on the electrodes for the detection of ethyl glucuronide at concentrations of 0.001–100 μg L^−1^. Interestingly, the rigid glass substrate allowed for detecting a low concentration of 0.0001 μg L^−1^. Anastasova et al. utilized a wearable multisensing patch containing microfluidic channels and reservoirs to continuously detect lactate, pH, and Na^+^ ions in sweat.^[^ ^217^ ^]^ Recently, Yoon et al. developed potentiometric ion‐sensing threads using a bio‐based supramolecular polymer coated over carbon fibers.^[^ ^206^ ^]^ The supramolecular polymer matrix was prepared using citric acid, succinic acid, and cyclohexanedimethanol. The thread was knitted into a fabric to sense K^+^/Na^+^ concentrations in sweat. The thread sensor displayed rapid self‐healing within 30 s at room temperature and recovered up to 97% of its efficiency after healing. This self‐healing wearable sensor thus allowed for real‐time sweat monitoring of volunteers during exercise. However, further optimization is necessary to reduce the fabrication steps overall design to improve comfort for the user, improve repeatability, and access to sweat for the measurement (see Table S9, Supporting Information, for comparison of different sensors).

An ECG is an important diagnostic tool used to measure the electrical signals of the heart and is used to determine the overall health of the heart. ECGs are obtained by placing Ag/AgCl electrodes on the skin using an electrolyte gel. However, traditionally, the functioning of the heart is monitored while the patient is stationary.^[^ ^217^ ^]^ Wearable ECG sensors aim to overcome the requirement of electrolyte gel to connect the electrode to the skin.^[^ ^218^, ^219^ ^]^ Recently, Chun et al. reported a wearable multimodal sensor for monitoring blood pressure, ECG, electromyograms, and mechanomyograms (Figure 13i).^[^ ^220^ ^]^ A composite ionic gel of PANi–poly (vinyl chloride) was used as a slow‐adapting‐sensing material for measuring blood pressure (BP) and ECG. A polyvinylidene fluoride–trifluoroethylene gel was used for rapid adapting sensing and for detecting mechanomyograms (Figure 14i). To measure these key features, they connected the Ag electrode to the gel and measured the corresponding signal using an oscilloscope (Figure 14j). The ECG signals obtained from the chest and wrist were comparable to those obtained using standard Ag/AgCl electrodes (Figure 14k).

**Figure 15:**
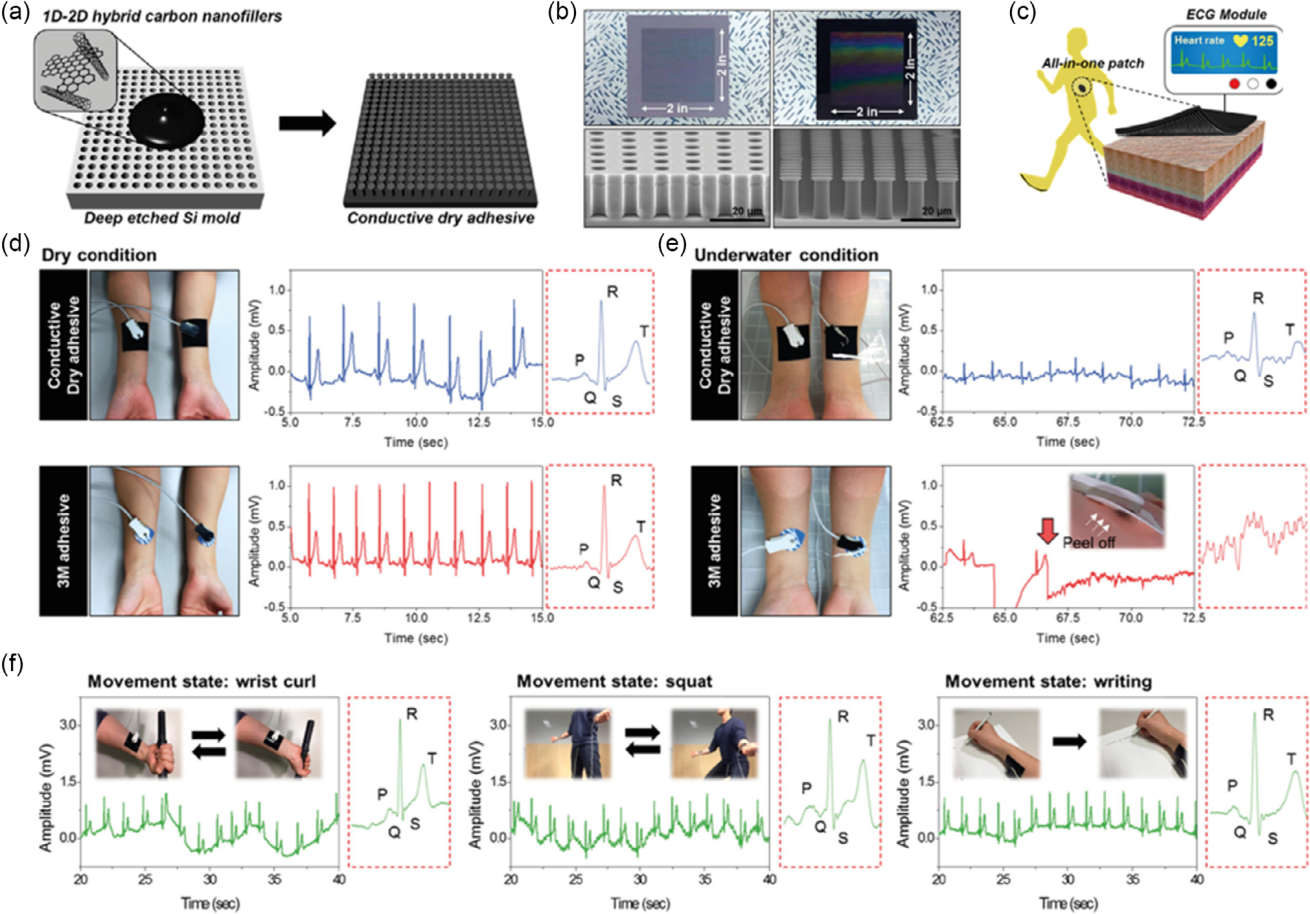
a) Schematic illustration of the fabrication for conductive dry adhesives and their application as ECG electrodes. b) Digital images of a silicon mold with gecko‐inspired micropatterns and a conductive dry adhesive, c) wearable patch, and d) comparison between ECG signals using conductive dry adhesives and commercial electrodes with 3M adhesives. e) Comparison of the ECG signals using conductive dry adhesives and commercial electrodes with 3M adhesives under water. f) Series of ECG signals using conductive dry adhesives under various movement conditions. Reproduced with permission.^[^ ^221^ ^]^ Copyright 2016, American Chemical Society.

Even though there have been multiple reports on wearable sensors for monitoring these vital biomarkers, appropriate guidelines for correlations between these signals and muscle movement are still limited. Utilizing ML to extract key features would enable the scientific community to come to a necessary conclusion for continuous monitoring and health prediction. Kim et al. recently reported a conductive dry adhesive wearable patch for measuring ECG (**Figure** **15**).^[^ ^221^ ^]^ This highly flexible sensor possesses a gecko‐like hierarchical structure a conducting filler with CNTs and graphene inside a PDMS elastomer matrix. The sensor had an overall conductivity of around ≈0.01 S cm^−1^, allowing it to capture biosignals from the human skin. As the electrolyte gel was absent, ECG signals could be monitored underwater. Recently, Wang et al. reported a self‐healing electronic tattoo (E‐tattoo) using graphene flakes suspended in silk fibroin/Ca^2+^. The tattoo could detect strain, humidity, temperature, and breathing responses.^[^ ^222^ ^]^ (EEG are considered the gold standard for studying brain disorders.^[^ ^223^ ^]^ Similar to electrodes used in ECG, electrodes for EEG are also classified into wet, dry, semidry, and hydrogel‐based electrodes. Conventional electrodes need an electrolyte gel to ensure good contact with the skin.^[^ ^224^ ^]^ Leleux et al. developed a flexible electrode for measuring EEG. The sensor was fabricated by sputtering Au on a Kapton tape substrate followed by a layer of a conducting polymer PEDOT:PSS.^[^ ^225^ ^]^ The frequency analysis plot showed that the performance of PEDOT:PSS‐coated electrodes was comparable to that of commercial Ag/AgCl electrodes. Since there is no need for gel electrolytes, such wearable electrodes could be worn longer. Chen et al. designed wearable, comfortable dry electrodes with an MN structure that does not penetrate the skin to monitor EEG.^[^ ^226^ ^]^ They used an ethylene propylene diene monomer mixed with additives such as carbon, stainless steel fibers, and carbon nanofibers under compression molding. The EEG spectra for these electrodes displayed comparable results under wet and dry conditions, but dry electrodes offered user‐friendliness and minimized training.^[^ ^227^, ^228^, ^229^ ^]^ Recently, Carvalho et al. fabricated a nondrying and skin‐adhering glycerol–polyacrylamide hydrogel. These hydrogel electrodes showed an impedance (2.43 × 10^5^ Ω) lower than that of conventional Ag/AgCl (6.61 × 10^5^ Ω) electrodes.^[^ ^230^ ^]^

Hydrogel‐based electrodes are skin conformable, have better adhesion, and are highly stretchable.^[^ ^231^ ^]^ Despite these advantages, hydrogels suffer from higher impedance, and dehydration increases their brittleness and rigidity.^[^ ^232^ ^]^ Peng et al. fabricated a microporous titanium (Ti)‐based passive electrode system for EEG recording.^[^ ^233^ ^]^ The Ti was encapsulated inside a PDMS case that contained KCl and AgCl solutions, which acted as electrolytes. The electrolyte was released after applying pressure on the PDMS casing, and the EEG signal was recorded. The electrolyte solution did not cause any irritations or allergic reactions to the skin. Even though the results were interesting, the performance did not match the standards of Ag/AgCl electrodes.^[^ ^233^ ^]^ The reported ECG and EEG sensors display poor conductivity under excessive perspiration, low performance under movements, and delayed response (see Table S10, Supporting Information, for a detailed summary of the performance of various ECG/EEG sensors).

## 7 MN‐Based Platforms

**Figure 16:**
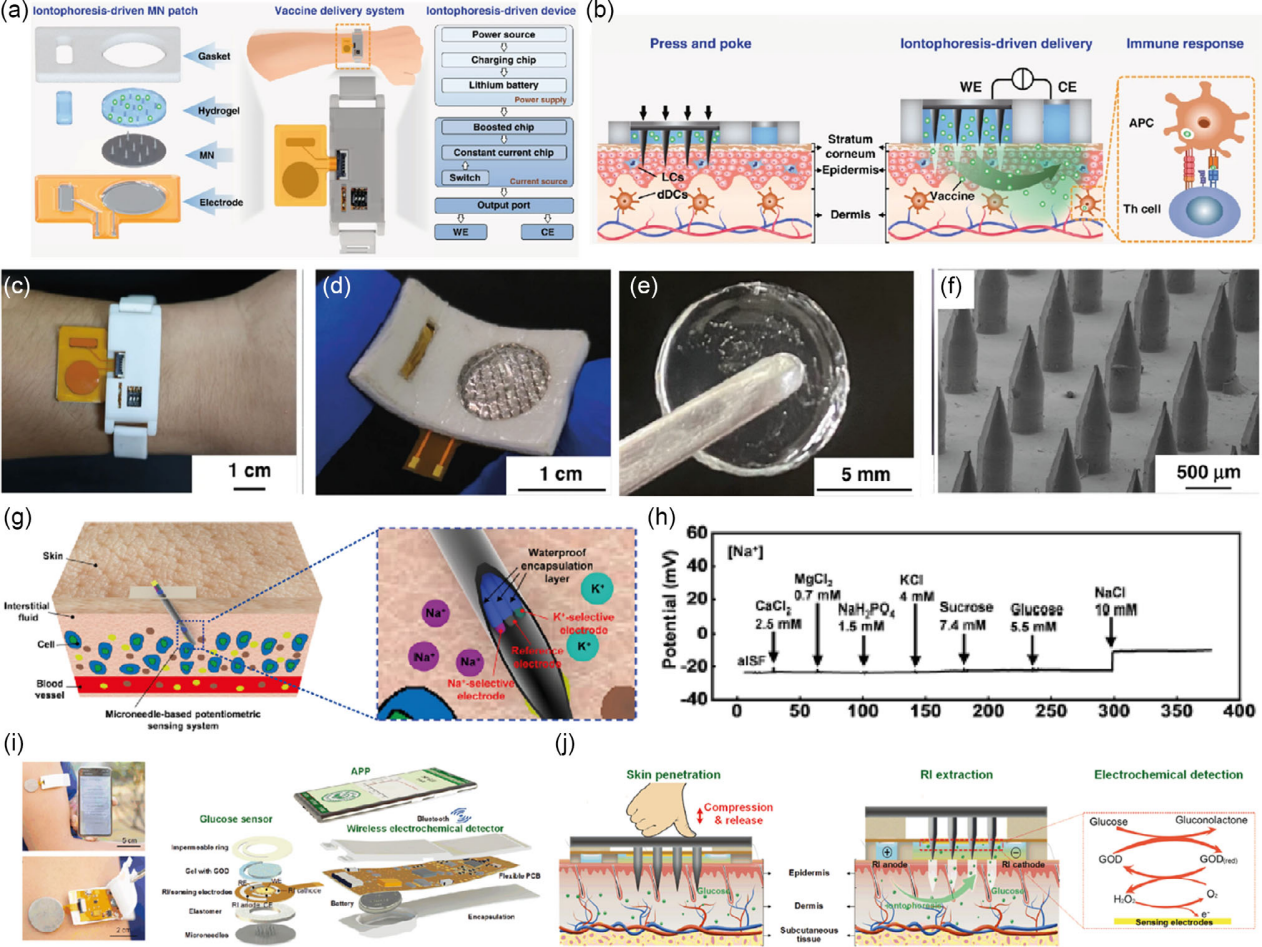
a) Schematics showing the iontophoresis‐driven microneedle patch, its components and vaccine delivery device. b) Schematics of the working principle of iontophoresis‐driven delivery and immune response. c) Photograph of the device worn on a wrist. d) Photograph of a flexible MN patch. e) Image of the hydrogel loaded with vaccine. f) SEM image of the MNs. Reproduced under the terms of the Creative Commons Attribution 4.0 International License.^[^ ^238^ ^]^ Copyright 2023, The Author(s). Published by Springer Nature. g) Schematics showing simultaneous monitoring of Na^+^ and K^+^ in skin interstitial fluid (ISF) using MN‐based potentiometric sensor. h) The effect of various salts and sugars on the selectivity of the Na^+^ sensor. Reproduced with permission.^[^ ^246^ ^]^ Copyright 2021, American Chemical Society. i) Smartphone‐based glucose electrochemical detection platform and schematics of the components of glucose sensor and wireless electrochemical detection devices. j) The working principle of glucose extraction and amperometric detection of glucose in the ISF using reverse iontophoresis. Reproduced with permission.^[^ ^247^ ^]^ Copyright 2022, Elsevier Inc.

In recent years, MN systems have emerged as promising biomedical devices for transdermal delivery of drugs, vaccines, and biomolecules. The MN platforms, such as patches, consist of an array of MNs of 50–90 μm in diameter with varying lengths between 25 and 2000 μm, allowing skin penetration without neural tissue damage.^[^ ^234^ ^]^ They offer controlled and on‐demand drug delivery, health parameter monitoring, pain management, biofluid extraction, and disease diagnostics. Depending on the desired application, solid, coated, dissolving, and hollow MNs have been fabricated.^[^ ^235^, ^236^ ^]^ The solid MN patches are used as pretreatment on the skin. Hollow MNs are miniature hypodermal needles suitable for drug delivery. The dissolving needles comprise biodegradable materials which dissolve upon contact with the skin and release the payload into the skin. The coated MNs are fabricated by coating the solid needle with a target drug solution or dispersion. Unlike conventional hypodermic injection, MNs are minimally invasive and can be used for self‐administered transdermal delivery of drugs.^[^ ^237^ ^]^ Zheng et al. recently reported a wearable iontophoresis‐driven MN patch for active transdermal vaccine delivery.^[^ ^238^ ^]^ Briefly, it consists of a solid MN array fabricated by milling 316 L stainless steel (**Figure** **16**). Additionally, it consists of Ag/AgCl electrode coated on a flexible PI film and gasket to hold each component firmly (Figure 16a–f). The MN array was used to puncture the conductive polyacrylamide/chitosan hydrogel loaded with vaccine and the skin. The vaccine from the hydrogel slowly diffuses from the hydrogel into the skin via the microchannels by applying a small potential. The delivery rate was controlled by the microchannel formation, current, duration, and dimension.^[^ ^239^ ^]^ The performance of these patches suggested that the MN platform induced a stronger immune response than the traditional intramuscular injection.

Modifying the solid MN array with porous interconnect micropores enhanced the molecular transport and improved the iontophoresis by direct current through the skin, which lowers the transdermal resistance of the stratum corneum.^[^ ^240^ ^]^ Furthermore, it can transport larger molecules via the interconnected micropores and generate electroosmotic flow. Duong et al. reported a polycarbonate‐based MN platform coated with polyelectrolyte multilayer assembly of charge reversal pH‐sensitive polymer for efficiently administering DNA‐based polyplex vaccines.^[^ ^241^ ^]^ Meyer et al. investigated the preclinical feasibility of first‐generation Nanopatch and the immune response of the unadjuvanted human papillomavirus vaccine in rhesus macaques.^[^ ^242^ ^]^ The immune response observed in Nanopatch was similar to that of traditional intramuscular administration. As a vaccine delivery system, MN technology has proven to be a viable alternative to conventional intramuscular injection in preclinical trials for influenza vaccinations, as it has demonstrated comparable immunological responses.^[^ ^243^ ^]^ MNs assist in treating massive submuscular hemorrhages^[^ ^244^ ^]^ andcentral retinal artery occlusion.^[^ ^245^ ^]^

Recently, Li et al. fabricated an MN‐based potentiometric sensor for rapid and reversible skin interstitial fluid electrolyte monitoring (Figure 16g).^[^ ^246^ ^]^ The system includes a sodium‐ion‐selective electrode, a potassium‐ion‐selective electrode, and an Ag/AgCl reference electrode housed within a stainless‐steel hollow MN. The MN‐based sensor allowed the detection of multiple electrolytes, including KCl (4 mM), NaCl (10 mM), CaCl_2_ (2.5 mM), MgCl_2_ (0.7 mM), NaH_2_PO_4_ (1.5 mM), sucrose (7.4 mM), and glucose (5.5 mM) from a pool of artificial ISF fluid complex (Figure 16h). The hollow MN structure ensures the robust operation of the sensor during in vivo monitoring, providing reliable results and preventing any delamination issues during insertion. Cheng et al. demonstrated a touch‐activated glucose sensor by integrating a solid MN array with a reverse iontophoresis sampling unit and an electrochemical sensing unit (Figure 16i).^[^ ^247^ ^]^ The MN array allows minimally invasive skin penetration, creating microchannels to extract glucose from the ISF fluid. The glucose sensor consists of glucose oxidase encapsulated in agarose gel, the sensing electrodes, and an elastomer ring to supply the necessary force for solid MN to penetrate the skin. Furthermore, the sensor performance was demonstrated in vivo using a mouse model and the data was recorded on the smartphone‐based app. The performance was comparable to a commercial glucometer.

**Figure 17:**
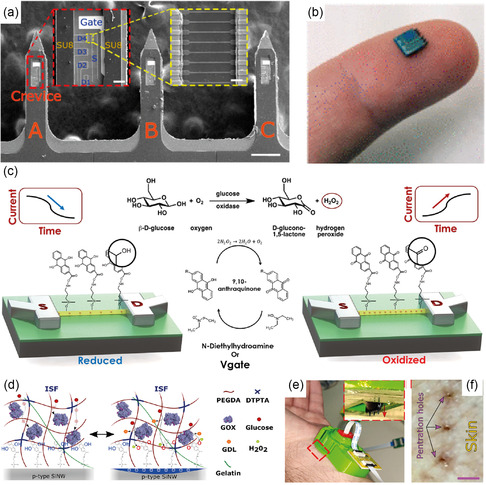
a) SEM image of silicon nanowire‐array‐based microneedle chip. b) Photograph of a microneedle chip on a finger. c) Schematics show the working principle of sensing, chemical modification of the surface, and reversible redox reactions. d) Schematic representation of hydrogel immobilized with enzymes and catalytic reaction. e) Photograph of 3D printed (inset shows a zoomed image of the microneedles penetrating through the patch) of oral glucose tolerance testing (OGTT). f) Photograph of the penetration holes in the skin after the OGTT test (scale bar: 650 μm). Reproduced with permission.^[^ ^248^ ^]^ Copyright 2021, American Chemical Society.

Heifler et al. recently fabricated a chemically modified silicon nanowire (SiNW) FET as a nanosensor on MN system for the detection of metabolites in the ISF and insulin delivery (**Figure** **17**).^[^ ^248^ ^]^

The device consists of a depth self‐limiting Si MN hosting four SiNW–FET devices for detecting glucose and lactate in the ISF (Figure 17a,b). The surface of these SiNW was chemically treated with amino silanes to covalently link the redox‐active anthraquinone molecule. The redox‐active molecules, when interacting with oxidizing agents such as H_2_O_2_, induce a change in the conductivity due to the altered electric field of SiNW (Figure 17c,d). A hydrogel made from poly(ethylene glycol) diacrylate with a matrix of diethylenetriaminepentaacetic acid linkers immobilized with glucose and lactate oxidase enzymes were applied to where the SiNW–FETs‐sensing devices are located. This allowed the diffusion of glucose and lactate molecules from the interstitial fluid and enzymatic oxidation. The oxidation produces H_2_O_2_ and glucono delta lactone, which oxidizes the anthraquinone moieties on the surface of the sensor. More importantly, the studies were extended for in vivo testing using healthy human subjects and the mice model (Figure 17e). Such MN platforms provide assistance for continuous monitoring of levodopa for Parkinson's management,^[^ ^249^ ^]^ nerve agent detection,^[^ ^250^ ^]^ biofluid extraction,^[^ ^251^, ^252^, ^253^ ^]^ and therapeutic drug monitoring.^[^ ^254^ ^]^ In addition to controlled transdermal delivery of drugs, it also offers long‐term usage and a lengthy shelf life. Continued research is centered around optimizing the needle design to minimize incision points through careful adjustments of its geometry. Additionally, closed loop systems are being explored along with efficient wireless transfer data to mobile phone applications. Enabling human usage after approval from medical agencies and allowing the large‐scale affordable production of these MN systems would be a significant breakthrough.

## 8 ML for Sensor Data Analysis

Wearable sensors collect immense amounts of data that need further processing to extract clinically relevant information. Management of this massive amount of information is challenging. In this context, ML has become a tool of choice for extracting meaningful data, discovering hidden patterns, and predicting diseases.^[^ ^255^ ^]^ ML involves collecting raw data from the sensors and subjecting this data to normalization, smoothing, and denoising. A typical neural network consists of input, output, and several hidden layers depending on the predictive capabilities of the network.^[^ ^256^ ^]^ Some of the widely used architectures reported for analyzing wearable sensor data are convolutional neural networks, recurrent neural networks, and transformer networks. Support vector machine is one of the classifiers associated with supervised learning models. Other models include bag of symbolic Fourier approximation (SFA), long short‐term memory, dynamic time wrapping, symbolic aggregate approximation in vector space model, piecewise aggregate approximation, bag of slow feature analysis symbols in vector space and symbolic function approximation (SFA), decision tree, random forest, and extra tree. The accuracy of these algorithms can be further improved by increasing the size of the training data. However, larger sets of data increase the overall processing time for the algorithm. Accuracy can also be enhanced by increasing the number of hidden layers which would demand higher processing power. There is no simple solution in this context, but a careful compromise between memory capacity, processing power, and data set size is essential.

## 9 Conclusions

Biofluids such as teardrops, sweat, interstitial fluids, and exhaled breath condensate offer a promising avenue for noninvasive diagnostics. Due to the abundance of biomarkers in various biofluids, identifying tools for rapid sensing and analysis provides transformative impacts and advancements in healthcare. More importantly, correlations between blood metabolites and metabolites in other biofluids are becoming increasingly available so that noninvasive tools can overcome the laborious, invasive, and cost‐intensive blood‐based assays. First‐generation wearable sensors, such as wristwatches and fitness bands, allow for biometric measurements, activity tracking, sleep monitoring, breathing rate, and heart rate. However, these first‐generation sensors have several limitations when used in advanced health‐condition‐monitoring applications. For example, they are often designed for single‐purpose use, display limited functionality, and have low battery life and low accuracy. Second‐generation sensors can be readily integrated into various pieces of equipment, including contact lenses, face masks, fitness bands, and fabrics. However, these integrations require that the sensors are lightweight, biocompatible, and nontoxic. Despite numerous efforts, comfort, accuracy, durability, and cost remain significant challenges to realize their wide use.

Wearable devices such as smart contact lenses also face numerous regulatory challenges. The direct contact of such devices with various organs also poses challenges due to the strict traceability of the materials utilized in the fabrication of these devices. For example, metal ion leaching, oxidation, and unwanted chemical reactions may pose significant challenges to overcome before clinical trials. Since smart wearable sensor data are often collected in smartphones, there are data‐privacy‐ and security‐related challenges. We are optimistic about ongoing research trends as overcoming current challenges may foster unprecedented progress in personalized diagnosis and treatment. More importantly, though the sensors will provide data similar to those obtained via blood diagnostics, one should view them as a complementary approach instead of replacing the existing methods. Massive clinical trials are needed for a successful deployment to assess the accuracy of wearable sensors for diagnostics and health prediction.

## Conflict of Interest

The authors declare no conflict of interest.

## Supporting information

Supplementary Material

## References

[1] D. Erlangga , T. Powell‐Jackson , D. Balabanova , K. Hanson , BMJ Glob. Health. 2023, 8, e012562. 10.1136/bmjgh-2023-012562 PMC10689394 38035736

[2] J. Piscopo , W. Groot , M. Pavlova , PLoS ONE 2024, 19, e0299359. 38446804 10.1371/journal.pone.0299359 PMC10917289

[3] S. Hamidi , Risk Manage Healthc. Policy 2014, 7, 163. 10.2147/RMHP.S69868 PMC4181628 25285027

[4] J. A. Price , L. Guinness , W. Irava , I. Khan , A. Asante , V. Wiseman , Health Policy Plan. 2016, 31, 472. 26420641 10.1093/heapol/czv089

[5] C. J. Murray , R. Govindaraj , P. Musgrove , Bull. World Health Organ. 1994, 72, 623. 7923542 PMC2486604

[6] M. T. Schneider , A. Y. Chang , A. Chapin , C. S. Chen , S. W. Crosby , A. C. Harle , G. Tsakalos , B. S. Zlavog , J. L. Dieleman , BMJ Glob. Health 2021, 6, e005799. 10.1136/bmjgh-2021-005799 PMC8327839 34330760

[7] C. McMurtry , M. Noel , C. Chambers , P. McGrath , Health Psychol. 2011, 30, 780. 21806301 10.1037/a0024817

[8] J. V. Vaghasiya , C. C. Mayorga‐Martinez , M. Pumera , NPJ Flex. Electron. 2023, 7, 26. 37304907 10.1038/s41528-023-00261-4 PMC10237062

[9] Ever Easier Health Monitoring, Nat. Biomed. Eng. 2023, 7, 1205. 37848560 10.1038/s41551-023-01126-x

[10] D. K. Ming , S. Sangkaew , H. Q. Chanh , P. T. H. Nhat , S. Yacoub , P. Georgiou , A. H. Holmes , Int. J. Infect. Dis. 2020, 96, 648. 32497806 10.1016/j.ijid.2020.05.086 PMC7263257

[11] K. Bayoumy , M. Gaber , A. Elshafeey , O. Mhaimeed , E. H. Dineen , F. A. Marvel , S. S. Martin , E. D. Muse , M. P. Turakhia , K. G. Tarakji , M. B. Elshazly , Nat. Rev. Cardiol. 2021, 18, 581. 33664502 10.1038/s41569-021-00522-7 PMC7931503

[12] R. S. Kalash , V. K. Lakshmanan , C. S. Cho , I. ‐K. Park , in Biomaterials Nanoarchitectonics (eds. M. Ebara ), William Andrew Publishing, Norwich, NY 2016, p. 197.

[13] D. Powell , A. Godfrey , NPJ Digit. Med. 2023, 6, 70. 37087520 10.1038/s41746-023-00820-z PMC10122642

[14] X. Wang , B. Yang , J. Liu , Y. Zhu , C. Yang , Q. He , Sci. Rep. 2016, 6, 36409. 27805065 10.1038/srep36409 PMC5090987

[15] J. Xu , D. Xu , X. Xuan , H. He . Molecules 2021, 26, 5912 34641460 10.3390/molecules26195912 PMC8512585

[16] S. S. Barold , Card. Electrophysiol. Rev. 2003, 7, 99. 12766530 10.1023/a:1023667812925

[17] C. Fisch , J. Am. Coll. Cardiol. 2000, 36, 1737. 11092639 10.1016/s0735-1097(00)00976-1

[18] W. Ormerod , J. Med. Biogr. 2006, 14, 30. 16435031 10.1258/j.jmb.2006.04-22

[19] E. K. S. Louis , L. C. Frey , J. W. Britton , J. L. Hopp , P. Korb , M. Z. Koubeissi , W. E. Lievens , E. M. Pestana‐Knight , E. K. S. Louis , in Electroencephalography (EEG): An Introductory Text and Atlas of Normal and Abnormal Findings in Adults, Children, and Infants (Eds: E. K. S. Louis , L. C. Frey ) American Epilepsy Society, Chicago, IL 2016. 27748095

[20] S. M. A. Iqbal , I. Mahgoub , E. Du , M. A. Leavitt , W. Asghar , NPJ Flex. Electron. 2021, 5, 1. https://doi.org/10.1038/s41528‐021‐00107‐x.

[21] V. L. D. S. N. Button , in Principles of Measurement and Transduction of Biomedical Variables, Academic Press, Cambridge, MA 2015, pp. 77–99.

[22] E. G. Moser , L. B. Crew , S. K. Garg , Adv. Diabetol. 2010, 26, 73.

[23] D. M. Nathan , S. Genuth , J. Lachin , P. Cleary , O. Crofford , M. Davis , L. Rand , C. Siebert , New. Engl. J. Med. 1993, 329, 977. 8366922 10.1056/NEJM199309303291401

[24] X. Lin , Y. Xu , X. Pan , J. Xu , Y. Ding , X. Sun , X. Song , Y. Ren , P.-F. Shan , Sci. Rep. 2020, 10, 14790. 32901098 10.1038/s41598-020-71908-9 PMC7478957

[25] O. Aquilina , Images Paediatr. Cardiol. 2006, 8, 17. 22368662 PMC3232561

[26] J. W. Severinghaus , Y. Honda , J. Clin. Monit. 1987, 3, 135. 3295125 10.1007/BF00858362

[27] P. C. Nicolson , J. Vogt , Biomaterials 2001, 22, 3273. 11700799 10.1016/s0142-9612(01)00165-x

[28] V. Andoralov , S. Shleev , T. Arnebrant , T. Ruzgas , Anal. Bioanal. Chem. 2013, 405, 3871. 23392407 10.1007/s00216-013-6756-x

[29] D.-H. Kim , N. Lu , R. Ma , Y.-S. Kim , R.-H. Kim , S. Wang , J. Wu , S. M. Won , H. Tao , A. Islam , K. J. Yu , T. Kim , R. Chowdhury , M. Ying , L. Xu , M. Li , H.-J. Chung , H. Keum , Science 2011, 333, 838. 21836009 10.1126/science.1206157

[30] F. Güder , A. Ainla , J. Redston , B. Mosadegh , A. Glavan , T. J. Martin , G. M. Whitesides , Angew. Chem. Int. Ed. 2016, 55, 5727. 10.1002/anie.201511805 27059088

[31] F. Yi , X. Wang , S. Niu , S. Li , Y. Yin , K. Dai , G. Zhang , L. Lin , Z. Wen , H. Guo , J. Wang , M.-H. Yeh , Y. Zi , Q. Liao , Z. You , Y. Zhang , Z. L. Wang , Sci. Adv. 2016, 2, e1501624. 27386560 10.1126/sciadv.1501624 PMC4928980

[32] S. P. Shashikumar , A. J. Shah , Q. Li , G. D. Clifford , S. Nemati , IEEE EMBS Int. Conf. Biomed. Health Inform. 2017, 141.

[33] T. Y. Kim , J. W. Mok , S. H. Hong , S. H. Jeong , H. Choi , S. Shin , C.-K. Joo , S. K. Hahn , Nat. Commun. 2022, 13, 6801. 36357417 10.1038/s41467-022-34597-8 PMC9649789

[34] T. M Rawson , S. A. N. Gowers , D. M. E. Freeman , R. C. Wilson , S. Sharma , M. Gilchrist , A. MacGowan , A. Lovering , M. Bayliss , M. Kyriakides , P. Georgiou , A. E. G. Cass , D. O’Hare , A. H. Holmes , Lancet 2019, 1, E335. 10.1016/S2589-7500(19)30131-1 33323208

[35] S. E. Reaume , Science 1952, 116, 641. 13028236 10.1126/science.116.3023.641

[36] S. Hashmi , P. Ling , G. Hashmi , M. Reed , R. Gaugler , W. Trimmer , Biotechniques 1995, 19, 766. 8588914

[37] S. Henry , D. V. McAllister , M. G. Allen , M. R. Prausnitz , J. Pharm. Sci. 1998, 87, 922. 9687334 10.1021/js980042+

[38] H. C. Ates , P. Q. Nguyen , L. Gonzalez‐Macia , E. Morales‐Narváez , F. Güder , J. Collins , C. Dincer , Nat. Rev. Mater. 2022, 7, 887. 35910814 10.1038/s41578-022-00460-x PMC9306444

[39] S. Qiu , Y. Cai , H. Yao , C. Lin , Y. Xie , S. Tang , A. Zhang , Signal Transduct. Target. Ther. 2023, 8, 132. 36941259 10.1038/s41392-023-01399-3 PMC10026263

[40] J. R. Sempionatto , J. A. Lasalde‐Ramírez , K. Mahato , J. Wang , W. Gao , Nat. Rev. Chem. 2022, 6, 899. 37117704 10.1038/s41570-022-00439-w PMC9666953

[41] A. A. Smith , Z. T. H. Tse , Sci. Rep. 2023, 13, 4998. 36973262 10.1038/s41598-022-26951-z PMC10043012

[42] S. Campuzano , M. Pedrero , R. M. Torrente‐Rodríguez , J. M. Pingarrón , Analysis Sensing 2023, 3, e202200087.

[43] C. Liu , T. Xu , D. Wang , X. Zhang , Talanta 2020, 212, 120801. 32113563 10.1016/j.talanta.2020.120801

[44] Z. Wu , Z. Qiao , S. Chen , S. Fan , Y. Liu , J. Qi , C. T. Lim , Commun. Mater. 2024, 5, 33.

[45] A. Choe , J. Yeom , R. Shanker , M. P. Kim , S. Kang , H. Ko , NPG Asia Mater. 2018, 10, 912.

[46] J.-H. Ha , Y. Jeong , J. Ahn , S. Hwang , S. Jeon , D. Kim , J. Ko , B. Kang , Y. Jung , J. Choi , H. Han , J. Gu , S. Cho , H. Kim , M. Bok , S. A. Park , J.-H. Jeong , I. Park , Mater. Horiz. 2023, 10, 4163. 37338170 10.1039/d3mh00340j

[47] X. Xue , Y. Nie , B. He , L. Xing , Y. Zhang , Z. L. Wang , Nanotechnology 2013, 24, 225501. 23633477 10.1088/0957-4484/24/22/225501

[48] M. Tonezzer , R. G. Lacerda , Phys. E Low‐Dimens. Syst. Nanostruct. 2012, 44, 1098.

[49] S. M. Sedghi , Y. Mortazavi , A. Khodadadi , Sens. Actuators B Chem. 2010, 145, 7.

[50] Y. Park , J. Jung , M. Chang , Appl. Sci. 2019, 9, 1070.

[51] S. M. A. Mokhtar , E. Alvarez de Eulate , M. Yamada , T. W. Prow , D. R. Evans , Med. Dev. Sens. 2021, 4, e10160.

[52] J. D. Mota‐Morales , E. Morales‐Narváez , Matter 2021, 4, 2141.

[53] Y. Qiao , X. Li , T. Hirtz , G. Deng , Y. Wei , M. Li , S. Ji , Q. Wu , J. Jian , F. Wu , Y. Shen , H. Tian , Y. Yang , T.-L. Ren , Nanoscale 2019, 11, 18923. 31532436 10.1039/c9nr05532k

[54] Y. R. Jeong , H. Park , S. W. Jin , S. Y. Hong , S.-S. Lee , J. S. Ha , Adv. Funct. Mater. 2015, 25, 4228.

[55] C. S. Boland , U. Khan , C. Backes , A. O’Neill , J. McCauley , S. Duane , R. Shanker , Y. Liu , I. Jurewicz , A. B. Dalton , J. N. Coleman , ACS Nano 2014, 8, 8819. 25100211 10.1021/nn503454h

[56] X. Li , T. Yang , Y. Yang , J. Zhu , L. Li , F. E. Alam , X. Li , K. Wang , H. Cheng , C.-T. Lin , Y. Fang , H. Zhu , Adv. Funct. Mater. 2016, 26, 1322.

[57] A. Palumbo , Z. Li , E.-H. Yang , IEEE Sens. J. 2022, 22, 20102.

[58] J. M. Nugent , K. S. V. Santhanam , A. Rubio , P. M. Ajayan , Nano Lett. 2001, 1, 87.

[59] J. R. Camargo , L. O. Orzari , D. A. G. Araújo , P. R. de Oliveira , C. Kalinke , D. P. Rocha , A. Luiz dos Santos , R. M. Takeuchi , R. A. A. Munoz , J. A. Bonacin , B. C. Janegitz , Microchem. J. 2021, 164, 105998.

[60] G. Volpes , S. Valenti , H. Zafar , R. Pernice , G. M. Stojanović , Flex. Print. Electron. 2023, 8, 015016.

[61] F. Gao , C. Liu , L. Zhang , T. Liu , Z. Wang , Z. Song , H. Cai , Z. Fang , J. Chen , J. Wang , M. Han , J. Wang , K. Lin , R. Wang , M. Li , Q. Mei , Z. Ma , S. Liang , G. Gou , N. Xue , Microsyst. Nanoeng. 2023, 9, 1. 36597511 10.1038/s41378-022-00443-6 PMC9805458

[62] E. Bakker , M. Telting‐Diaz , Anal. Chem. 2002, 74, 2781. 12090665 10.1021/ac0202278

[63] N. A. Rakow , K. S. Suslick , Nature 2000, 406, 710. 10963592 10.1038/35021028

[64] J. You , M. D. Willcox , M. C. Madigan , V. Wasinger , B. Schiller , B. J. Walsh , P. H. Graham , J. H. Kearsley , Y. Li , Adv. Clin. Chem. 2013, 62, 151. 24772667 10.1016/b978-0-12-800096-0.00004-4

[65] S. K. Nandi , D. Singh , J. Upadhay , N. Gupta , N. Dhiman , S. K. Mittal , N. Mahindroo , Int. J. Biol. Macromol. 2021, 193, 838. 34728300 10.1016/j.ijbiomac.2021.10.198

[66] X. Zhan , J. Li , Y. Guo , O. Golubnitschaja , EPMA J. 2021, 12, 449. 34876936 10.1007/s13167-021-00265-y PMC8639411

[67] L. Zhou , R. W. Beuerman , C. M. Chan , S. Z. Zhao , X. R. Li , H. Yang , L. Tong , S. Liu , M. E. Stern , D. Tan , J. Proteome Res. 2009, 8, 4889. 19705875 10.1021/pr900686s

[68] Z. Torok , T. Peto , E. Csosz , E. Tukacs , A. Molnar , Z. Maros‐Szabo , A. Berta , J. Tozser , A. Hajdu , V. Nagy , B. Domokos , A. Csutak , BMC Ophthalmol. 2013, 13, 40. 23919537 10.1186/1471-2415-13-40 PMC3770351

[69] C. Salvisberg , N. Tajouri , A. Hainard , P. R. Burkhard , P. H. Lalive , N. Turck , Proteomics Clin. Appl. 2014, 8, 185. 24488530 10.1002/prca.201300053

[70] A. Kenny , E. M. Jiménez‐Mateos , M. A. Zea‐Sevilla , A. Rábano , P. J. Gili‐Manzanaro , H. M. Prehn , D. C. Henshall , J. Ávila , T. Engel , F. Hernández , Sci. Rep. 2019, 9, 15437. 31659197 10.1038/s41598-019-51837-y PMC6817868

[71] N. Tomosugi , K. Kitagawa , N. Takahashi , S. Sugai , I. Ishikawa , J. Proteome Res. 2005, 4, 820. 15952728 10.1021/pr0497576

[72] C. Aass , I. Norheim , E. F. Eriksen , E. C. Børnick , P. M. Thorsby , M. Pepaj , Clin. Endocrinol. 2016, 85, 805. 10.1111/cen.13122 27256639

[73] J. Kim , M. Kim , M.-S. Lee , K. Kim , S. Ji , Y.-T. Kim , J. Park , K. Na , K.-H. Bae , H. K. Kim , F. Bien , C. Y. Lee , J.-U. Park , Nat. Commun. 2017, 8, 14997. 28447604 10.1038/ncomms14997 PMC5414034

[74] S. Guo , K. Wu , C. Li , H. Wang , Z. Sun , D. Xi , S. Zhang , W. Ding , M. E. Zaghloul , C. Wang , F. A. Castro , D. Yang , Y. Zhao , Matter 2021, 4, 969. 33398259 10.1016/j.matt.2020.12.002 PMC7773002

[75] S. Kim , H.-J. Jeon , S. Park , D. Y. Lee , E. Chung , Sci. Rep. 2020, 10, 8254. 32427894 10.1038/s41598-020-65103-z PMC7237479

[76] B. Lin , M. Wang , C. Zhao , S. Wang , K. Chen , X. Li , Z. Long , C. Zhao , X. Song , S. Yan , L. Wang , W. Ma , NPJ Flex. Electron. 2022, 6, 1.

[77] A. Kikuchi , K. Suzuki , O. Okabayashi , H. Hoshino , K. Kataoka , Y. Sakurai , T. Okano , Anal. Chem. 1996, 68, 823. 21619178 10.1021/ac950748d

[78] Y.-J. Lee , S. A. Pruzinsky , P. V. Braun , Langmuir 2004, 20, 3096. 15875835

[79] M. Elsherif , M.U. Hassan , A. K. Yetisen , H. Butt , ACS Nano 2018, 12, 5452. 29750502 10.1021/acsnano.8b00829 PMC6107296

[80] S. Park , J. Hwang , H.-J. Jeon , W. R. Bae , I.-K. Jeong , T. G. Kim , J. Kang , Y.-G. Han , E. Chung , D. Y. Lee , ACS Appl . Nano Mater. 2021, 4, 5198.

[81] J. C. Tsai , E. M. Kanner , Expert Opin. Emerg. Drugs 2005, 10, 109. 15757407 10.1517/14728214.10.1.109

[82] H. A. Quigley , A. T. Broman , Br. J. Ophthalmol. 2006, 90, 262. 16488940 10.1136/bjo.2005.081224 PMC1856963

[83] P. Vitish‐Sharma , A. G. Acheson , R. Stead , J. Sharp , A. Abbas , M. Hovan , C. Maxwell‐Armstrong , B. Guo , A. J. King , Acta Ophthalmol. 2018, 96, e242. 28391622 10.1111/aos.13456

[84] Z. Liu , G. Wang , C. Ye , H. Sun , W. Pei , C. Wei , W. Dai , Z. Dou , Q. Sun , C.-T. Lin , Y. Wang , H. Chen , G. Shen , Adv. Funct. Mater. 2021, 31, 2010991.

[85] Y. Zhang , Y. Chen , T. Man , D. Huang , X. Li , H. Zhu , Z. Li , Microsyst. Nanoeng. 2019, 5, 1. 31636929 10.1038/s41378-019-0078-x PMC6799840

[86] J. Kim , J. Park , Y.-G. Park , E. Cha , M. Ku , H. S. An , K.-P. Lee , M.-I. Huh , J. Kim , T.-S. Kim , D. W. Kim , H. K. Kim , J.-U. Park , Nat. Biomed. Eng. 2021, 5, 772. 33941897 10.1038/s41551-021-00719-8

[87] C. Yang , Q. Wu , J. Liu , J. Mo , X. Li , C. Yang , Z. Liu , J. Yang , L. Jiang , W. Chen , H. Chen , J. Wang , X. Xie , Nat. Commun. 2022, 13, 2556. 35581184 10.1038/s41467-022-29860-x PMC9114010

[88] T. H. Dohlman , J. B. Ciralsky , E. C. Lai , Curr. Opin. Allergy Clin. Immunol. 2016, 16, 487. 27585060 10.1097/ACI.0000000000000307

[89] Y. Ye , Y. Ge , Q. Zhang , M. Yuan , Y. Cai , K. Li , Y. Li , R. Xie , C. Xu , D. Jiang , J. Qu , X. Liu , Y. Wang , Adv. Sci. 2022, 9, 2104738. 10.1002/advs.202104738 PMC9036001 35195359

[90] B. Young , E. Eggenberger , D. Kaufman , Curr. Opin. Ophthalmol. 2012, 23, 497. 23047167 10.1097/ICU.0b013e328359045e

[91] H. P. N. Scholl , E. Zrenner , Surv. Ophthalmol. 2000, 45, 29. 10946080 10.1016/s0039-6257(00)00125-9

[92] C. Senger , R. Moreto , S. E. S. Watanabe , A. G. Matos , J. S. Paula , J. Glaucoma 2020, 29, 147. 31809397 10.1097/IJG.0000000000001422

[93] K. Kim , H. J. Kim , H. Zhang , W. Park , D. Meyer , M. K. Kim , B. Kim , H. Park , B. Xu , P. Kollbaum , B. W. Boudouris , C. H. Lee , Nat. Commun. 2021, 12, 1544. 33750806 10.1038/s41467-021-21916-8 PMC7943761

[94] F. Alam , S. R. Choudhury , A. H. Jalal , Y. Umasankar , S. Forouzanfar , N. Akter , S. Bhansali , N. Pala , Biosens. Bioelectron. 2018, 117, 818. 30096736 10.1016/j.bios.2018.06.054

[95] I. L. Kahán , E. Ottovay , Albrecht Von Graefes Arch. Für Klin. Exp. Ophthalmol. 1975, 194, 267. 10.1007/BF00414674 1079419

[96] N. Thomas , I. Lähdesmäki , B. A. Parviz , Sens. Actuators B Chem. 2012, 162, 128.

[97] R. C. Reid , S. D. Minteer , B. K. Gale , Biosens. Bioelectron. 2015, 68, 142. 25562741 10.1016/j.bios.2014.12.034

[98] A. Konda , A. Prakash , G. A. Moss , M. Schmoldt , G. D. Grant , S. Guha , ACS Nano 2020, 14, 6339. 32329337 10.1021/acsnano.0c03252

[99] C. Liu , P. C. Hsu , H. W. Lee , M. Ye , G. Zheng , N. Liu , W. Li , Y. Cui , Nat. Commun. 2015, 6, 6205. 25683688 10.1038/ncomms7205

[100] A. J. Cohen , M. Brauer , R. Burnett , H. R. Anderson , J. Frostad , K. Estep , K. Balakrishnan , B. Brunekreef , L. Dandona , R. Dandona , V. Feigin , G. Freedman , B. Hubbell , A. Jobling , H. Kan , L. Knibbs , Y. Liu , R. Martin , L. Morawska , C. A. Pope , H. Shin , K. Straif , G. Shaddick , M. Thomas , R. Dingenen , A. van Donkelaar , T. van Vos , C. J. L. Murray , M. H. Forouzanfar , Lancet 2017, 389, 1907. 28408086 10.1016/S0140-6736(17)30505-6 PMC5439030

[101] J. S. Puthumana , L. M. Ngaage , M. R. Borrelli , E. M. Rada , J. Caffrey , Y. Rasko , Bull. World Health Organ. 2021, 99, 439. 34108754 10.2471/BLT.20.279786 PMC8164180

[102] J. P. Langrish , X. Li , S. Wang , M. M. Y. Lee , G. D. Barnes , M. R. Miller , F. R. Cassee , N. A. Boon , K. Donaldson , J. Li , L. Li , N. L. Mills , D. E. Newby , L. Jiang , Environ. Health. Perspect. 2012, 120, 367. 22389220 10.1289/ehp.1103898 PMC3295351

[103] E. Moloney , S. O’Sullivan , T. Hogan , L. W. Poulter , C. M. Burke , Chest 2002, 121, 1806. 12065342 10.1378/chest.121.6.1806

[104] N. Fens , A. H. Zwinderman , M. P. van der Schee , S. B. de Nijs , E. Dijkers , A. C. Roldaan , D. Cheung , E. H. Bel , P. J. Sterk , Am. J. Respir. Crit. Care Med. 2009, 180, 1076. 19713445 10.1164/rccm.200906-0939OC

[105] S. Rauf , M. T. Vijjapu , M. A. Andrés , I. Gascón , O. Roubeau , M. Eddaoudi , K. N. Salama , ACS Appl. Mater. Interfaces 2020, 12, 29999. 32512994 10.1021/acsami.0c07532 PMC7467549

[106] S. Zhang , L. Li , Y. Lu , D. Liu , J. Zhang , D. Hao , X. Zhang , L. Xiong , J. Huang , Appl. Mater. Today 2022, 26, 101391.

[107] Y. Huo , M. Bu , Z. Ma , J. Sun , Y. Yan , K. Xiu , Z. Wang , N. Hu , Y.-F. Li , J. Colloid Interface Sci. 2022, 607, 2010. 34798709 10.1016/j.jcis.2021.09.189

[108] Q. Ding , H. Wang , Z. Zhou , Z. Wu , K. Tao , X. Gui , C. Liu , W. Shi , J. Wu , SmartMat 2023, 4, e1147.

[109] J. Yang , L. Rong , W. Huang , Z. Wu , Q. Ding , H. Zhang , Y. Lin , F. Li , C. Li , B.-R. Yang , K. Tao , J. Wu , View 2023, 4, 20220060.

[110] B. Li , Q. Tian , H. Su , X. Wang , T. Wang , D. Zhang , Sens. Actuators B Chem. 2019, 299, 126973.

[111] T. Liu , D. Qu , L. Guo , G. Zhou , G. Zhang , T. Du , W. Wu , Adv. Sens. Res. 2023, 3, 2300014.

[112] S. A. Iyengar , P. Srikrishnarka , S. K. Jana , M. R. Islam , T. Ahuja , J. S. Mohanty , T. Pradeep , ACS Appl. Electron. Mater. 2019, 1, 951.

[113] P. Srikrishnarka , R. M. Dasi , S. K. Jana , T. Ahuja , J. S. Kumar , A. Nagar , A. R. Kini , B. George , T. Pradeep , ACS Omega 2022, 7, 42926. 36467907 10.1021/acsomega.2c05017 PMC9713799

[114] X. Yao , L. Chen , Z. Luo , C. Ye , F. Liang , T. Yang , X. Liu , X. Tian , H. Bi , C. Wang , C. Cai , L. Lyu , X. Wu , Nano Select 2022, 3, 1168.

[115] J. He , P. Xiao , J. Shi , Y. Liang , W. Lu , Y. Chen , W. Wang , P. Théato , S.-W. Kuo , T. Chen , Chem. Mater. 2018, 30, 4343.

[116] U. Mogera , A. A. Sagade , S. J. George , G. U. Kulkarni , Sci. Rep. 2014, 4, 4103. 24531132 10.1038/srep04103 PMC3925946

[117] T. Pan , Z. Yu , F. Huang , H. Yao , G. Hu , C. Tang , J. Gu , ACS Appl. Mater. Interfaces 2023, 15, 28248. 37262400 10.1021/acsami.3c04283

[118] E. P. Cummins , M. J. Strowitzki , C. T. Taylor , Physiol. Rev. 2020, 100, 463. 31539306 10.1152/physrev.00003.2019

[119] P. Escobedo , M. D. Fernández‐Ramos , N. López‐Ruiz , O. Moyano‐Rodríguez , A. Martínez‐Olmos , I. M. Pérez de Vargas‐Sansalvador , M. A. Carvajal , L. F. Capitán‐Vallvey , A. J. Palma , Nat. Commun. 2022, 13, 72. 35013232 10.1038/s41467-021-27733-3 PMC8748626

[120] L. Liu , F. Hao , S. P. Morgan , R. Correia , A. Norris , S. Korposh , Sens. Bio‐Sens. Res. 2019, 22, 100254.

[121] M. J. Lefferts , M. R. Castell , Sens. Diagn. 2022, 1, 955.

[122] N. T. Brannelly , J. P. Hamilton‐Shield , A. J. Killard , Crit. Rev. Anal. Chem. 2016, 46, 490. 26907707 10.1080/10408347.2016.1153949

[123] J. P. Ong , A. Aggarwal , D. Krieger , K. A. Easley , M. T. Karafa , F. Van Lente , A. C. Arroliga , K. D. Mullen , Am. J. Med. 2003, 114, 188. 12637132 10.1016/s0002-9343(02)01477-8

[124] A. Pita , A. Fernandez‐Bustos , M. Rodes , J. Arranz , C. Fisac , N. Virgili , J. Soler , Y. Wakabayashi , J. Parenter. Enter. Nutr. 2004, 28, 315. 10.1177/0148607104028005315 15449570

[125] C. R. Bosoi , C. F. Rose , Metab. Brain Dis. 2009, 24, 95. 19104924 10.1007/s11011-008-9112-7

[126] A. Armuzzi , M. Candelli , M. A. Zocco , A. Andreoli , A. De Lorenzo , E. C. Nista , L. Miele , F. Cremonini , I. A. Cazzato , A. Grieco , G. Gasbarrini , A. Gasbarrini , Aliment. Pharmacol. Ther. 2002, 16, 1977. 12452932 10.1046/j.1365-2036.2002.01374.x

[127] D. K. Bandgar , S. T. Navale , S. R. Nalage , R. S. Mane , F. J. Stadler , D. K. Aswal , S. K. Gupta , V. B. Patil , J. Mater. Chem. C 2015, 3, 9461.

[128] M. Eising , C. E. Cava , R. V. Salvatierra , A. J. G. Zarbin , L. S. Roman , Sens. Actuators B Chem. 2017, 245, 25.

[129] W. Zhang , G. Li , C. She , A. Liu , J. Cheng , H. Li , S. Liu , C. Jing , Y. Cheng , J. Chu , Anal. Chim. Acta 2020, 1093, 115. 31735204 10.1016/j.aca.2019.09.049

[130] C. Wu , L. Han , J. Zhang , Y. Wang , R. Wang , L. Chen , Adv. Mater. Technol. 2022, 7, 2101247.

[131] T. N. Ly , S. Park , Sci. Rep. 2018, 8, 18030. 30575788 10.1038/s41598-018-36468-z PMC6303394

[132] A. Joshi , S. A. Gangal , S. K. Gupta , Sens. Actuators B Chem. 2011, 156, 938.

[133] A. Shahmoradi , A. Hosseini , A. Akbarinejad , N. Alizadeh , Anal. Chem. 2021, 93, 6706. 33881307 10.1021/acs.analchem.1c00171

[134] S. Abbas , W. Yi , S. Yoo , A. Khalid , Z. Bhalli , J. Si , X. Hou , Phys. Status Solidi A 2022, 219, 2100529.

[135] S. Freddi , A. V. Emelianov , I. I. Bobrinetskiy , G. Drera , S. Pagliara , D. S. Kopylova , M. Chiesa , G. Santini , N. Mores , U. Moscato , A. G. Nasibulin , P. Montuschi , L. Sangaletti , Adv. Healthcare Mater. 2020, 9, 2000377. 10.1002/adhm.202000377 32378358

[136] H. Fujita , M. Hao , S. Takeoka , Y. Miyahara , T. Goda , T. Fujie , Adv. Mater. Technol. 2022, 7, 2101486.

[137] G. Wu , H. Du , Y. L. Cha , D. Lee , W. Kim , F. Feyzbar‐Khalkhali‐Nejad , T.-S. Oh , X. Zhang , D.-J. Kim , Sens. Actuators B Chem. 2023, 375, 132858.

[138] G. Song , D. Jiang , J. Wu , X. Sun , M. Deng , L. Wang , C. Hao , J. Shi , H. Liu , Y. Tian , M. Chen , Chem. Eng. J. 2022, 440, 135979.

[139] T.-C. Wu , A. De Luca , Q. Zhong , X. Zhu , O. Ogbeide , D.-S. Um , G. Hu , T. Albrow‐Owen , F. Udrea , T. Hasan , NPJ 2D Mater. Appl. 2019, 3, 1.

[140] P. P. Ricci , O. J. Gregory , Sci. Rep. 2021, 11, 7185. 33785837 10.1038/s41598-021-86686-1 PMC8009942

[141] S. Wang , Y. Jiang , H. Tai , B. Liu , Z. Duan , Z. Yuan , H. Pan , G. Xie , X. Du , Y. Su , Nano Energy 2019, 63, 103829.

[142] S. Wang , H. Tai , B. Liu , Z. Duan , Z. Yuan , H. Pan , Y. Su , G. Xie , X. Du , Y. Jiang , Nano Energy 2019, 58, 312.

[143] P. Puchalska , P. A. Crawford , Cell Metab. 2017, 25, 262. 28178565 10.1016/j.cmet.2016.12.022 PMC5313038

[144] J. Wolfsdorf , M. E. Craig , D. Daneman , D. Dunger , J. Edge , W. Lee , A. Rosenbloom , M. Sperling , R. Hanas , Pediatr. Diabetes 2009, 10, 118. 19754623 10.1111/j.1399-5448.2009.00569.x

[145] D. L. Coleman , Proc. Natl. Acad. Sci. USA 1980, 77, 290.6928621

[146] C. N. Tassopoulos , D. Barnett , T. R. Fraser , Lancet 1969, 293, 1282. 10.1016/s0140-6736(69)92222-3 4182178

[147] J. P. Casazza , D. H. Sohn , K. S. Park , B. J. Song , Arch. Biochem. Biophys. 1994, 309, 111. 8117100 10.1006/abbi.1994.1092

[148] F. Bovey , J. Cros , B. Tuzson , K. Seyssel , P. Schneiter , L. Emmenegger , L. Tappy , Nutr. Diabetes 2018, 8, 1. 30201981 10.1038/s41387-018-0058-5 PMC6131485

[149] A. T. Güntner , S. Abegg , K. Königstein , P. A. Gerber , A. Schmidt‐Trucksäss , S. E. Pratsinis , ACS Sens. 2019, 4, 268. 30623644 10.1021/acssensors.8b00937

[150] X. Li , J. Pan , Y. Wu , H. Xing , Z. An , Z. Shi , J. Lv , F. Zhang , J. Jiang , D. Wang , R. P. S. Han , B. Su , Y. Lu , Q. Liu , Biosens. Bioelectron. 2023, 222, 114945. 36462428 10.1016/j.bios.2022.114945

[151] J. Dai , J. Meng , X. Zhao , W. Zhang , Y. Fan , B. Shi , Z. Li , Adv. Mater. Technol. 2023, 8, 2201535.

[152] M. Zhou , Y. Liu , Y. Duan , Clin. Chim. Acta 2012, 413, 1770. 22796631 10.1016/j.cca.2012.07.006

[153] S. Svensson , A.-C. Olin , M. Lärstad , G. Ljungkvist , K. Torén , J. Chromatogr. B 2004, 809, 199–203. 10.1016/j.jchromb.2004.06.027 15315765

[154] D. Maier , E. Laubender , A. Basavanna , S. Schumann , F. Güder , G. A. Urban , C. Dincer , ACS Sens. 2019, 4, 2945. 31610653 10.1021/acssensors.9b01403 PMC6879172

[155] Y. Cao , H. Shi , Y. Zheng , Z. Tan , Z. Xie , C. Zhang , Z. Chen , Sens. Actuators B Chem. 2023, 393, 134189.

[156] R. Hingson , M. Winter , Alcohol Res. Health, 2003, 27, 63. 15301401 PMC6676697

[157] M. Rezapour , K. Ksaibati , Humanit. Soc. Sci. Commun. 2022, 9, 1. 10.1057/s41599-022-01334-x PMC9491673 36164459

[158] R. Powers , M. Etezadi‐Amoli , E. M. Arnold , S. Kianian , I. Mance , M. Gibiansky , D. Trietsch , A. S. Alvarado , J. D. Kretlow , T. M. Herrington , S. Brillman , N. Huang , P. T. Lin , H. A. Pham , A. V. Ullal , Sci. Transl. Med. 2021, 13, eabd7865. 33536284 10.1126/scitranslmed.abd7865

[159] H. H. Mosher , J. Biol. Chem. 1933, 99, 781.

[160] P. J. Derbyshire , H. Barr , F. Davis , S. P. J. Higson , J. Physiol. Sci. 2012, 62, 429. 22678934 10.1007/s12576-012-0213-z PMC10717375

[161] S. Kanaparthi , P. Supraja , S. G. Singh , in Advanced Biosensors for Health Care Applications (eds. I. R. Khan , A. Mohammad , A. M. Asiri ), Elsevier, Amsterdam 2019, pp. 209–226.

[162] E. Ono , H. Murota , Y. Mori , Y. Yoshioka , Y. Nomura , T. Munetsugu , H. Yokozeki , I. Katayama , PLoS ONE 2018, 13, e0195960. 29677207 10.1371/journal.pone.0195960 PMC5909908

[163] T. Takemura , P. W. Wertz , K. Sato , Br. J. Dermatol. 1989, 120, 43. 2638916 10.1111/j.1365-2133.1989.tb07764.x

[164] K. Agrawal , L. A. Hassoun , N. Foolad , T. L. Pedersen , R. K. Sivamani , J. W. Newman , J. Lipid Res. 2017, 58, 188. 27875258 10.1194/jlr.M071738 PMC5234720

[165] J. Quartier , W. Rao , S. Slade , F. Métral , M. Lapteva , Y. N. Kalia , Int. J. Pharm. 2021, 607, 120967. 34352336 10.1016/j.ijpharm.2021.120967

[166] A. C. Kendall , M. M. Koszyczarek , E. A. Jones , P. J. Hart , M. Towers , C. E. M. Griffiths , M. Morris , A. Nicolaou , Exp. Dermatol. 2018, 27, 721. 29654617 10.1111/exd.13558

[167] C.-T. Huang , M.-L. Chen , L.-L. Huang , I.-F. Mao , Chin. J. Physiol. 2002, 45, 109. 12817713

[168] S. Salatiello , M. Spinelli , C. Cassiano , A. Amoresano , F. Marini , S. Cinti , Anal. Chim. Acta 2022, 1210, 339882. 35595367 10.1016/j.aca.2022.339882

[169] M. G. Bulmer , J. Physiol. 1957, 137, 261. 13449876 10.1113/jphysiol.1957.sp005810 PMC1362977

[170] S. Robinson , A. H. Robinson , Physiol. Rev. 1954, 34, 202. 13155186 10.1152/physrev.1954.34.2.202

[171] P. D. Di Santagnese , R. C. Darling , G. A. Perera , E. Shea , Pediatrics 1953, 12, 549. 13111855

[172] I. J. Lichton , J. Appl. Physiol. 1957, 11, 223. 13475170 10.1152/jappl.1957.11.2.223

[173] P. M. Quinton , Physiology 2007, 22, 212. 17557942 10.1152/physiol.00041.2006

[174] J. T. Korpelainen , K. A. Sotaniemi , V. V. Myllylä , Stroke 1992, 23, 1271. 1519281 10.1161/01.str.23.9.1271

[175] M. Wang , Y. Yang , J. Min , Y. Song , J. Tu , D. Mukasa , C. Ye , C. Xu , N. Heflin , J. S. McCune , T. K. Hsiai , Z. Li , W. Gao , Nat. Biomed. Eng. 2022, 6, 1225. 35970928 10.1038/s41551-022-00916-z PMC10432133

[176] E. Bilbao , O. Garate , T. Rodríguez Campos , M. Roberti , M. Mass , A. Lozano , G. Longinotti , L. Monsalve , G. Ybarra , Chemosensors 2023, 11, 244.

[177] X. Yang , J. Yi , T. Wang , Y. Feng , J. Wang , J. Yu , F. Zhang , Z. Jiang , Z. Lv , H. Li , T. Huang , D. Si , X. Wang , R. Cao , X. Chen , Adv. Mater. 2022, 34, 2201768. 10.1002/adma.202201768 36134533

[178] S. M. Mugo , W. Lu , M. Wood , S. Lemieux , Electrochem. Sci. Adv. 2022, 2, e2100039.

[179] X. Huang , Y. Liu , W. Park , J. Li , J. Ma , C. K. Yiu , Q. Zhang , J. Li , P. Wu , J. Zhou , Y. Zeng , X. He , J. Li , T. H. Wong , K. Yao , L. Zhao , Y. Gao , R. Shi , H. Li , M. Li , D. Li , Z. Zhao , Y. Li , H. Li , X. Yu , Adv. Healthcare Mater. 2023, 2202846.

[180] M. Slavich , G. Falasconi , A. Guarnaccia , L. Pannone , L. Rampa , G. Fragasso , A. Granata , S. Savonitto , R. Spoladore , Am. J. Cardiovasc. Dis. 2021, 11, 635. 34849296 PMC8611272

[181] A. Aggarwal , M. Dautta , L. F. Ayala‐Cardona , A. Wudaru , A. Javey , Adv. Mater. Technol. 2023, 8, 2300385.

[182] D.-H. Choi , M. Gonzales , G. B. Kitchen , D.-T. Phan , P. C. Searson , ACS Sens. 2020, 5, 3821. 33263987 10.1021/acssensors.0c01219

[183] M. Bariya , N. Davis , L. Gillan , E. Jansson , A. Kokkonen , C. McCaffrey , J. Hiltunen , A. Javey , ACS Sens. 2022, 7, 1156. 35411764 10.1021/acssensors.2c00177

[184] M. Dautta , L. F. Ayala‐Cardona , N. Davis , A. Aggarwal , J. Park , S. Wang , L. Gillan , E. Jansson , M. Hietala , H. Ko , J. Hiltunen , A. Javey , Adv. Mater. Technol. 2023, 8, 2201187.

[185] J. K. Sim , Y.-H. Cho , Sens. Actuators B Chem. 2016, 234, 176.

[186] A. Brueck , T. Iftekhar , A. B. Stannard , K. Yelamarthi , T. Kaya , Sensors 2018, 18, 533. 29439398 10.3390/s18020533 PMC5855985

[187] R. Ghaffari , D. S. Yang , J. Kim , A. Mansour , J. A. Wright , J. B. Model , D. E. Wright , J. A. Rogers , T. R. Ray , ACS Sens. 2021, 6, 2787. 34351759 10.1021/acssensors.1c01133 PMC9108092

[188] J. Choi , A. J. Bandodkar , J. T. Reeder , T. R. Ray , A. Turnquist , S. B. Kim , N. Nyberg , A. Hourlier‐Fargette , J. B. Model , A. J. Aranyosi , S. Xu , R. Ghaffari , J. A. Rogers , ACS Sens. 2019, 4, 379. 30707572 10.1021/acssensors.8b01218

[189] S. Kim , B. Lee , J. T. Reeder , S. H. Seo , S.-U. Lee , A. Hourlier‐Fargette , J. Shin , Y. Sekine , H. Jeong , Y. S. Oh , A. J. Aranyosi , S. P. Lee , J. B. Model , G. Lee , M.-H. Seo , S. S. Kwak , S. Jo , G. Park , S. Han , I. Park , H.-I. Jung , R. Ghaffari , J. Koo , P. V. Braun , J. A. Rogers , Proc. Natl. Acad. Sci. USA 2020, 117, 27906. 33106394 10.1073/pnas.2012700117 PMC7668081

[190] A. J. Bandodkar , W. J. Jeang , R. Ghaffari , J. A. Rogers , Annu. Rev. Anal. Chem. 2019, 12, 1. 10.1146/annurev-anchem-061318-114910 30786214

[191] S. Liu , D. S. Yang , S. Wang , H. Luan , Y. Sekine , J. B. Model , A. J. Aranyosi , R. Ghaffari , J. A. Rogers , EcoMat 2023, 5, e12270.

[192] A. Koh , D. Kang , Y. Xue , S. Lee , R. M. Pielak , J. Kim , T. Hwang , S. Min , A. Banks , P. Bastien , M. C. Manco , L. Wang , K. R. Ammann , K.-I. Jang , P. Won , S. Han , R. Ghaffari , U. Paik , M. J. Slepian , G. Balooch , Y. Huang , J. A. Rogers , Sci. Transl. Med. 2016, 8, 366ra165. 10.1126/scitranslmed.aaf2593 PMC5429097 27881826

[193] Z. Yuan , L. Hou , M. Bariya , H. Y. Y. Nyein , L.-C. Tai , W. Ji , L. Li , A. Javey , Lab Chip 2019, 19, 3179. 31433434 10.1039/c9lc00598f

[194] A. Ghoorchian , M. Kamalabadi , M. Moradi , T. Madrakian , A. Afkhami , H. Bagheri , M. Ahmadi , H. Khoshsafar , Anal. Chem. 2022, 94, 2263. 35050594 10.1021/acs.analchem.1c04960

[195] E. Garcia‐Cordero , F. Bellando , J. Zhang , F. Wildhaber , J. Longo , H. Guérin , A. M. Ionescu , ACS Nano 2018, 12, 12646. 30543395 10.1021/acsnano.8b07413

[196] Y. Lu , K. Jiang , D. Chen , G. Shen , Nano Energy 2019, 58, 624.

[197] Y. Gai , E. Wang , M. Liu , L. Xie , Y. Bai , Y. Yang , J. Xue , X. Qu , Y. Xi , L. Li , D. Luo , Z. Li , Small Methods 2022, 6, 2200653. 10.1002/smtd.202200653 36074976

[198] S. Y. Oh , S. Y. Hong , Y. R. Jeong , J. Yun , H. Park , S. W. Jin , G. Lee , J. H. Oh , S.-S. Lee , S.-S. Lee , J. S. Ha , ACS Appl. Mater. Interfaces 2018, 10, 13729. 29624049 10.1021/acsami.8b03342

[199] Y. J. Hong , H. Lee , J. Kim , M. Lee , H. J. Choi , T. Hyeon , D.-H. Kim , Adv. Funct. Mater. 2018, 28, 1805754.

[200] G. Xiao , J. He , X. Chen , Y. Qiao , F. Wang , Q. Xia , L. Yu , Z. Lu , Cellulose 2019, 26, 4553.

[201] A. Singh , A. Sharma , S. Arya , J. Anal. Sci. Technol. 2022, 13, 11.

[202] J. Zhang , Q. Zhou , J. Cao , W. Wu , H. Zhang , Y. Shi , Q. Mao , H. Ma , Cellulose 2021, 28, 3123.

[203] Y. Wang , F. Chen , J. Ye , H. Liu , T. Zhang , Z. Li , Cellulose 2023, 30, 5131.

[204] M. Dervisevic , M. Alba , L. Esser , N. Tabassum , B. Prieto‐Simon , N. H. Voelcker , ACS Appl. Mater. Interfaces 2022, 14, 2401. 34968024 10.1021/acsami.1c22383

[205] L. Klous , C. J. de Ruiter , S. Scherrer , N. Gerrett , H. A. M. Daanen , Eur. J. Appl. Physiol. 2021, 121, 803. 33355715 10.1007/s00421-020-04562-8 PMC7892530

[206] J. Bakker , M. W. Nijsten , T. C. Jansen , Ann. Intensive Care 2013, 3, 12. 23663301 10.1186/2110-5820-3-12 PMC3654944

[207] P. A. van Beest , L. Brander , S. P. A. Jansen , J. H. Rommes , M. A. Kuiper , P. E. Spronk , Ann. Intensive Care 2013, 3, 6. 23446002 10.1186/2110-5820-3-6 PMC3599274

[208] G. Rabost‐Garcia , V. Colmena , J. Aguilar‐Torán , J. Vieyra Galí , J. Punter‐Villagrasa , J. Casals‐Terré , P. Miribel‐Catala , X. Muñoz , J. Cadefau , J. Padullés , D. Brotons Cuixart , ACS Sens. 2023, 8, 1536. 37029741 10.1021/acssensors.2c02614 PMC10152482

[209] X. Xuan , C. Chen , A. Molinero‐Fernandez , E. Ekelund , D. Cardinale , M. Swarén , L. Wedholm , M. Cuartero , G. A. Crespo , ACS Sens. 2023, 8, 2401. 37289663 10.1021/acssensors.3c00708 PMC10294257

[210] L. J. Currano , F. C. Sage , M. Hagedon , L. Hamilton , J. Patrone , K. Gerasopoulos , Sci. Rep. 2018, 8, 15890. 30367078 10.1038/s41598-018-33565-x PMC6203741

[211] X. Xuan , C. Pérez‐Ràfols , C. Chen , M. Cuartero , G. A. Crespo , ACS Sens. 2021, 6, 2763. 34228919 10.1021/acssensors.1c01009 PMC8397467

[212] A. Wiorek , M. Parrilla , M. Cuartero , G. A. Crespo , Anal. Chem. 2020, 92, 10153. 32588617 10.1021/acs.analchem.0c02211 PMC7467422

[213] M. G. McDonell , J. Skalisky , E. Leickly , S. McPherson , S. Battalio , J. R. Nepom , D. Srebnik , J. Roll , R. K. Ries , Drug Alcohol Depend. 2015, 157, 184. 26475403 10.1016/j.drugalcdep.2015.10.004 PMC4663163

[214] S. H. Stewart , D. G. Koch , I. R. Willner , P. K. Randall , A. Reuben , Alcohol. Alcohol. 2013, 48, 83. 23015609 10.1093/alcalc/ags109 PMC3523385

[215] C. L. Crunelle , D. Cappelle , M. Yegles , M. De Doncker , P. Michielsen , G. Dom , A. L. N. van Nuijs , K. E. Maudens , A. Covaci , H. Neels , Anal. Bioanal. Chem. 2016, 408, 2019. 26549114 10.1007/s00216-015-9117-0

[216] A. P. Selvam , S. Muthukumar , V. Kamakoti , S. Prasad , Sci. Rep. 2016, 6, 23111. 26996103 10.1038/srep23111 PMC4800395

[217] S. Anastasova , B. Crewther , P. Bembnowicz , V. Curto , H. M. Ip , B. Rosa , G.-Z. Yang , Biosens. Bioelectron. 2017, 93, 139. 27743863 10.1016/j.bios.2016.09.038

[218] J. H. Yoon , S.-M. Kim , Y. Eom , J. M. Koo , H.-W. Cho , T. J. Lee , K. G. Lee , H. J. Park , Y. K. Kim , H.-J. Yoo , S. Y. Hwang , J. Park , B. G. Choi , ACS Appl. Mater. Interfaces 2019, 11, 46165. 31774642 10.1021/acsami.9b16829

[219] A. Searle , L. Kirkup , Physiol. Meas. 2000, 21, 271. 10847194 10.1088/0967-3334/21/2/307

[220] K.-Y. Chun , S. Seo , C.-S. Han , Adv. Mater. 2022, 34, 2110082. 10.1002/adma.202110082 35178764

[221] T. Kim , J. Park , J. Sohn , D. Cho , S. Jeon , ACS Nano 2016, 10, 4770. 26986477 10.1021/acsnano.6b01355

[222] Q. Wang , S. Ling , X. Liang , H. Wang , H. Lu , Y. Zhang , Adv. Funct. Mater. 2019, 29, 1808695.

[223] J.-C. Hsieh , Y. Li , H. Wang , M. Perz , Q. Tang , K. W. K. Tang , I. Pyatnitskiy , R. Reyes , H. Ding , H. Wang , J. Mater. Chem. B 2022, 10, 7260. 35678148 10.1039/d2tb00618a

[224] G. Li , J. Wu , Y. Xia , Y. Wu , Y. Tian , J. Liu , D. Chen , Q. He , J. Neural Eng. 2020, 17, 026001. 32000145 10.1088/1741-2552/ab71ea

[225] P. Leleux , J.-M. Badier , J. Rivnay , C. Bénar , T. Hervé , P. Chauvel , G. G. Malliaras , Adv. Healthc. Mater. 2014, 3, 490. 24106008 10.1002/adhm.201300311

[226] Y.-H. Chen , M. O. De Beeck , L. Vanderheyden , E. Carrette , V. Mihajlović , K. Vanstreels , B. Grundlehner , S. Gadeyne , P. Boon , C. van Hoof , Sensors 2014, 14, 23758. 25513825 10.3390/s141223758 PMC4299086

[227] A. Harati , A. Jahanshahi , Sens. Actuators Phys. 2021, 326, 112727.

[228] L. Yang , Q. Liu , Z. Zhang , L. Gan , Y. Zhang , J. Wu , Adv. Mater. Technol. 2022, 7, 2100612.

[229] G. Pei , J. Wu , D. Chen , G. Guo , S. Liu , M. Hong , T. Yan , Sensors 2018, 18, 3396. 30314263 10.3390/s18103396 PMC6211015

[230] F. M. Carvalho , P. Lopes , M. Carneiro , A. Serra , J. Coelho , A. T. de Almeida , M. Tavakoli , ACS Appl. Electron. Mater. 2020, 2, 3390.

[231] G. Chen , N. Matsuhisa , Z. Liu , D. Qi , P. Cai , Y. Jiang , C. Wan , Y. Cui , W. R. Leow , Z. Liu , S. Gong , K.-Q. Zhang , Y. Cheng , X. Chen , Adv. Mater. 2018, 30, 1800129. 10.1002/adma.201800129 29603437

[232] P. A. Lopes , D. Vaz Gomes , D. Green Marques , P. Faia , J. Góis , T. F. Patrício , J. Coelho , A. Serra , A. T. de Almeida , C. Majidi , M. Tavakoli , Adv. Healthc. Mater. 2019, 8, 1900234. 10.1002/adhm.201900234 31273945

[233] H.-L. Peng , J.-Q. Liu , H.-C. Tian , Y.-Z. Dong , B. Yang , X. Chen , C.-S. Yang , Sens. Actuators B Chem. 2016, 226, 349.

[234] I. Menon , P. Bagwe , K.B. Gomes , L. Balaji , R. Gala , M. N. Uddin , M. J. D’Souza , S. M. Zughair , Micromachines 2021, 12, 435. 33919925 10.3390/mi12040435 PMC8070939

[235] R. Nagarkar , M. Singh , H.X. Nguyen , S. Jonnalagadda , J. Drug Deliv. Sci. Technol. 2020, 59, 101923.

[236] E. Larrañeta , R. E. Lutton , A. D. Woolfson , R. F. Donnelly , Mater. Sci. Eng. R Rep. 2016, 104, 1.

[237] M. Azmana , S. Mahmood , A. R. Hilles , U. K. Mandal , K. A. S. Al‐Japairai , S. Raman , J. Drug Deliv. Sci. Technol. 2020, 60, 101877.

[238] Y. Zheng , R. Ye , X. Gong , J. Yang , B. Liu , Y. Xu , G. Nie , X. Xie , L. Jiang , Microsyst. Nanoeng. 2023, 9, 35. 36987502 10.1038/s41378-023-00515-1 PMC10040928

[239] A. K. Nayak , S. Dev , K. Pal , I. Banerjee , Bioelectron. Med. Dev. 2019, 393.

[240] S. Kusama , K. Saita , Y. Matsui , N. Kimura , H. Abe , S. Yoshida , M. Nishizawa , Nat. Commun. 2021, 12, 658. 33510169 10.1038/s41467-021-20948-4 PMC7843990

[241] H. T. T. Duong , N. W. Kim , T. Thambi , V. G. Phan , M. S. Lee , Y. Yin , J. H. Jeong , D. S. Lee , J. Control. Release 2018, 269, 225. 29154976 10.1016/j.jconrel.2017.11.025

[242] B. K. Meyer , M. A. Kendall , D. M. Williams , A. J. Bett , S. Dubey , R. C. Gentzel , D. Casimiro , A. Forster , H. Corbett , M. Crichton , S. B. Baker , R. K. Evans , A. Bhambhani , Vaccine X 2019, 2, 100030. 31384745 10.1016/j.jvacx.2019.100030 PMC6668242

[243] G. J. Fernando , J. Hickling , C. M. J. Flores , P. Griffin , C. D. Anderson , S. R. Skinner , C. Davies , K. Witham , M. Pryor , J. Bodle , S. Rockman , I. H. Frazer , Vaccine 2018, 36, 3779. 29779922 10.1016/j.vaccine.2018.05.053

[244] K. Kadonosono , A. Arakawa , S. Yamane , M. Inoue , T. Yamakawa , E. Uchio , Y. Yanagi , Ophthalmology 2015, 122, 123. 25200400 10.1016/j.ophtha.2014.07.027

[245] K. Kadonosono , S. Yamane , M. Inoue , T. Yamakawa , E. Uchio , Sci. Rep. 2018, 8, 1360. 29358594 10.1038/s41598-018-19747-7 PMC5778058

[246] H. Li , G. Wu , Z. Weng , H. Sun , R. Nistala , Y. Zhang , ACS Sens. 2021, 6, 2181. 34038108 10.1021/acssensors.0c02330

[247] Y. Cheng , X. Gong , J. Yang , G. Zheng , Y. Zheng , Y. Li , Y. Xu , G. Nie , X. Xie , M. Chen , C. Yi , L. Jiang , Biosens. Bioelectron. 2022, 203, 114026. 35114468 10.1016/j.bios.2022.114026

[248] O. Heifler , E. Borber , N. Harpek , M. Zverzhinetsky , V. Krivitsky , I. Gabriel , V. Fourman , D. Sherman , F. Patolsky , ACS Nano 2021, 15, 12019. 34157222 10.1021/acsnano.1c03310 PMC8397432

[249] K. Y. Goud , C. Moonla , R. K. Mishra , C. Yu , R. Narayan , I. Litvan , J. Wang , ACS Sens. 2019, 4, 2196. 31403773 10.1021/acssensors.9b01127

[250] R. K. Mishra , Y. Gouda , Z. Li , C. Moonla , M. A. Mohamed , F. Tehrani , H. Teymourian , J. Wang , J. Am. Chem. Soc. 2020, 142, 5991. 32202103 10.1021/jacs.0c01883

[251] S. R. Corrie , G. J. P. Fernando , M. L. Crichton , M. E. G. Brunck , C. D. Anderson , M. A. F. Kendall , Lab Chip 2010, 10, 2655. 20820632 10.1039/c0lc00068j

[252] D. A. Muller , S. R. Corrie , J. Coffey , P. R. Young , M. A. Kendall , ACS Anal. Chem. 2012, 84, 3262. 10.1021/ac2034387 22424552

[253] C. G Li , M. Dangol , C. Y. Lee , M. Jang , H. Jung , Lab Chip 2015, 15, 382. 25352059 10.1039/c4lc00937a

[254] Y. Wu , F. Tehrani , H. Teymourian , J. Mack , A. Shaver , M. Reynoso , J. Kavner , N. Huang , A. Furmidge , A. Duvvuri , Y. Nie , Lori M. Laffel , F. J. Doyle III , M. E. Patti , E. Dassau , J. Wang , N. A. Currás , Anal. Chem. 2022, 94, 8335. 35653647 10.1021/acs.analchem.2c00829 PMC9202557

[255] L. Sigcha , L. Borzì , F. Amato , I. Rechichi , C. Ramos‐Romero , A. Cárdenas , L. Gascó , G. Olmo , Expert Syst. Appl. 2023, 229, 120541.

[256] L. Deng , D. Yu , Found. Trends Signal Process. 2014, 7, 197.

