# Supplementary material for: Wearable Sensors for Physiological Condition and Activity Monitoring
Source: Small Sci. 2024 May 18;4(7):2300358. doi: 10.1002/smsc.202300358 (PMC11935081; doi:10.1002/smsc.202300358)
Supplement: Supplementary file 1 — Supplementary Material [file SMSC-4-2300358-s001.pdf]

Supporting Information
for

Wearable Sensors for Physiological Condition and Activity Monitoring

Pillalamarri Srikrishnarka, Joonas Haapasalo, Juan P. Hinestroza, Zhipei Sun, and Nonappa**

Table of contents

Table S1: A comparison of reported for teardrop glucose sensing analysis…………………………...S2

Table S2: A comparison of reported sensors for IOP sensing and analysis…………………………S2-S3

Table S3: A comparison of reported sensors for teardrop lactate sensing and analysis……………S3

Table S4: A comparison of various techniques for breath humidity sensing and analysis………….S3-S4

Table S5: A comparison of various techniques for breath CO_2 sensing and analysis……………….S5

Table S6: A comparison of various techniques for breath ammonia sensing and analysis………….S5-S6

Table S7: A comparison of various techniques for breath acetone sensing and analysis……………S6

Table S8: A comparison of various techniques for breath hydrogen peroxide sensing and analysis...S6

Table S9: A comparison of various techniques for sweat metabolite sensing and analysis………….S6-S9

Table 10: A comparison of various techniques for human activity monitoring……………………...S9-S10

References corresponding to the main text… …… ……………………………………….S10-S14

Table S1: A comparison of reported for teardrop glucose sensing analysis.

Substrates/materials/devices	Transduction method	Advantages	Limitations	Ref.	Sensitivity/ Response
Soft contact lens as substrate, graphene/Ag nanowires	Electrochemical	Fabricated on commercially available contact lenses. Wireless data transfer. High specificity towards sensing glucose. Allows a duration of 24 h continuous glucose and IOP measurements.	Additional molecular barriers may be required to prevent the formation of AgCl salts due to the reaction between the teardrop and Ag nanowires.	73	N.A.
PDMS substrate and MoS_2/Au electrodes	Electrochemical	Flexible substrate. Transparency of the lens prevent vision loss. Allows glucose and temperature measurement.	PDMS has low hydrophilicity and may result in protein accumulation.	74	48 % at 0.6 mM
CeO nanoparticle treated with glucose oxidase	Colorimetric	Simple fabrication process. Rapid detection of glucose.	Severe diabetic conditions might limit low glucose detection. Nanoparticle coating lowers the transmittance. Not reusable.	75	N.A.
PEDOT:PSS/Pt and glucose oxidase	Electrochemical	Self-powered glucose and Ca^{2+} sensor. Selective and accurate glucose detection	Metal electrodes contact causes discomfort to the eye.	76	N.A.
PVA/PBA/Pt electrode	Electrochemical	Non-enzymatic detection. Reproducible outputs of complex-cast Pt electrodes	Not evaluated on animal models.	77	0.004 (μA/min)(dl/mg)
2-hydroxyethyl methacrylate and 2-acrylamidophenyl boronic acid polymer in polystyrene colloidal template.	Optical	Doesn't require enzymes such as glucose oxidase. Reversible glucose sensing.	Slow kinetics	78	N.A.
Acrylamide, phenylboronic acid	Optical	Fast and facile preparation. Rapid response to low glucose concentration.	Expensive instruments for measurements.	79	12 nm mM^{-1}
Cerium oxide nanoparticles, glucose oxidase, PEO	Colorimetric	No external electronic circuit. Detection via image processing.	Transparency is lost due to coating.	80	N.A.

Table S2: A comparison of reported sensors for IOP sensing and analysis.

Substrate/materials/devices	Transduction method	Advantages	Limitations	Reference	Sensitivity/Response
Graphene/PDMS	Piezoelectric	No external power supply. Wire data transfer. Low-cost. Allows for a period of 24 h continuous monitoring.	The accuracy and stability of the transmitted signal need improvement.	84	1.0164 mV mm Hg^{-1} on a silicone eye, 3.166 mV mm Hg^{-1} in vitro on the porcine eye
Graphene woven fabric (GWF)	Electrical	Highly transparent. Biocompatible.	Poor repeatability and consistency between the fabricated GWF.	85	N.A.

			Long-term stability and repeatability need to be evaluated.		
Ag NW-NF/Si	Electrical	Transparent and stretchable. Wireless data transfer. Negligible corneal abrasion.	Extensive fabrication steps and cost-intensive to fabricate.	86	0.05% per mm Hg
Cu/Ni/au electrodes	Electrical	High optical transparency and closed-loop system sensing and drug delivery. Wireless data transmission. Cost-effective fabrication	Higher wireless frequencies heat up the sensor.	87	1.28 ± 0.09 MHz/mmHg
SiO2/Au nanobowls	Braggs diffraction and SERS	Dual sensing of IOP and MMP-9. No external power supply. Noninvasive health monitoring.	Low transmittance. Lower sensitivity	88	N.A.

Table S3: A comparison of reported sensors for teardrop lactate sensing and analysis.

**Substrate/material/devices**	**Transduction method**	**Advantages**	**Limitations**	**Ref.**	**Sensitivity /Response**
Lactate oxidase with glutaraldehyde and bovine serum albumin coated with medical grade polyurethane. Ti/Pd/Pt stack as electrode material	Electrochemical	Rapid response and high resolution. Stable for 24 h sensing and monitoring.	Lower stability due to poor current response after prolonged storage. Enzymes are unstable for long-term operation.	96	53 µA mM^{-1} cm^{-2}
Buckypaper anode with poly(methylene green) and a hydrogel matrix containing lactate dehydrogenase and nicotinamide adenine dinucleotide hydrate. Buckypaper cathode was modified with 1-pyrenemethyl anthracene-2-carboxylate, and then bilirubin oxidase	Electrochemical	Self-powered sensor. Contact lens biofuel. High stability.	Power loss after 48 h of operation. CNTs of buckypaper which could irritate the cornea.	97	N.A.

Table S4. A comparison of various techniques for exhaled breath humidity sensing and analysis

**Components /substrates/devices**	**Transduction method**	**Advantages**	**Limitations**	**Ref.**	**Sensitivity/Response**
MIL-96(Al) MOF coated on interdigitated textile	Electrochemical	High selectivity and thermal stability.	Slow response and recovery time	105	Response: 0.02
Ionically conductive MOFs	Electrochemical	Rapid response and recovery time, repeatability and high stability. Proximity and breath humidity sensing.	Biodegradable materials	106	36 (0-12 %RH) 477 (12-43 %RH) 4237 (36-61 %RH) 1537 (75-97 %RH)
Phytic acid-treated co-metal organic framework nanosheets	Electrochemical	Flexible, wide operating range, fast response. Proximity and exhaled breath humidity sensing.	Biodegradability	107	Response: 2000
PVA-CNF organohydrogel	Electrochemical	Flexible and rapid response and recovery time. Proximity and breath humidity sensing.	-	108	Response: 250
PAM coated fabric with LiBr salt	Electrochemical	Flame retarding, breathable, deformable and high sensitivity.	Slow response and recovery time	109	31197.8 $\%RH^{-1}$

In_2O_3 nanoparticles coated on graphene oxide	Electrochemical	Rapid recovery time, repeatability and high stability.	Slow response. Expensive raw materials	110	1061.6 pF $\%RH^{-1}$
MXene/TPU	Electrochemical	Fast response and recovery time. Wide operating range, low hysteresis and excellent repeatability.	Biocompatibility and biodegradability.	111	Response: 28%
PVDF/rGO/PANi	Electrochemical	High sensitivity, wide working range, fast response and recovery time. Low operating voltage and scalability.	Multiple fabrication steps and expensive raw materials	112	0.01 mA^{-1}
Fabric/PANi	Electrochemical	Affordable, fast response and recovery time.<br>Inert to bacterial growth and allows breath pattern recognition.	Miniaturization of electronics<br>Less accuracy due to less training set.	113	0.033 $\%RH^{-1}$
GO	Electrochemical	Flexible, quick response time, wide humidity operating range. Breath pattern recognition.	Slow recovery time	114	1.113 $\%RH^{-1}$
PDA/GO	Electrochemical	Rapid response and recovery time. Wide humidity operating range. Biocompatible materials. Allows skin humidity monitoring and speech recognition.	Air-breathability.	115	Response: 2000
Coronene tetracarboxylate and dodecyl methyl viologen	Electrochemical	Fast response and recovery time, wide operating range and high sensitivity.	Expensive raw materials.<br>Air-breathability.<br>Laborious fabrication steps.	116	Response: 45000
CNT/CNF	Electrochemical	High sensitivity, wide operation range. Robust in detecting different breathing patterns.	Slow response and recovery time. Expensive material.	117	Response: 61.5 %

Table S5. A comparison of various techniques for exhaled breath CO_2 sensing and analysis

**Substrates/materials/devices**	**Transduction method**	**Advantages**	**Limitations**	**Reference**	**Sensitivity**
TMAOH/CTAB/ La_2O_2:Eu	Colorimetric	Real-time CO_2 sensing. Wireless data transmission, energy harvesting via NFC and scalable.	Photobleaching could occur after prolonged illumination.	119	3.384 abs/%CO_2
Thymol blue, TEOS	Optical	Quick response time, wide operating range.	Slow recovery time and poor stability.	120	2.9 mg/mL

Table S6. A comparison of various techniques for exhaled breath ammonia sensing and analysis

**Substrates/materials/devices**	**Transduction method**	**Advantages**	**Limitations**	**Reference**	**Sensitivity**
PANi	Electrochemical	Sensing of 0-1000 ppm of ammonia concentration, long-term stability and flexibility.	Slow response and recovery time	127	22.5 Ω/ppm
PANi/CNT/HCSA	Electrochemical	Flexible with high stability, and repeatability	Slow response and recovery time.	128	0.34 Ω/ppm
PANi/Eu doped	Electrochemical	Rapid response and recovery time. High specificity, stability and robustness.		129	435% response for 0.3 mL

G.O./PANi	Electrochemical	Highly flexible. Specific for ammonia sensing.	Slow response and recovery time. Smaller working range of 0-100 ppm.	130	49.3 x 10^{-5} ppm^{-1}
RGO-conducting polymer	Electrochemical	Highly sensitive, portable, easy fabrication and room temperature operation.	The sensor was evaluated under a limited operating range	131	14 %/ppm
SRGO-PPY	Electrochemical	Flexible sensor, long-term stability and high repeatability.	Poor response and recovery time.	132	4.06 ppm^{-1}
SWCNTs	Electrochemical	High sensitivity and room temperature operation.	Expensive fabrication route	133	0.78% ppm
Paper/PEDOT:PSS and Iron (III) compound	Electrochemical	Highly flexible substrate and affordable.	Slow response and recovery time.	134	50% response
CNT/PANi	Electrochemical	High specificity, repeatable, room temperature sensing and long-term stability.	Slow response and recovery time.	135	Response: 452 %
Curcumin derivative	Colorimetric	Rapid response and recovery time and wide range of operation.	Expensive raw materials and preparation steps.	136	3 x 10^{-4} ppm^{-1}
WO3-graphene	Electrochemical	Very high responsivity and low power consumption.	Slow response and recovery time and high operating temperatures	137	125% ppm^{-1}
SnO on yttria-stabilized zirconia (YSZ) ribbons	Electrochemical	Highly specific.	High operating temperature and slow response and recovery time.	138	Response: 4.3%
Ce-ZnO/PET/Au	Triboelectric self-powered	Real-time breath ammonia sensing. Robust in distinguishing breathing patterns. Highly sensitive at lower ammonia concentrations. Self-powered ammonia sensor	Limited sensing range.	139	20.13 ppm^{-1}
Ce-doped ZnO/PANi	Triboelectric self-powered	No external power supply is needed. Capable of detecting breath ammonia during mouth ulcer.	Limited sensing range. Slow response and recovery time.	140	13.66 ppm^{-1}

Table S7. A comparison of various techniques for exhaled breath acetone sensing and analysis

**Substrates/materials/devices**	**Transduction method**	**Advantages**	**Limitations**	**Reference**	**Sensitivity**
$Ti_3C_2T_x$-MXene/TiO_2 peptide	Triboelectric self-powered	Incorporated inside face mask for breath acetone sensing. Allow breath acetone sensing before and after food consumption and exercise.	Slow response and recovery time.	150	Response: 3.15%
Na:ZnO/PVDF	Triboelectric self-powered	Flexible, external power supply not needed, and allows breathing pattern monitoring.	Slow response and recovery time	151	0.0075 ppm^{-1}

Table S8. A comparison of various techniques for exhaled breath hydrogen peroxide sensing and analysis

Substrates/materials/devices	Transduction method	Advantages	Limitations	Ref.	Sensitivity
Prussian blue/Ag/AgCl	Electrochemical	Affordable, lightweight and calibration-free paper-based electrochemical sensor, robust to detect different breathing patterns.	Slow response time and delay in analyte detection.	154	0.23 nA/µM
PANi/Prussian blue	Electrochemical	Quantitative detection of H_2O_2 in exhaled breath condensate. Portable sensor.	Slow response time, complex processing steps and limited repetitive use.	155	0.0089 µA/µM

Tables S9. A comparison of various techniques for sweat metabolite sensing and analysis

Metabolites	Components/materials/devices	Transduction methods	Advantages	Limitations	Ref.	Sensitivity /Response
Sweat rate	Commercial humidity sensor.	Electrical	Off-the-shelf humidity sensor.<br>Adhesion-free device, long-time operation for sweat rate monitoring.	The ejection of collected sweat needs improvement.	181	N.A.
	ITO electrodes deposited on PET substrate.	Electrical	Low-cost, easy-to-fabricate, and rapid response time.	Higher variation during high-intensity exercise.<br>Possible skin irritation due to tape adhesion.	182	0.3474 pF min^{-1}
	PET, Ag conductive tracks, C electrodes≥ PDMS reset button to expel collected sweat.	Electrochemical	Continuous sweat rate monitoring and expels the collected sweat.<br>Impedimetric sensing overcomes the interference from the ionic composition.	Multi-processing steps.<br>Signal strength over multiple reset cycles.<br>Tape adhesion might be uncomfortable.	183	N.A.
	PET substrate, Ag electrode. C electrode. N,N-bis(2-hydroxyethyl-3-aminopropyl)-triethoxysilane	Electrochemical	Tape-free sweat monitoring device. Reusable bands for holding the device.	Multi-processing steps for the fabrication.<br>Expensive. Poor ventilation at the device point of contact.	184	N.A.
	Commercial humidity sensor/microheater	Capacitive sensing	Natural ventilation, rapid response time and simple fabrication method.	Environmental wind flow might affect sweat evaporation.<br>Microheating elements might cause discomfort.<br>Mechanical moving parts may be subject to wear during the long run.	185	0.056 (pF/s)/(g/m^{2}h)
	Thermocouple	Calorimetric flow rate sensing	Rapid sensing, off-the-shelf components for sweat rate measurement.	The sensor might not be optimal for wearers who sweat less.<br>Visual data acquisition of sweat travel can have errors. The local sweat rate might represent the body sweat rate.	186	0.15 µL/min/cm^2
Sweat loss, pH, chloride, glucose, temperature, lactate	PMMA, PDMS. Silver chlor-anilate, glucose colorimetric assay. pH cocktail solution.	Colorimetric	Real-time detection of multicomponent detection, accurate, high selectivity and specificity<br>No mechanical moving parts.<br>No external power supply is needed.	Adhesion on the skin might cause discomfort.<br>The reusability of the sensor might be limited.	187	Lactate: -0.019 a.u/mM

Sweat rate, Glucose, Vitamin C	PDMS, PMMA, Cu electrodes, AuNPs, cortisol-BSA, IgG antibody	Conductivity, Fluorescence	Wireless data transfer, no external power supply and multimodal noninvasive sensing.	The detection range depends on the amount of analytes mobilized on the substrate. Reusability might be limited. Laborious fabrication steps.	188	N.A.
Sweat rate, Chloride and pH	KMPR, PTFE, PDMS, red cabbage powder, silver chloranilate and poly(hydroxyethyl methacrylate)	Colorimetric	Thermoplastic material enables scalable manufacturing and no external power supply needed.	The reusability of the sensor is limited.	189	N.A.
Lactate Chloride Glucose pH water	PDMS, glucose oxidase, lactase assay kit, chloride assay kit, pH indicator solution.		Soft and biocompatible materials, no patch detachment under temperature and humidity change. External power source not required.	Limited availability range of chemical reagents. During time-dependent changes in the marker, detection is limited due to the microfluidic channel design.	190	N.A.
Sweat rate, Na^+ and total ionic charge concentration	PDMS, PET, Na ionophore, Ag/AgCl electrode, APTES.	Electrochemical	Simultaneous sweat rate, total ionic concentration, and Na^+ concentration measurement. Continuous sensing and term sweat monitoring.	Expensive raw materials. Patch adhesion may cause discomfort to the user.	191	0.063 µS mM^{-1} of Na^+ sensing
Na+	$Na_{0.44}NnO_2$, Ag ink, C powder	Electrochemical	Low-cost, real-time Na^+ wearable sensor. Wireless sensor transfer via Bluetooth.	The sensor is also sensitive to Ca^{2+}. Pretreatment is done by soaking in $NaNO_3$ solution, which increases the analysis time.	192	Response: 58 mV dec^{-1}
pH, Na^+, K^+	Si wafer, HfO_2, ion-specific ionophore, Au electrodes	Electrochemical	Miniaturized low-volume passive microfluidic device for sweat monitoring with high sensitivity and specificity. Real-time sensing with repeatable values and low power consumption/sensor	Rigid device with extensive processing steps. High cost of fabrication.	193	35 mV/dec (pH), 62 mV/dec (Na^+), 55 mV/dec (K^+)
Glucose, Na^+, K^+	$NiCo_2O_4$, PVDF, Chitosan, Ag/AgCl, ion specific ionophore.	Electrochemical	Flexible substrate, an integrated device for real-time sweat monitoring. Wireless data transfer and self-powered sensor	Limited range of materials for ion specific sensing. Expensive sensing raw materials.	194	0.5 µA/µM (glucose), 0.031 nF/mM for (Na^+) and 0.056 nF/mM (K^+)
Na^+ and K^+ ions	PTFE, PEDOT:PSS, PET, ion-specific ionophore, MWCNTs	Electrochemical	Self-powered flexible wearable sensor. Wireless data transfer via Bluetooth.	Extensive fabrication route.	195	67.23 mV/dec (Na^+), 30.42 mV/dec (K^+)
Glucose and pH	AgNW, CoWO4/CNT electrode, Ag/AgCl, AuNS/CNT, PANi/CNT	Electrochemical	Skin attachable and stretchable wearable sensor. There is no interference from different analytes in sweat.	Performance can be affected by higher body temperature and expensive raw materials.	196	10.89 $\mu A/\mu Mcm^{2}$ (glucose), 71.44

						μA/μMcm[2] (pH)
Glucose	Conducting C paste, Prussian blue, Ag/AgCl counter electrodes.	Electrochemical	Disposable strip-based sensing with wireless data transfer. Integrated inside a smart band having SpO_2, temperature, and activity tracking devices.	Mandatory perspiration is needed for the detection of glucose.	197	N.A.
	Glucose oxidase, horseradish peroxidase, chitosan, paper, 3,3,5,5,-tetramethylbenzidine	Colorimetric	An affordable, wearable fabric-paper-based sensor with no external power supply is needed.	Repeated sensing might not be possible. Long-term stability needs to be evaluated along with long-term shelf-life	198	-36.72 R/μM
	Cu-Mn/polypyrrole treated cotton.	Electrochemical	Direct contact on the skin without any irritation on the skin. Continuous glucose monitoring for long periods of time. Stable and specific to glucose.	Further investigations are needed if polypyrrole leaches onto the skin after prolonged perspiration.	199	-2352 mA/μM
	CuNW/RGO/CF	Electrochemical	Flexible textile-based wearable sensor. Fast response and recovery time. Long-term stability, reproducibility and minimal interference.	Expensive raw materials. Extensive fabrication methods. Interference from other ions in sweat needs further analysis.	201	1020 $\mu AmM^{-1}cm^{-2}$
	Si Micropillar array/Au/Prussian blue/Chitosan-AuNPs/glucose oxidase	Electrochemical	Non-invasive sweat analysis platform with high stability and excellent fabrication reproducibility.	Delay in reaching the blood glucose concentrations. Extensive fabrication steps.	202	4.7 μAmM^{-1}
Sweat rate, lactate	Lactate oxidase.	Electrochemical	Establishes the relation between sweat and blood lactate concentration. A commercially available lateral flow strip was used for lactate sensing.	Continuous measurement is needed during the entire period of the workout. Renewal of sweat is needed.	203	0.0287 μAmM^{-1}
Lactate	C, Ag/AgCl electrodes, Nafion, lactose oxidase enzyme	Electrochemical	Wearable disposable electrochemical lactate sensor. Wireless data aquation and processing. Good response time	Manual collection of sweat is further needed under extreme exercise.	204	-12 nA mM^{-1}
	PEDOT:PSS electrodes, lactate oxidase immobilized in chitosan, Prussian blue	Electrochemical	Flexible, wearable lactate sensing device. Inexpensive fabrication technique.	Accumulation of hydrogen peroxide might degrade the electrode.	205	1.9 mA mM^{-1}
Lactate, pH and temperature	C, Ag/AgCl, Lactose oxidase, Prussian blue, PANi, MWCNTs, tetradodecylammonium tetrakis(4-chlorophenyl) borate (ETH 500), polyvinyl chloride (PVC), and bis(2-ethylhexyl) sebacate (DOS)	electrochemical	Simultaneous analyte sensing with good response time, repeatability, reversibility and excellent selectivity.	Extensive fabrication steps with expensive raw materials.	206	Lactate: -26.4 ± 0.7 nA mM^{-1}

Glucose, pH and temperature	C ink, Ag/AgCl, Prussian blue, glucose oxidase, Nafion, MWCNTs	Electrochemical	Reliable glucose sensing by measuring both pH and temperature and rapid response time.	Further evaluation is needed to attain a high correlation between sweat and blood glucose concentration.	207	N.A.
Ka+/Na+	citric acid, succinic acid, cyclohexanedimethanol, carbon fiber thread	Electrochemical	Self-healing, flexible, fast responsive sensor with wireless data transmission and real-time sweat monitoring. Selective to Na^{+}/K^{+} ions.	Long preparation steps and expensive raw materials.	208	60.7 mV/log[Na+] 54.8 mV/log[K+]

Table S10. A comparison of various techniques for human activity monitoring.

**Metabolites**	**Components/ materials/devices**	**Transduction**	**Advantages**	**Limitations**	**References**	**Sensitivity /Response**
B.P., ECG, EMG, MMG	PANi-PVC, PVDF-TrFe, C, 1-ethyl-3-methylimidazolium bis (trifluoromethyl sulfonyl) imide	Electrical	Flexible, compact, portable, adhere gel for multimodal sensing.	Slight response delay to the applied pressure.	220	0.97 VPa^{-1}
ECG	Carbon nanofillers, PDMS, CNT, graphene	Electrical	Flexible, metal-free conductive dry adhesive for ECG measurement. Accurate, robust against movement and underwater.	Expensive raw materials.	221	N.A.
ECG, breathing, temperature	Silk fibroin, Ca^{2+}, graphene	Electrical	Self-healing soft, flexible tattoo as a substrate, fast response and recovery time.	Under extensive perspiration, conductivity reduces.	222	2.09% $^{o}C^{-1}$
EEG	Ag/AgCl dry electrodes, Polyacetlyimide	Electrical	Sweat adsorbing sponge for EEG monitoring. Flexible dry electrodes compare EEG signals reliably compared to commercial wet electrodes. Nonpolarizable with good adhesive performance	Without perspiration, signal strength could be affected.	223	N.A.
	Au, Kapton, PEDOT:PSS	Electrical	Mechanically flexible with skin conformability to the skin. High signal-noise ratio	Long-term evaluation needs to understand the performance of the electrodes.	224	N.A.
ECG, EEG	Ethylene propylene diene, Au electrodes	Electrical	Easy fabrication, flexible and elastic conductive polymer electrodes. Long-term stability with good response	Long-term usage caused slight irritation due to poor ventilation of the fabric used. Low SNR when eyes were closed while measuring EEG	225	N.A.
EEG	Double-sided flex, Cu electrode	Electrical	Flexible, robust, skin conformable dry electrode. Performance even under harsh endurance testing	Movement artifacts and long-term electrode performance needed to be done.	226	N.A.

ECG, EEG	Polyacrylamide-acrylic acid-water-glycerol-based hydrogel	Electrical	Soft, hydrogel with excellent adhesion on the skin.<br>Lowers skin electrode impedance.<br>High SNR	-	229	N.A.
	Plasticized silk fibroin with Ca2+	Electrical	Tunable Young's modulus and stretchability.<br>Good biocompatibility and biodegradability.	Expensive raw materials	230	N.A.
ECG, EMG	PDMS, C, particles, Ag/AgCl, Ag/Ni coated PDMS	Electrical	Soft conductive electrodes are printable on the skin. High SNR. Reusable patch.	Expensive raw materials	231	N.A.

References:

[73] J. Kim, M. Kim, M. -S. Lee, K. Kim, S. Ji, Y. -T. Kim, J. Park, K. Na, K. -H. Bae, H. K. Kim, F. Bien, C. Y. Lee, J. -U. Park, *Nat. Commun.* **2017**, *8*, 14997.

[74] S. Guo, K. Wu, C. Li, H. Wang, Z. Sun, D. Xi, S. Zhang, W. Ding, M. E. Zaghloul, C. Wang, F. A. Castro, D. Yang, Y. Zhao, *Matter* **2021**, *4*, 969.

[75] S. Kim, H. -J. Jeon, S. Park, D. Y. Lee, E. Chung, *Sci. Rep.* **2020**, *10*, 8254.

[76] B. Lin, M. Wang, C. Zhao, S. Wang, K. Chen, X. Li, Z. Long, C. Zhao, X. Song, S. Yan, L. Wang, W. Ma, *Npj Flex. Electron.* **2022**, *6*, 1.

[77] A. Kikuchi, K. Suzuki, O. Okabayashi, H. Hoshino, K. Kataoka, Y. Sakurai, T. Okano, *Anal. Chem.* **1996**, *68*, 823.

[78] Y. -J. Lee, S. A. Pruzinsky, P. V. Braun, *Langmuir* **2004**, *20*, 3096.

[79] M. Elsherif, M.U. Hassan, A. K. Yetisen, H. Butt, *ACS Nano* **2018**, *12*, 5452.

[80] S.Park, J. Hwang, H. -J. Jeon, W. R. Bae, I. -K. Jeong, T. G.Kim, J. Kang, Y. -G. Han, E. Chung, D. Y. Lee, *ACS Appl. Nano Mater*. **2021**, *4*, 5198.

[84] Z. Liu, G. Wang, C. Ye, H. Sun, W. Pei, C. Wei, W. Dai, Z. Dou, Q. Sun, C. -T. Lin, Y. Wang, H. Chen, G. Shen, *Adv. Funct. Mater.* **2021**, *31*, 2010991.

[85] Y. Zhang, Y. Chen, T. Man, D. Huang, X. Li, H. Zhu, Z. Li, *Microsyst. Nanoeng.* **2019**, *5*, 1.

[86] J. Kim, J. Park, Y. -G. Park, E. Cha, M. Ku, H. S. An, K. -P. Lee, M. -I. Huh, J. Kim, T. -S. Kim, D. W. Kim, H. K. Kim, J. -U. Park, *Nat. Biomed. Eng.* **2021**, *5*, 772.

[87] C.Yang, Q. Wu, J. Liu, J. Mo, X. Li, C. Yang, Z. Liu, J. Yang, L. Jiang, W. Chen, H. Chen, J. Wang, X. Xie, *Nat. Commun.* **2022**, *13*, 2556.

[88] T. H. Dohlman, J. B. Ciralsky, E. C. Lai, *Curr. Opin. Allergy Clin. Immunol.* **2016**, *16*, 487.

[96] N. Thomas, I. Lähdesmäki, B. A. Parviz, *Sens. Actuators B Chem.* **2012**, *162*, 128.

[97] R. C. Reid, S. D. Minteer, B. K. Gale, *Biosens. Bioelectron.* **2015**, *68*, 142.

[105] S. Rauf, M. T. Vijjapu, M. A. Andrés, I. Gascón, O. Roubeau, M. Eddaoudi, K. N. Salama, K.N. *ACS Appl. Mater. Interfaces* **2020**, *12*, 29999.

[106] S. Zhang, L. Li, Y. Lu, D. Liu, J. Zhang, D. Hao, X. Zhang, L. Xiong, J. Huang, *Appl. Mater. Today* **2022**, *26*, 101391.

[107] Y. Huo, M. Bu, Z. Ma, J. Sun, Y. Yan, K. Xiu, Z. Wang, N. Hu, Y. -F. Li, *J. Colloid Interface Sci.* 2022, **607**, 2010.

[108] Q. Ding, H. Wang, Z. Zhou, Z. Wu, K. Tao, X. Gui, C. Liu, W. Shi, J. Wu, *SmartMat* **2023**, *4*, e1147.

[109] J. Yang, L. Rong, W. Huang, Z. Wu, Q. Ding, H. Zhang, Y. Lin, F. Li, C. Li, B. -R. Yang, K. Tao, J. Wu, *VIEW* **2023**, *4*, 20220060.

[110] B. Li, Q. Tian, H. Su, X. Wang, T. Wang, D. Zhang, *Sens. Actuators B Chem.* 2019, **299**, 126973.

[111] T. Liu, D. Qu, L. Guo, G. Zhou, G. Zhang, T. Du, W. Wu, *Adv. Sens. Res.*, **2023**, *3*, 2300014.

[112] S. A. Iyengar, P. Srikrishnarka, S. K. Jana, M. R. Islam, T. Ahuja, J. S. Mohanty, T. Pradeep, *ACS Appl. Electron. Mater.* **2019**, *1*, 951.

[113] P. Srikrishnarka, R. M. Dasi, S. K. Jana, T. Ahuja, J. S. Kumar, A. Nagar, A. R. Kini, B. George, T. Pradeep, *ACS Omega*, **2022**, *7*, 42926.

[114] X. Yao, L. Chen, Z. Luo, C. Ye, F. Liang, T. Yang, X. Liu, X. Tian, H. Bi, C. Wang, C. Cai, L. Lyu, X. Wu, *Nano Sel.* **2022**, *3*, 1168.

[115] J. He, P. Xiao, J. Shi, Y. Liang, W. Lu, Y. Chen, W. Wang, P. Théato, S. -W. Kuo, T. Chen, *Chem. Mater.* **2018**, *30*, 4343–4354.

[116] U. Mogera, A. A. Sagade, S. J. George, G. U. Kulkarni, *Sci. Rep.* **2014**, *4,* 4103.

[117] T. Pan, Z. Yu, F. Huang, H. Yao, G. Hu, C. Tang, J. Gu, *ACS Appl. Mater. Interfaces* **2023**, *15*, 28248–28257.

[119] P. Escobedo, M. D. Fernández-Ramos, N. López-Ruiz, O. Moyano-Rodríguez, A. Martínez-Olmos, I. M. Pérez de Vargas-Sansalvador, M. A. Carvajal, L. F. Capitán-Vallvey, A. J. Palma, *Nat. Commun.* **2022**, *13*, 72.

[120] L. Liu, F. Hao, S. P. Morgan, R. Correia, A. Norris, S. Korposh, *Sens. Bio-Sens. Res.* **2019**, *22*, 100254.

[127] D. K. Bandgar, S. T. Navale, S. R. Nalage, R. S. Mane, F. J. Stadler, D. K. Aswal, S. K. Gupta, V. B. Patil, *J. Mater. Chem. C* **2015**, *3*, 9461–9468.

[128] M. Eising, C. E. Cava, R. V. Salvatierra, A. J. G. Zarbin, L. S. Roman, *Sens. Actuators B Chem.* **2017**, *245*, 25–33.

[129] W. Zhang, G. Li, C. She, A. Liu, J. Cheng, H. Li, S. Liu, C. Jing, Y. Cheng, J. Chu, *Anal. Chim. Acta* **2020**, *1093*, 115–122.

[130] C. Wu, L. Han, J. Zhang, Y. Wang, R. Wang, L. Chen, *Adv. Mater. Technol.* **2022**, *7*, 2101247.

[131] T. N. Ly, S. Park, *Sci. Rep.* **2018**, *8*, 18030.

[132] A. Joshi, S. A. Gangal, S. K. Gupta, *Sens. Actuators B Chem.* **2011**, **1***56*, 938–942.

[133] A. Shahmoradi, A. Hosseini, A. Akbarinejad, N. Alizadeh, *Anal. Chem.* **2021**, *93*, 6706–6714.

[134] S. Abbas, W. Yi, S. Yoo, A. Khalid, Z. Bhalli, J. Si, X. Hou, *Phys. Status Solidi A*, **2022**, *219*, 2100529.

[135] S. Freddi, A. V. Emelianov, I. I. Bobrinetskiy, G. Drera, S. Pagliara, D. S. Kopylova, M. Chiesa, G. Santini, N. Mores, U. Moscato, A. G. Nasibulin, P. Montuschi, L. Sangaletti, *Adv. Healthc. Mater.* **2020,** *9*, 2000377.

[136] H. Fujita, M. Hao, S. Takeoka, Y. Miyahara, T. Goda, T. Fujie, *Adv. Mater. Technol.* **2022**, *7*, 2101486.

[137] G. Wu, H. Du, Y. L. Cha, D. Lee, W. Kim, F. Feyzbar-Khalkhali-Nejad, T. -S. Oh, X. Zhang, D. -J. Kim, *Sens. Actuators B Chem.* **2023**, *375*, 132858.

[138] G. Song, D. Jiang, J. Wu, X. Sun, M. Deng, L. Wang, C. Hao, J. Shi, H. Liu, Y. Tian, M. Chen, *Chem. Eng. J.* **2022**, *440*, 135979.

[139] T. -C. Wu, A. De Luca, Q. Zhong, X. Zhu, O. Ogbeide, D. -S. Um, G. Hu, T. Albrow-Owen, F. Udrea, T. Hasan, *Npj 2D Mater. Appl.* **2019**, *3*, 1–10.

[140] P. P. Ricci, O. J. Gregory, *Sci. Rep.* **2021**, *11*, 7185.

[150] X. Li, J. Pan, Y. Wu, H. Xing, Z. An, Z. Shi, J. Lv, F. Zhang, J. Jiang, D. Wang, R. P. S. Han, B. Su, Y. Lu, Q. Liu, *Biosens. Bioelectron.* **2023**, *222*, 114945.

[151] J. Dai, J. Meng, X. Zhao, W. Zhang, Y. Fan, B. Shi, Z. Li, *Adv. Mater. Technol.* **2023**, *8*, 2201535.

[154] D. Maier, E. Laubender, A. Basavanna, S. Schumann, F. Güder, G. A. Urban, C. Dincer, *ACS Sens.* **2019**, *4*, 2945–2951.

[155] Y. Cao, H. Shi, Y. Zheng, Z. Tan, Z. Xie, C. Zhang, Z. Chen, *Sens. Actuators B Chem.* **2023**, *393*, 134189.

[181] A. Aggarwal, M. Dautta, L. F. Ayala-Cardona, A. Wudaru, A. Javey, *Adv. Mater. Technol.* **2023**, *8*, 2300385.

[182] D. -H. Choi, M. Gonzales, G. B. Kitchen, D. -T. Phan, P. C. Searson, *ACS Sens.* **2020**, *5*, 3821–3826.

[183] M. Bariya, N. Davis, L. Gillan, E. Jansson, A. Kokkonen, C. McCaffrey, J. Hiltunen, A. Javey, *ACS Sens.* **2022**, *7*, 1156–1164.

[184] M. Dautta, L. F. Ayala-Cardona, N. Davis, A. Aggarwal, J. Park, S. Wang, L. Gillan, E. Jansson, M. Hietala, H. Ko, J. Hiltunen, A. Javey, *Adv. Mater. Technol.* **2023**, *8*, 2201187.

[185] J. K. Sim, Y. -H. Cho, *Sens. Actuators B Chem.* **2016**, 234, 176–183.

[186] A. Brueck, T. Iftekhar, A. B. Stannard, K. Yelamarthi, T. Kaya, *Sensors* **2018**, *18*, 533.

[187] R. Ghaffari, D. S. Yang, J. Kim, A. Mansour, J. A. Wright, J. B. Model, D. E. Wright, J. A. Rogers, T. R. Ray, *ACS Sens.* **2021**, *6*, 2787–2801.

[188] J. Choi, A. J. Bandodkar, J. T. Reeder, T. R. Ray, A. Turnquist, S. B. Kim, N. Nyberg, A. Hourlier-Fargette, J. B. Model, JA. J. Aranyosi, S. Xu, R. Ghaffari, J. A. Rogers, *ACS Sens.* **2019**, *4*, 379–388.

[189] S. Kim, B. Lee, J. T. Reeder, S. H. Seo, S. -U. Lee, A. Hourlier-Fargette, J. Shin, Y. Sekine, H. Jeong, Y. S. Oh, A. J. Aranyosi, S. P. Lee, J. B. Model, G. Lee, M. -H. Seo, S. S. Kwak, S. Jo, G. Park, S. Han, I. Park, H. -I. Jung, R. Ghaffari, J. Koo, P. V. Braun, J. A. Rogers, J.A. *Proc. Natl. Acad. Sci.* **2020**, *117*, 27906–27915.

[190] A. J. Bandodkar, W. J. Jeang, R. Ghaffari, J. A. Rogers, *Annu. Rev. Anal. Chem.* **2019**, *12*, 1–22.

[191] S. Liu, D. S. Yang, S. Wang, H. Luan, Y. Sekine, J. B. Model, A. J. Aranyosi, R. Ghaffari, J. A. Rogers, *EcoMat* **2023**, *5*, e12270.

[192] A. Koh, D. Kang, Y. Xue, S. Lee, R. M. Pielak, J. Kim, T. Hwang, S. Min, A. Banks, P. Bastien, M. C. Manco, L. Wang, K. R. Ammann, K. -I. Jang, P. Won, S. Han, R. Ghaffari, U. Paik, M. J. Slepian, G. Balooch, Y. Huang, J. A. Rogers, *Sci. Transl. Med.* **2016**, *8,* 366ra165.

[193] Z. Yuan, L. Hou, M. Bariya, H. Y. Y. Nyein, L. -C. Tai, W. Ji, L. Li, A. Javey, *Lab. Chip* **2019**, *19,* 3179–3189.

[194] A. Ghoorchian, M. Kamalabadi, M. Moradi, T. Madrakian, A. Afkhami, H. Bagheri, M. Ahmadi, H. Khoshsafar, *Anal. Chem.* **2022**, *94*, 2263–2270.

[195] E. Garcia-Cordero, F. Bellando, J. Zhang, F. Wildhaber, J. Longo, H. Guérin, A. M. Ionescu, *ACS Nano* **2018**, *12*, 12646–12656.

[196] Y. Lu, K. Jiang, D. Chen, G. Shen, *Nano Energy*, **2019**, *58*, 624–632.

[197] Y. Gai, E. Wang, M. Liu, L. Xie, Y. Bai, Y. Yang, J. Xue, X. Qu, Y. Xi, L. Li, D. Luo, Z. Li, *Small Methods* 2022, **6** (10), 2200653.

[198] S. Y. Oh, S. Y. Hong, Y. R. Jeong, J. Yun, H. Park, S. W. Jin, G. Lee, J. H. Oh, S. -S. Lee, S. -S. Lee, J. S. Ha, *ACS Appl. Mater. Interfaces* **2018**, *10*, 13729–13740.

[199] Y. J. Hong, H. Lee, J. Kim, M. Lee, H. J. Choi, T. Hyeon, D. -H. Kim, *Adv. Funct. Mater.* **2018**, *28*, 1805754.

[200] G. Xiao, J. He, X. Chen, Y. Qiao, F. Wang, Q. Xia, L. Yu, Z. Lu, *Cellulose* **2019**, *26*, 4553–4562.

[201] A. Singh, A. Sharma, S. Arya, S. *J. Anal. Sci. Technol.* **2022**, *13*, 11.

[202] J. Zhang, Q. Zhou, J. Cao, W. Wu, H. Zhang, Y. Shi, Q. Mao, H. Ma, *Cellulose* **2021**, *28*, 3123–3133.

[203] Y. Wang, F. Chen, J. Ye, H. Liu, T. Zhang, Z. Li, (2023) Reduced graphene oxide cotton fabric based on copper nanowires for flexible non-enzyme glucose sensor. *Cellulose* **2023**, *30*, 5131–5143.

[204] M. Dervisevic, M. Alba, L. Esser, N. Tabassum, B. Prieto-Simon, N. H. Voelcker, *ACS Appl. Mater. Interfaces* **2022**, *14*, 2401–2410.

[205] L. Klous, C. J. de Ruiter, S. Scherrer, N. Gerrett, H. A. M. Daanen, *Eur. J. Appl. Physiol.* **2021**, *121*, 803–816.

[206] J. Bakker, M. W. Nijsten, T. C. Jansen, T. C. *Ann. Intensive Care* **2013**, *3*, 12.

[207] P. A. van Beest, L. Brander, S. P. A. Jansen, J. H. Rommes, M. A. Kuiper, P. E. Spronk, *Ann. Intensive Care* **2013**, *3*, 6.

[208] G. Rabost-Garcia, V. Colmena, J. Aguilar-Torán, J. Vieyra Galí, J. Punter-Villagrasa, J. Casals-Terré, P. Miribel-Catala, X. Muñoz, J. Cadefau, J. Padullés, D. Brotons Cuixart, *ACS Sens.* **2023**, *8*, 1536–1541.

[221] T. Kim, J. Park, J. Sohn, D. Cho, S. Jeon, *ACS Nano* **2016**, *10*, 4770–4778.

[222] Q. Wang, S. Ling, X. Liang, H. Wang, H. Lu, Y. Zhang, *Adv. Funct. Mater.* **2019**, *29*, 1808695.

[223] J. -C. Hsieh, Y. Li, H. Wang, M. Perz, Q. Tang, K. W. K. Tang, I. Pyatnitskiy, R. Reyes, H. Ding, H. Wang, *J. Mater. Chem. B* **2022**, *10*, 7260–7280.

[224] G. Li, J. Wu, Y. Xia, Y. Wu, Y. Tian, J. Liu, D. Chen, Q. He, *J. Neural Eng.* **2020**, *17*, 026001.

[225] P. Leleux, J. -M. Badier, J. Rivnay, C. Bénar, T. Hervé, P. Chauvel, G. G. Malliaras, *Adv. Healthc. Mater.* **2014**, *3*, 490–493.

[226] Y. -H. Chen, M. O. De Beeck, L. Vanderheyden, E. Carrette, V. Mihajlović, K. Vanstreels, B. Grundlehner, S. Gadeyne, P. Boon, C. van Hoof, *Sensors* **2014**, *14*, 23758–23780.

[227] A. Harati, A. Jahanshahi, *Sens. Actuators Phys.* **2021**, *326*, 112727.

[228] L. Yang, Q. Liu, Z. Zhang, L. Gan, Y. Zhang, J. Wu, *Adv. Mater. Technol.* **2022**, *7*, 2100612.

[229] G. Pei, J. Wu, D. Chen, G. Guo, S. Liu, M. Hong, T. Yan, *Sensors* **2018**, *18*, 3396.

[230] F. M. Carvalho, P. Lopes, M. Carneiro, A. Serra, J. Coelho, A. T. de Almeida, M. Tavakoli, *ACS Appl. Electron. Mater.* **2020**, *2*, 3390–3401.

[231] G. Chen, N. Matsuhisa, Z. Liu, D. Qi, P. Cai, Y. Jiang, C. Wan, Y. Cui, W. R. Leow, Z. Liu, S. Gong, K. -Q. Zhang, Y. Cheng, X. Chen, *Adv. Mater.* **2018**, *30*, 1800129.
